# Modelling predictive gender- and gestation-specific weight reference centiles for preterm infants using a population-based cohort study

**DOI:** 10.1038/s41598-020-60895-6

**Published:** 2020-03-04

**Authors:** W. John Watkins, Daniel Farewell, Sujoy Banerjee, Hesham Nasef, Anitha James, Mallinath Chakraborty

**Affiliations:** 1 Department of Infection and Immunity, Cardiff University, Cardiff, UK; 2 Neonatal Intensive Care Unit, Singleton Hospital, Swansea, UK; 3 Neonatal Intensive Care Unit, Royal Gwent Hospital, Newport, UK; 4 Regional Neonatal Intensive Care Unit, University Hospital of Wales, Cardiff, UK; 5 Centre for Medical Education, School of Medicine, Cardiff University, Cardiff, UK

**Keywords:** Neonatology, Epidemiology

## Abstract

We aimed to model longitudinal data to create predictive growth charts for weight in preterm infants from birth till discharge, that took into account the differing growth rates post-birth when compared to in-utero growth and therefore was more representative of the data than the UK1990 reference charts. Data from birth until discharge (or death), was collected and rigorously cleaned for all infants born at <32 weeks of gestation over a 4-year period. Means and standard deviations from the UK1990 reference charts were used to compute standard deviation scores (SDS) for our cohort. 2/3rd of the data was randomly selected and used to create gestation and gender-specific predictive weight centile lines through novel application of mixed modelling methods. The remaining 1/3rd of the data was used to test model fit by comparing expected *vs* actual weights for the new model with those predicted by the UK1990 model. Data from 1,510 preterm infants was analysed. 1067 of these were used to produce the predictive model. Weekly SDS were significantly lower than predicted throughout hospital stay for all gestation groups when compared with UK1990 data. The test data (n = 539) fitted the new centile lines substantially better than those modelled by the UK1990 centile lines. Mixed modelling of longitudinal data produced new predictive references for weight centiles of preterm infants. A large population-based prospective study is needed to produce representative longitudinal reference growth charts using these methods.

## Introduction

New infant growth charts were adopted in the UK in May 2009, based on a large international study by the World Health Organisation, for monitoring growth of children between 0–4 years of age^1^. However, for preterm infants, old UK1990 reference curves^2,3^ continued to be used as recommended by the Scientific Advisory Committee on Nutrition (SACN) in 2007. The Royal College of Paediatrics and Child Health in the UK (RCPCH) combined these two datasets to produce new Neonatal Infant Close Monitoring Charts (NICM) for boys and girls^4^ the current standard for growth monitoring in preterm infants in the UK. The UK1990 reference curves plot cross-sectional data at birth, an approach that has been used elsewhere for reference standards^5^. Growth charts constructed using cross sectional reference data of weight at birth reflect expected or optimum *in utero* growth rather than actual growth after preterm birth. Ideally, growth chart references should be based on longitudinal data from a reference population of ‘healthy’ infants. However, the majority of preterm population born at gestations <32 weeks are challenged by multiple additional morbidities and it is unlikely that any of these infants can be classed as “healthy”. Growth of these infants is profoundly affected by the direct or indirect cumulative impact of multiple morbidities, not all exclusively linked to nutritional limitations^6^. More likely, preterm infants represent a different population compared to fetuses of corresponding gestations^7^ and it is unrealistic that very preterm newborns will follow the intrauterine growth trajectory using current methods of nutrition delivery. A review of the current literature reveals that no longitudinal reference centile charts exist to monitor the most vulnerable preterm infants born at extremes of gestation, who are at the highest risk of having growth problems^8^. This is a specific gap we have aimed to fulfil: a well-designed longitudinal growth chart from a large data set that represents the actual preterm population and contemporary clinical practice that is likely to better represent their true growth potential. Variations in nutrition delivery may be expected to be smoothed out in such a large population data set.

We aimed to achieve this objective by collecting retrospective, longitudinal measurements of weight from preterm infants born at <32 weeks gestation in Wales, starting from birth, and model gender- and gestation specific predictive centile charts till discharge from the neonatal unit.

## Methods

### Data collection

Retrospective anonymised data was collected for all infants born at <32 weeks of gestation in the calendar years 2011–2014 from the ‘Badgernet’ electronic neonatal database (Clevermed Systems, Edinburgh, UK) for Wales. The database maintains clinical records for all preterm infants admitted to the neonatal units in Wales from 2011. All infants born during the study period were included in the data cleaning process. Reasons for necessary exclusions are detailed in the data cleaning section. The postnatal weight of these infants was recorded in the database according to practices in different neonatal units and varied from daily to once in 2 weeks. Along with weight, data was also collected on duration of mechanical ventilation (days), respiratory support (days) and parenteral nutrition (days). Data was collected from all infants from birth till discharge from the neonatal unit. Infants who died before discharge contributed data up to the point of their stay on the unit (Table 1 and Supplementary Fig. 2). Stay duration was variable due to the differences in morbidities among infants but tended to be longer for the earlier gestation groups (Table 2).

**Table 1:** Demographic details of the whole cohort, stratified by gestation bands.

	23^+0^–25^+6^ weeks	26^+0^–28^+6^ weeks	29^+0^–31^+6^ weeks
Total Infants	183	454	910
Gender – Male (%)	98 (53.6%)	247 (54.4%)	494 (54.3%)
Deaths (%)	76 (41.5%)	82 (18.1%)	154 (16.9%)
Mean Birthweight (grams) ± 95% CI	670 (649–690)	1014 (994–1034)	1465 (14445–1485)
Birthweight SDS ± 95% CI – to do	0.01 (−0.13 to 0.15)	0.0 (−0.1 to 0.1)	0.08 (0.01 to 0.15)
Mean Stay duration (days) ± 95% CI	74 (66–82)	73 (70–77)	43 (41–44)
Mean Discharge weight (grams) ± 95% CI	2154 (1969–2340)	2526 (2442–2612)	2310 (2271–2349)
Discharge SDS ± 95% CI – to do	−1.0 (−1.2 to −0.8)	−1.0 (−1.1 to −0.9)	−0.9 (−1.0 to −0.8)
Mean Ventilation (days) ± 95% CI	27.2(17.3–37.2)	9.6 (8.5–10.7)	2.4 (1.9–2.9)
Mean Respiratory Support (days) ± 95% CI	54.1 (43.3–65.0)	40.2 (37.4–43.0)	11.8 (10.6–12.9)
Mean Parenteral Nutrition (days) ± 95% CI	23.1 (13.0–33.1)	16.2 (14.6–17.9)	8.9 (8.0–9.7)

(SDS = standard deviation score, CI = confidence interval).

**Table 2:** Demographic details, stratified by model- and test-groups, of the whole cohort which was analysed for the study.

Category	23^+0^–25^+6^				26^+0^–28^+6^				29^+0^–31^+6^			
	Male		Female		Male		Female		Male		Female	
	Model	Test	Model	Test	Model	Test	Model	Test	Model	Test	Model	Test
Total Infants	63	35	61	24	176	71	155	52	328	166	282	134
Deaths	16 (25%)	8 (23%)	11 (18%)	3 (12%)	9 (5%)	3 (4%)	8 (5%)	1 (2%)	8 (3%)	2 (1%)	7 (3%)	1 (1%)
Mean Gestation (weeks)	24.8 (0.08)	24.7 (0.10)	24.9 (0.10)	24.6 (0.16)	27.4 (0.06)	27.6 (0.10)	27.5 (0.08)	27.5 (0.12)	30.7* (0.05)	30.5* (0.07)	30.6* (0.05)	30.8* (0.07)
Mean Birthweight (grams)	718 (16.4)	766.2 (25.3)	605 (14.3)	566 (14.4)	1024 (16.8)	1079.9 (25.0)	984 (17.5)	975.4 (27.5)	1502 (18.3)	1467 (21.4)	1413 (17.2)	1478.4 (26.4)
Mean Stay duration (days)	70.1 (7.0)	73.1 (9.7)	76.0 (6.7)	74.7 (10.6)	76.0 (3.1)	76.7 (4.2)	67.8 (2.8)	75.8 (4.7)	43.1 (1.4)	45.0 (1.8)	42.7 (1.4)	40.0 (2.0)
Mean Discharge Weight (grams)	2141 (169.1)	2258 (236)	2152.0 (151.5)	2049 (238)	2625.6 (75.6)	2724 (121.5)	2349.4 (64.1)	2472.8 (101.7)	2398 (36.5)	2322.8 (35.6)	2225.2* (32.1)	2257.9* (61.2)
Mean Ventilation (days)	36.3 (14.3)	22.6 (3.2)	23.7 (2.6)	19.2 (2.9)	9.7 (0.9)	10.7 (1.4)	8.2 (0.9)	11.8 (2.2)	3.1 (0.6)	2.6 (0.6)	2.0 (0.2)	1.4 (0.2)
Mean Respiratory support (days)	59.4 (14.5)	48.3 (6.7)	54.(4.9)	47.0 (6.9)	43.5 (2.5)	42.2 (3.8)	35 (2.0)	41.2 (4.2)	13.4 (1.1)	12.6 (1.5)	11.1 (0.9)	8.8 (1.2)
Mean Parenteral Nutrition (days)	30.4 (14.4)	17.9 (2.6)	17.9 (2.1)	23.7 (5.2)	17.1 (1.6)	16.1 (1.5)	14.4 (1.0)	18.8 (3.4)	9.1 (0.8)	9.6 (0.9)	8.4 (0.6)	8.4 (1.3)

Data is reported as means or total numbers with standard deviation or proportions in parenthesis, as appropriate. (*p < 0.05).

### Ethics statement

All data was collected as part of routine data collection on neonatal units for which individual parental consent is not sought. The Wales Neonatal Network has permissions in place to access anonymised data from neonatal units. Routinely collected anonymised data of clinical care was acquired by the authors from the Wales Neonatal Network for analysis; no identifiable data was available to the authors. Using the NHS Health Research Authority decision tool (http://www.hra-decisiontools.org.uk/ethics/), this type of research was exempt from specific ethical consent.

### Data cleaning

Data cleaning was undertaken using various strategies as summarised below, with detailed examples in the supplementary information.

1. Repeated values on successive days. If the weight – accurate to 4 significant figures – did not vary over at least two successive days, then the first value on the first day was taken as correct and the subsequent days with the same value were deleted as, from clinical experience, they were very likely to be re-entries without re-weighing in critically unwell and unstable infants.
2. Weights that were very likely to be 1/10th of what they should be were corrected by multiplying by 10 and removing duplicates.
3. Weights that were very likely to be 10 times what they should be were corrected by dividing by 10 and removing duplicates.
4. Weights that were very likely to be too large or small by comparison with the weights either side of them and not correctable as in 2 and 3 above were removed.
5. Patterns of weights that were repeated over successive groups of days were deleted beyond the first.
6. Any infant with only one distinct weight was removed *e.g*. either just birth weight or birth weight repeated for one or more subsequent days. These are mostly infants who died before a second weight could be recorded.

### Statistical analysis

For the first part of the analysis – comparison with UK1990 data – the cohort was divided into three pragmatic gestation bands: 23^+0^ to 25^+6^ weeks, 26^+0^ to 28^+6^ weeks and 29^+0^ to 31^+6^ weeks. Demographic data is reported as means and proportions and was compared (model data *vs* test data) by 2-sided independent samples t-test or Fisher’s test as appropriate. Date-wise weight data from all infants were initially converted to day-wise data till discharge and cross-checked. Infants were first sorted by gender and then by gestation (weeks and days). For calculation of standard deviation scores (SDS) and centiles, weights were first converted from gm to kg. Birth weight (day 0) and weights for each week (day 7, 14, 21 etc.) ±3 days were recorded. SDS was calculated by using LMS Growth Excel add-in (Pan H, Cole TJ, LMS growth, a Microsoft Excel add-in to access growth references based on the LMS method Version 2.76. http://www.healthforallchildren.co.uk/; 2011). The LMS method was used to create NICM growth charts from the UK1990 data^3,4^. Gender-specific mean ± 95% confidence interval of weekly SDS and weights were plotted from birth to discharge.

The complete data was then randomly divided: two-thirds of the data was used for modelling (training cohort) and the remaining third was used as test data to validate the model (test cohort).

Extensive investigation identified an appropriate mixed effects regression model where infant weight was explained by gender, time since birth and time since conception (calculated as the sum of time since birth and gestation minus two weeks) to create predictive weight-gain centiles from birth till discharge (at a variable period of time for different gestations) from the neonatal units. The use of these two distinct timescales is worth highlighting, as one of the main purposes of our work was to distinguish between pre- and post-birth weight gain. The relationship between weight and time since birth was captured using splines with boundary knots at 0 and 14 weeks and other knots at 1, 2, 3 and 4 weeks to properly accommodate the more varied behaviour in the first 4 weeks of life. Time since birth and time since conception were also included as quadratic polynomials. Random effects for each child allowed for correlation between an individual’s observations. Interestingly, no random intercept terms were needed because foetal weight is known to be (effectively) zero when time since conception is zero, and any random intercept at birth is captured by the random slope on the time since conception scale. To reduce the complexity of our modelling, these two slopes were assumed to be uncorrelated. By using parametric models to describe the relationship between weight, time since birth and time since conception, we were able to borrow strengths between different gestation groups and ages.

Predicted quantiles were calculated at 0, 2, 4, 6, 8, 10 and 12 weeks since birth and applied for the following 14 days; thus, the model variance was effectively refreshed every two weeks with the additional data available. Growth trajectories were determined from the model at the following fractiles used in growth charts: 0.004, 0.02, 0.09, 0.25, 0.50, 0.75, 0.91, 0.98 and 0.996^9^. These fractiles can equivalently be interpreted as centiles when (e.g.) 0.02 is multiplied by 100% to give 2%.

All models and figures were produced using R for Windows (R Core Team (2013). R: A language and environment for statistical computing. R Foundation for Statistical Computing, Vienna, Austria. URL http://www.R-project.org/). Each model was tested in three different ways:

1. Plotting model data on new centile curves to check fit.
2. Plotting test data on new centile curves to confirm fit.
3. Plotting test data on UK1990 curves to check fit.

All the test data (all gestations and both genders) was used to numerically compare their fit to both the model we produced and the UK1990 curves by counting the number of weights in each quartile for each. Each weight was compared to quartile levels at the same time to determine in which quartile it belonged. The results for each gender and gestation combination were combined and are given in the tables below where the predicted values are those that should be seen in a completely representative model.

## Results

Between 2011–2014, there were 1606 infants born at <32 weeks of gestation and admitted to a neonatal unit in Wales. Table 2 summarises the demographics for the whole cohort of infants. While the birth SDS were comparable with the reference data (UK1990 or LMS), SDS were significantly lower for all three gestation bands soon after birth until discharge (Table 3), suggesting apparent growth failure during stay (supplementary Fig. 1). 5-weekly SDS data starting from birth is presented in Table 3; full weekly data is presented in Supplementary Tables 1–2.

**Table 3:** Gestation-band specific weekly mean standard deviation score (SDS) of weight with 95% confidence interval (CI) of the mean, from birth up to the 20^th^ week of life.

Completed Week	Birth	5	10	15	19
Gestation-Bands					
**Boys**					
23–25: n	89	51	49	33	5
Mean SDS	−0.043	−1.167	−1.294	−1.472	−1.780
Lower 95% CI	−0.252	−1.350	−1.528	−1.836	−4.006
Upper 95% CI	0.165	−0.984	−1.061	−1.108	0.445
26–28: n	220	192	106	32	11
Mean SDS	−0.280	−1.017	−1.362	−2.319	−2.485
Lower 95% CI	−0.421	−1.139	−1.579	−2.892	−3.440
Upper 95% CI	−0.138	−0.894	−1.145	−1.746	−1.531
29–31: n	482	303	39	15	6
Mean SDS	0.056	−1.133	−1.682	−2.068	−2.059
Lower 95% CI	−0.043	−1.231	−2.100	−2.795	−2.957
Upper 95% CI	0.154	−1.036	−1.264	−1.342	−1.161
**Girls**					
23–25: n	82	53	53	28	8
Mean SDS	0.078	−1.133	−1.338	−1.687	−1.564
Lower 95% CI	−0.104	−1.298	−1.535	−2.049	−2.582
Upper 95% CI	0.260	−0.968	−1.141	−1.325	−0.546
26–28: n	194	166	100	25	4
Mean SDS	−0.006	−1.035	−1.45	−1.456	−1.68
Lower 95% CI	−0.145	−1.163	−1.644	−1.943	−2.419
Upper 95% CI	0.132	−0.907	−1.256	−0.969	−0.941
29–31: n	408	252	23	10	
Mean SDS	0.110	−1.053	−1.593	−1.332	
Lower 95% CI	0.012	−1.160	−2.286	−2.225	
Upper 95% CI	0.207	−0.946	−0.900	−0.439	

SDS was calculated by comparing with the UK1990 birth centiles data at each gestation. Numbers of infants in this table are different from the whole group as presented in Table 2 due to missing data at or within 3 days of the time-points considered, including at birth.

Data from 1067 infants were used as the training cohort for the model, while data from the remaining third of the infants (539 infants) were used to test the model. Table 1 gives details of the gender- and gestation-specific demographics of the two groups of infants used as modelling data and test data. All clinical characteristics in the three gestation groups were well balanced between the training and test cohorts for both boys and girls, except a small but statistically significant difference in the mean gestation of males compared to females in the 29-31-week group, and a significantly higher discharge weight in females on the 29-31-week group in the test cohort.

**Figure 1:**
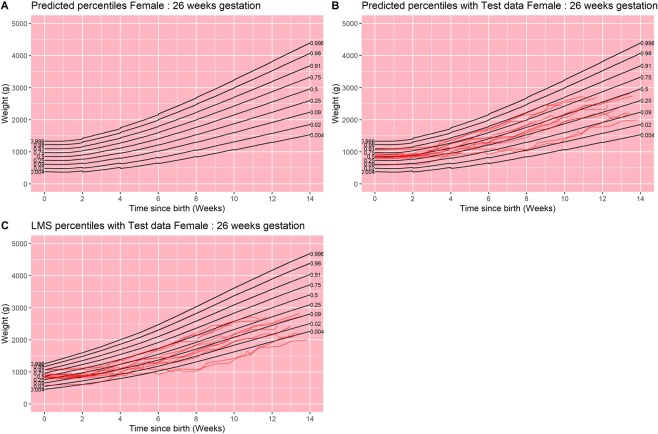
Results from final model for females born at 26 weeks of gestation. (**A**) Model centile curves, (**B**) test data plotted on model centiles, and (**C**) test data plotted on UK1990 reference centiles. Days since birth are plotted on the x-axis on each figure, and weight in grams is plotted on the y-axis. Each point plotted in parts B and C are individual data points from test infants. All infants were included in this analysis.

Using methods described above, and given a hypothetical observation lying on one of the fractiles used in growth charts (i.e. 0.004, 0.02, 0.09, 0.25, 0.50, 0.75, 0.91, 0.98 and 0.996) at a particular point in time (one of 0, 2, 4, 6, 8, 10 and 12 weeks since birth), we were able to calculate the expected values of the two random effects and determine a predicted two-week growth trajectory. Figure 1 represents an example of the model output for girls born at 26 weeks of gestation, with test data plotted on the model centiles and also on the UK1990 curves for comparison. Effectively, the predictive model was recalculated every 2-weeks, leading to small changes in the centile lines at the extremes of the range. However, the fit for the test data was good on the model centiles and was uniformly plotted on lower centiles for the UK1990 data as expected (Table 4). Detailed figures for all gestations are presented in the supplementary files (supplementary information 2 for boys and girls born at 23–31 weeks of gestation) and show good fit of the test data on the model centiles compared to the UK1990 data. From our model, it is possible to create predictive weight centiles for any infant born at any gestation, a fraction of any gestation, or a gestation band, between 23–31 weeks (supplementary information 2).

**Table 4:** Proportions of plotted points in each quartile for the UK1990 data and for our new model including all infants.

	<25%	25–50%	50–75%	>75%
Predicted	1553 (25%)	1553 (25%)	1553 (25%)	1553 (25%)
UK1990	4587 (73.8%)	787 (12.7%)	486 (7.8%)	353 (5.7%)
New model including all infants	1675 (26.9%)	1502 (24.2%)	1571 (25.3%)	1465 (23.6%)

The predicted values represent what should be expected in a completely representative model.

**Figure 2:**
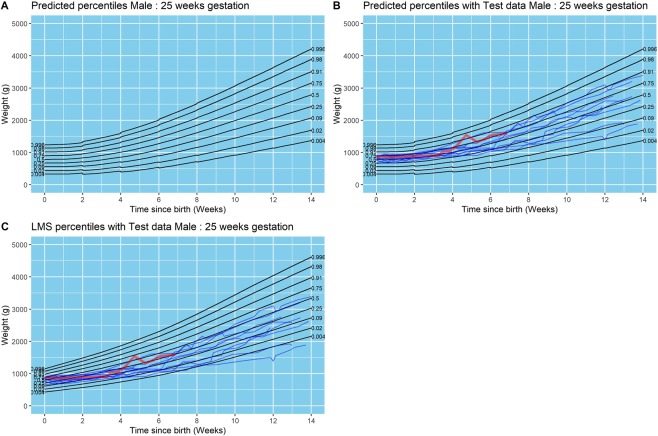
Results from final model for males born at 25 weeks of gestation. (**A**) Model centile curves, (**B**) test data plotted on model centiles, and (**C**) test data plotted on UK1990 reference centiles. Days since birth are plotted on the x-axis on each figure, and weight in grams is plotted on the y-axis. Each point plotted in parts B and C are individual data points from test infants. All infants were included in this analysis. Infants who died are represented as a bold red line in the figure.

**Figure 3:**
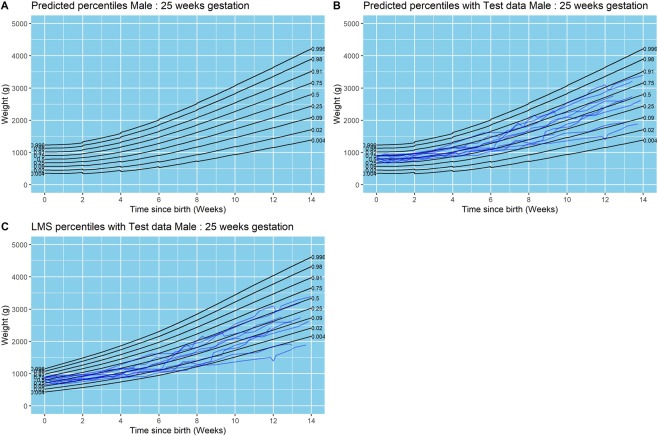
Results from final model for males born at 25 weeks of gestation. (**A**) Model centile curves, (**B**) test data plotted on model centiles, and (**C**) test data plotted on UK1990 reference centiles. Days since birth are plotted on the x-axis on each figure, and weight in grams is plotted on the y-axis. Each point plotted in parts B and C are individual data points from test infants. Only surviving infants were included in this analysis.

In our cohort, there were 77 deaths in total before discharge from the neonatal unit (Table 1). As infants who died could have affected the model, a sub-group analysis for the growth curves was undertaken excluding all of the infants who died. Representative data for male infants born at 25 weeks of gestation with and without infants who died is presented in Figs. 2 and 3 respectively. Model fit for the subgroup analysis is presented in Table 5. Proportions of plots in each quartile were almost identical for both models (all infants and surviving infants).

**Table 5:** Proportions of plotted points in each quartile for the UK1990 data and for our new model including surviving infants only.

	<25%	25–50%	50–75%	>75%
Predicted	1538.5 (25%)	1538.5 (25%)	1538.5 (25%)	1538.5 (25%)
UK1990	3982 (64.7%)	1225 (19.9%)	632 (10.3%)	315 (5.1%)
New model including surviving infants	1663 (27.0%)	1492 (24.2%)	1551 (25.2%)	1448 (23.5%)

The predicted values represent what should be expected in a completely representative model.

## Discussion

Although it has been shown that cross-sectional birthweight centiles differ in shape from postnatal growth curves in infants <32 weeks gestation and how the mean growth curves vary by gestation^10^, this is the first attempt to analyse longitudinal weight data from extreme preterm infants and appropriately model them to produce predictive weight centile references until discharge. Our results show good fit for preterm infants at all gestations tested, while we also demonstrate poor fit of the data on the UK1990 curves.

Our proposed model has several features that distinguish it from the LMS approach, which is currently used to construct Newborn and Infant Close Monitoring (NICM) Growth Charts^11^. Most importantly, it is based around two different timescales: one measuring elapsed time since conception, and the other elapsed time since birth. It seems to us that both these timescales are of immediate and obvious relevance in assessing the growth of preterm infants. A convenient aspect of the two-timescale approach is that all random effects may be thought of as growth rates (slopes), the intercept terms being (in the case of time since conception) known to be zero, and (in the case of time since birth) determined by growth since conception.

The discontinuities in Fig. 1 (and other similar figures in supplementary information) are intentional. In essence, such figures are composed of numerous individual two-week longitudinal predictions. While our underlying model can produce predictions of arbitrary duration and based on arbitrarily timed (since conception, and since birth) observed percentiles, such estimates would only really be tractable and practicable in dynamic, digital media. For static, printed formats, Fig. 1 represents a visual compromise: predictions may be based on observed percentiles at most one week distant from the truth and will then last between one and two weeks. The discontinuities also serve as a visual reminder of the difference between cross-sectional and longitudinal percentiles. For example, even among infants observed at the 90th percentile, there will be variation in subsequent growth; we plot the mean predicted trajectory, but the steps in the curves at the end of each two-week segment reflect real variation around this central estimate.

Preterm infants are probably the only population group where “growth” assessment is still undertaken by using cross-sectional reference charts plotting centiles at birth (or *in-utero* growth) as opposed to longitudinal measurements^12^. This was based on the principle that postnatal weight gain should reflect *in-utero* weight gain^13^. However, this standard seems to have never been achieved in practice^6,10,14^. It is also evident in almost all growth data presented in this high-risk population where preterm infants suffer a period of initial growth failure (defined variably) when plotted on the cross-sectional growth charts^6,10,15,16^. Aggressive nutritional interventions to achieve growth rates similar to the intrauterine growth trajectories have had variable success rates^17^ but more importantly serious long-term concerns have emerged^18^. Thus, the practical utility of traditional growth charts based on cross sectional growth data of in-utero fetuses to monitor growth after preterm birth is questionable. In a recent detailed review, Villar and colleagues^19^ elegantly argued against this principle, as preterm infants are often unable to achieve the recommendations from these charts, even after receiving the best possible care^17^, or with advanced neonatal care^10,20^. In addition, studies have shown that preterm infants often have preserved head growth in preference to body weight during postnatal life, accounting for apparent “growth failure”^21^. More importantly, the authors argued that attempting to reflect *in-utero* weight gain by accelerated postnatal growth may not be desirable due to long-term metabolic adverse effects^18,22,23^.

In a recent systematic review of longitudinal studies attempting to create postnatal growth charts for preterm infants, Villar and colleagues^8^ noted that overall methodologic quality of the studies was fair to low by a scoring system. Several studies included in the review were from historical cohorts of infants, and their management would not be comparable with current practice. However, there were nine studies that looked at cohorts of infants born after the year 2000^10,24–31^, which are more representative of current clinical care. Only three of these studies had sufficient sample size for analysis^10,24,27^. Of the nine studies, six studies did not publish any centile lines^10,24,26,28–30^ and one study produced only birth-weight specific data with no attempt at constructing charts^25^. The study by Bocca-Tjeertes and colleagues^27^ published reference centiles for growth of preterm infants up to 15 months of age. However, the early postnatal period from birth up to discharge from the neonatal unit was presented in a compressed format in the chart and would be challenging to use clinically on the neonatal unit. The study by Villar and colleagues^31^, while methodologically rigorous, had a small sample size (201 infants), with only 28 infants born below 33 weeks of gestation (centile charts were presented from 27 weeks of gestation). Thus, no longitudinal reference centile charts exist to monitor the most vulnerable preterm infants born at extremes of gestation, who are at the highest risk of having growth problems. This is a specific gap we have aimed to fill.

Our study has several strengths including a large sample size and using gestation (and not birth weight) as the correct representation of prematurity. We have used rigorous and detailed analysis followed by easily replicable mixed modelling methods allowing for variability (a constant in this population) at every stage, resulting in predictive weight-gain centiles of extreme preterm infants. This contemporary data reflects current clinical practice and any variations in clinical management are expected to be reflected in the large data set analysed. Borrowing strengths across gestation groups has allowed us to make meaningful predictions for even the early gestation groups, where smaller numbers of infants were available to provide data. Parametric modelling allowed borrowing of strength across the whole dataset, so that (in principle) even a full-term infant could contribute something to our estimation of growth curves for extremely preterm infants although in practice, such contributions are small. Although we have presented reference centiles for only a few chosen gestations, this is neither due to data paucity nor model inadequacy but is instead a limitation of static graphical presentation. The model is in principle infinitely fine-grained and interpolates seamlessly between different ages and gestations. Any choice of static plots will necessarily represent a compromise between precision and practicality: it would be impractical to suggest neonatal units employ books of growth charts hundreds of pages long corresponding to all possible combinations of age and gestation. Nevertheless, we consider dynamic plots an attractive and viable option in the near future, where growth percentiles tailored exactly to a child’s age and gestation are instantly available electronically. Our data and model can already produce such dynamic predictions; our static representations provide an example of these possibilities.

There are some limitations that need pointing out. Although most infants in our cohort come from a population where gestation is assessed early at around 12–14 weeks by ultrasound, this data was not available to us to cross-check. Weight measurements were taken using similar machines at the different hospitals, but these were not standardised or quality controlled. Morbidity data in our retrospective cohort was of an insufficient quality to include in the model predictions for individualised charts. All of these limitations can be corrected by designing a prospective national study to collect representative data.

In conclusion, we have published the first gestation- and gender-specific predictive longitudinal weight gain charts in extreme preterm infants. This model can be used to produce similar reference charts for other anthropological measurements from preterm infants, by collecting prospective data in a national study.

## Supplementary information

Supplementary Information.
