# Supplementary material for: Modelling predictive gender- and gestation-specific weight reference centiles for preterm infants using a population-based cohort study
Source: Sci Rep. 2020 Mar 4;10:4032. doi: 10.1038/s41598-020-60895-6 (PMC7055271; doi:10.1038/s41598-020-60895-6)
Supplement: Supplementary file 1 — Supplementary Information. [file 41598_2020_60895_MOESM1_ESM.pdf]

# Modelling predictive gender- and gestation-specific weight reference centiles for preterm infants using a population-based cohort study

W. John Watkins, Daniel Farewell, Sujoy Banerjee, Hesham Nasef, Anitha James, Mallinath Chakraborty

**Supplementary Information File**

**Data Cleaning: Examples**

Cleaning of original data was undertaken using several different strategies, as detailed below with examples.

1. Repeated values on successive days. The infants may have received attention but not have been weighed every day in which case we believe it was fairly common practice to simply re-enter the weight from the previous actual measurement as a "working" weight. It was thought to be highly unlikely that actual weight measured on a scale would be identical for several consecutive days. Consequently, if the weight didn't vary over at least two successive days, then the first value on the first day was taken as correct and the subsequent days with the same value were deleted. In the example below a number of weights are repeated and the repeats were removed by the cleaning process.

| ID | Gender | Gestation | Age (Days) | Weight (g) |
|---|---|---|---|---|
| 82 | 1 | 26 | 100 | 2920 |
| 82 | 1 | 26 | 101 | 3000 |
| 82 | 1 | 26 | 102 | 3000 |
| 82 | 1 | 26 | 103 | 3000 |
| 82 | 1 | 26 | 104 | 3015 |
| 82 | 1 | 26 | 105 | 3015 |
| 82 | 1 | 26 | 106 | 3015 |
| 82 | 1 | 26 | 107 | 3015 |
| 82 | 1 | 26 | 109 | 3145 |
| 82 | 1 | 26 | 110 | 3145 |
| 82 | 1 | 26 | 111 | 3375 |
| 82 | 1 | 26 | 112 | 3375 |
| 82 | 1 | 26 | 113 | 3375 |
| 82 | 1 | 26 | 114 | 3375 |
| 82 | 1 | 26 | 115 | 3375 |
| 82 | 1 | 26 | 116 | 3375 |
| 82 | 1 | 26 | 117 | 3375 |
| 82 | 1 | 26 | 120 | 3290 |

| ID | Gender | Gestation | Age (Days) | Weight (g) |
|---|---|---|---|---|
| 82 | 1 | 26 | 100 | 2920 |
| 82 | 1 | 26 | 101 | 3000 |
| 82 | 1 | 26 | 104 | 3015 |
| 82 | 1 | 26 | 109 | 3145 |
| 82 | 1 | 26 | 111 | 3375 |
| 82 | 1 | 26 | 120 | 3290 |

2. Weight which were likely to be 1/10th of what they should be through simple data entry error were corrected by multiplying by 10 and removing duplicates. In the event the actual weight was slightly higher than expected, *e.g.* 220 was in fact 2204, then the difference between the actual and the multiple is relatively small and unlikely to affect any analysis.

| ID | Gender | Gestation | Age (Days) | Weight (g) |
|---|---|---|---|---|
| 83 | 1 | 26 | 77 | 1990 |
| 83 | 1 | 26 | 78 | 220 |
| 83 | 1 | 26 | 79 | 220 |
| 83 | 1 | 26 | 81 | 2200 |
| 83 | 1 | 26 | 83 | 2280 |

| ID | Gender | Gestation | Age (Days) | Weight (g) |
|---|---|---|---|---|
| 83 | 1 | 26 | 77 | 1990 |
| 83 | 1 | 26 | 78 | 2200 |
| 83 | 1 | 26 | 81 | 2200 |
| 83 | 1 | 26 | 83 | 2280 |

3. Weight which were likely to be 10 times what they should be through simple data entry error were corrected by dividing by 10 and removing duplicates.

| ID | Gender | Gestation | Age (Days) | Weight (g) |
|---|---|---|---|---|
| 96 | 1 | 26 | 173 | 4455 |
| 96 | 1 | 26 | 174 | 46150 |
| 96 | 1 | 26 | 175 | 46150 |
| 96 | 1 | 26 | 176 | 4735 |
| 96 | 1 | 26 | 177 | 4735 |

| ID | Gender | Gestation | Age (Days) | Weight (g) |
|---|---|---|---|---|
| 96 | 1 | 26 | 173 | 4455 |
| 96 | 1 | 26 | 174 | 4615 |
| 96 | 1 | 26 | 176 | 4735 |

4. Weights which appeared too large or small by comparison with the weights on either side of them were removed. These weights were identified through coding if they were greater than 20% larger or smaller than the preceding weight. Once identified each suspect weight was individually examined to assess the likelihood of being an error.  If this was the decision then the weight was deleted, as shown in the example below.

| ID | Gender | Gestation | Age (Days) | Weight (g) |
|---|---|---|---|---|
| 623 | 2 | 26 | 27 | 749 |
| 623 | 2 | 26 | 28 | 1230 |
| 623 | 2 | 26 | 31 | 759 |
| 623 | 2 | 26 | 32 | 745 |

→

| ID | Gender | Gestation | Age (Days) | Weight (g) |
|---|---|---|---|---|
| 623 | 2 | 26 | 27 | 749 |
| 623 | 2 | 26 | 31 | 759 |
| 623 | 2 | 26 | 32 | 745 |

However in this second instance, despite a change of over 40% weight this happened over a period of 22 days so was considered plausible and was not removed.

| ID | Gender | Gestation | Age (Days) | Weight (g) |
|---|---|---|---|---|
| 16 | 2 | 24 | 37 | 790 |
| 16 | 2 | 24 | 42 | 1150 |
| 16 | 2 | 24 | 64 | 1680 |
| 16 | 2 | 24 | 66 | 1690 |

←

5. Patterns of weights which were repeated over successive groups of days were deleted beyond the first as the repeat of a succession of very precise numbers was judged to be very unlikely.

| ID | Gender | Gestation | Age (Days) | Weight (g) |
|---|---|---|---|---|
| 571 | 2 | 24 | 96 | 2040 |
| 571 | 2 | 24 | 97 | 2170 |
| 571 | 2 | 24 | 98 | 2040 |
| 571 | 2 | 24 | 99 | 2170 |
| 571 | 2 | 24 | 100 | 2170 |

→

| ID | Gender | Gestation | Age (Days) | Weight (g) |
|---|---|---|---|---|
| 571 | 2 | 24 | 96 | 2040 |
| 571 | 2 | 24 | 97 | 2170 |
| 571 | 2 | 24 | 100 | 2170 |

6. Any infant with only one distinct weight was removed, *e.g.* either just birth weight or birth weight repeated for one or more subsequent days as below. This decision was made as these measurement(s) would provide no longitudinal input into the analysis.

| ID | Gender | Gestation | Age (Days) | Weight (g) |
|---|---|---|---|---|
| 197 | 1 | 28 | 84 | 3530 |
| 198 | 1 | 28 | 0 | 1500 |
| 198 | 1 | 28 | 1 | 1500 |
| 198 | 1 | 28 | 2 | 1500 |
| 198 | 1 | 28 | 3 | 1500 |
| 198 | 1 | 28 | 4 | 1500 |
| 198 | 1 | 28 | 5 | 1500 |
| 199 | 1 | 28 | 0 | 1055 |

| ID | Gender | Gestation | Age (Days) | Weight (g) |
|---|---|---|---|---|
| 197 | 1 | 28 | 84 | 3530 |
| 199 | 1 | 28 | 0 | 1055 |

**Standard Deviation Scores (SDS)**

As described in the methods and results, SDS was calculated for the whole cohorts using weekly weights, as represented in supplementary figure 1.

A: 23-25 weeks

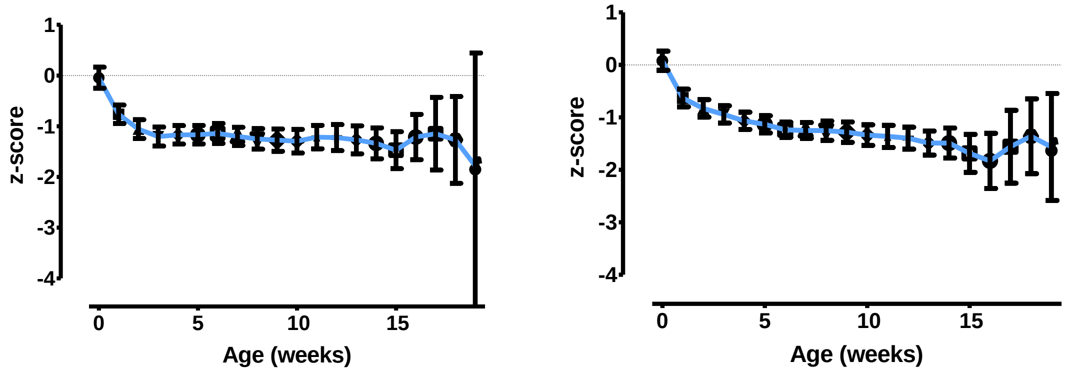


B: 26-28 weeks

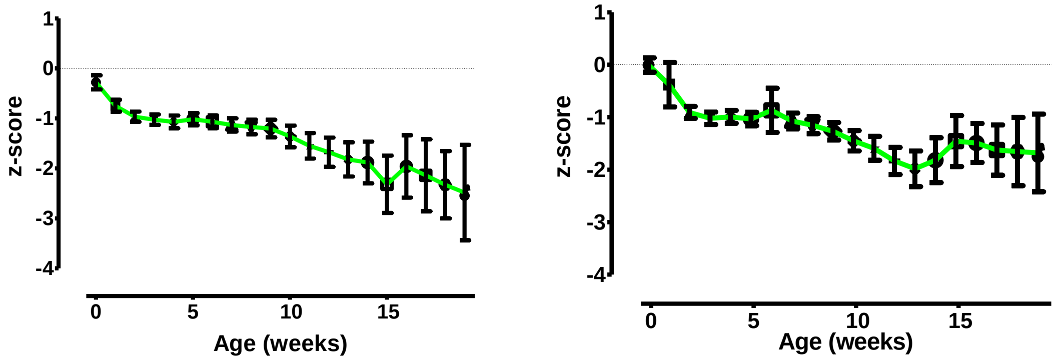


C: 29-31 weeks

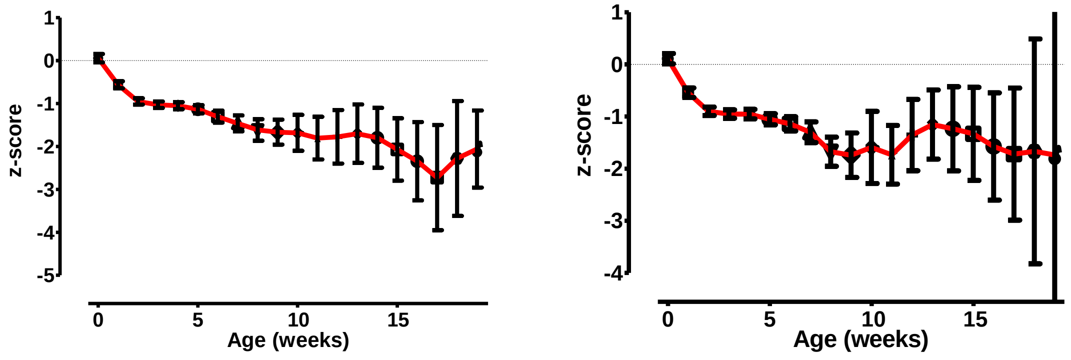


Supplementary Figure 1: Weekly SDS (z-score) of males (left panel) and females (right panel) in the three gestation bands (A) 23-25 weeks (blue line), (B) 26-28 weeks (green line), and (C) 29-31 weeks (red line). Age in weeks is represented on the x-axis while SDS (z-score) is represented on the y-axis. Each data point is the mean weekly SDS ±95% confidence intervals (CI).

| Completed Week<br>Gestation-Bands | Birth | 1 | 2 | 3 | 4 | 5 | 6 | 7 | 8 | 9 | 10 | 11 | 12 | 13 | 14 | 15 | 16 | 17 | 18 | 19 |
|---|---|---|---|---|---|---|---|---|---|---|---|---|---|---|---|---|---|---|---|---|
| | | | | | | | | | | | | | | | | | | | | |
| 23-25: n | 89 | 69 | 58 | 56 | 53 | 51 | 51 | 51 | 49 | 48 | 49 | 47 | 45 | 44 | 40 | 33 | 28 | 16 | 12 | 5 |
| Mean SDS | -0.043 | -0.763 | -1.056 | -1.203 | -1.169 | -1.167 | -1.142 | -1.199 | -1.250 | -1.273 | -1.294 | -1.214 | -1.224 | -1.268 | -1.337 | -1.472 | -1.212 | -1.147 | -1.269 | -1.780 |
| Lower 95% CI | -0.252 | -0.946 | -1.241 | -1.391 | -1.351 | -1.350 | -1.339 | -1.378 | -1.453 | -1.493 | -1.528 | -1.445 | -1.481 | -1.544 | -1.643 | -1.836 | -1.659 | -1.864 | -2.125 | -4.006 |
| Upper 95% CI | 0.165 | -0.581 | -0.870 | -1.015 | -0.987 | -0.984 | -0.945 | -1.021 | -1.047 | -1.054 | -1.061 | -0.983 | -0.967 | -0.991 | -1.032 | -1.108 | -0.764 | -0.430 | -0.413 | 0.445 |
| | | | | | | | | | | | | | | | | | | | | |
| 26-28: n | 220 | 206 | 200 | 194 | 195 | 192 | 191 | 181 | 160 | 132 | 106 | 89 | 73 | 58 | 42 | 32 | 20 | 17 | 15 | 11 |
| Mean SDS | -0.280 | -0.748 | -0.967 | -1.027 | -1.071 | -1.017 | -1.069 | -1.133 | -1.172 | -1.204 | -1.362 | -1.549 | -1.677 | -1.821 | -1.882 | -2.319 | -1.960 | -2.140 | -2.327 | -2.485 |
| Lower 95% CI | -0.421 | -0.866 | -1.069 | -1.132 | -1.198 | -1.139 | -1.196 | -1.265 | -1.318 | -1.380 | -1.579 | -1.804 | -1.970 | -2.163 | -2.299 | -2.892 | -2.584 | -2.860 | -3.000 | -3.440 |
| Upper 95% CI | -0.138 | -0.630 | -0.865 | -0.921 | -0.944 | -0.894 | -0.941 | -1.000 | -1.026 | -1.029 | -1.145 | -1.294 | -1.384 | -1.478 | -1.465 | -1.746 | -1.337 | -1.420 | -1.654 | -1.531 |
| | | | | | | | | | | | | | | | | | | | | |
| 29-31: n | 482 | 465 | 455 | 427 | 397 | 303 | 198 | 130 | 92 | 65 | 39 | 27 | 20 | 19 | 17 | 15 | 13 | 8 | 6 | 6 |
| Mean SDS | 0.056 | -0.563 | -0.952 | -1.028 | -1.049 | -1.133 | -1.305 | -1.462 | -1.615 | -1.666 | -1.682 | -1.806 | -1.776 | -1.701 | -1.798 | -2.068 | -2.342 | -2.726 | -2.279 | -2.059 |
| Lower 95% CI | -0.043 | -0.646 | -1.025 | -1.103 | -1.132 | -1.231 | -1.444 | -1.646 | -1.866 | -1.959 | -2.100 | -2.305 | -2.400 | -2.383 | -2.495 | -2.795 | -3.255 | -3.951 | -3.618 | -2.957 |
| Upper 95% CI | 0.154 | -0.480 | -0.879 | -0.953 | -0.967 | -1.036 | -1.166 | -1.277 | -1.364 | -1.374 | -1.264 | -1.307 | -1.152 | -1.020 | -1.101 | -1.342 | -1.429 | -1.501 | -0.9398 | -1.161 |

Supplementary table 1: Table 2: Gestation-band specific weekly mean standard deviation score (SDS) of weight for boys, with 95% confidence interval (CI) of the mean, from birth up to the 20th week of life. SDS was calculated by comparing with the UK1990 birth centiles data at each gestation.

| Completed Week<br>Gestation-Bands | Birth | 1 | 2 | 3 | 4 | 5 | 6 | 7 | 8 | 9 | 10 | 11 | 12 | 13 | 14 | 15 | 16 | 17 | 18 | 19 |
|---|---|---|---|---|---|---|---|---|---|---|---|---|---|---|---|---|---|---|---|---|
| | | | | | | | | | | | | | | | | | | | | |
| 23-25: n | 82 | 66 | 59 | 56 | 56 | 53 | 53 | 53 | 53 | 53 | 53 | 52 | 48 | 46 | 37 | 28 | 20 | 14 | 12 | 8 |
| Mean SDS | 0.078 | -0.632 | -0.829 | -0.944 | -1.066 | -1.133 | -1.236 | -1.25 | -1.254 | -1.282 | -1.338 | -1.363 | -1.397 | -1.491 | -1.49 | -1.687 | -1.83 | -1.56 | -1.36 | -1.564 |
| Lower 95% CI | -0.104 | -0.799 | -0.996 | -1.110 | -1.229 | -1.298 | -1.380 | -1.401 | -1.438 | -1.477 | -1.535 | -1.574 | -1.606 | -1.720 | -1.777 | -2.049 | -2.355 | -2.254 | -2.074 | -2.582 |
| Upper 95% CI | 0.260 | -0.465 | -0.662 | -0.780 | -0.902 | -0.968 | -1.092 | -1.099 | -1.069 | -1.087 | -1.141 | -1.152 | -1.188 | -1.262 | -1.203 | -1.325 | -1.305 | -0.866 | -0.646 | -0.546 |
| | | | | | | | | | | | | | | | | | | | | |
| 26-28: n | 194 | 170 | 172 | 169 | 166 | 166 | 162 | 157 | 142 | 123 | 100 | 79 | 59 | 42 | 32 | 25 | 15 | 9 | 5 | 4 |
| Mean SDS | -0.006 | -0.379 | -0.910 | -1.019 | -0.995 | -1.035 | -0.869 | -1.072 | -1.153 | -1.268 | -1.45 | -1.593 | -1.834 | -1.983 | -1.819 | -1.456 | -1.491 | -1.626 | -1.655 | -1.68 |
| Lower 95% CI | -0.145 | -0.802 | -1.026 | -1.139 | -1.118 | -1.163 | -1.291 | -1.223 | -1.315 | -1.436 | -1.644 | -1.821 | -2.092 | -2.323 | -2.247 | -1.943 | -1.863 | -2.106 | -2.305 | -2.419 |
| Upper 95% CI | 0.132 | 0.044 | -0.795 | -0.899 | -0.872 | -0.907 | -0.449 | -0.921 | -0.991 | -1.100 | -1.256 | -1.365 | -1.576 | -1.643 | -1.391 | -0.969 | -1.119 | -1.146 | -1.005 | -0.941 |
| | | | | | | | | | | | | | | | | | | | | |
| 29-31: n | 408 | 391 | 384 | 363 | 329 | 252 | 178 | 107 | 65 | 37 | 23 | 20 | 15 | 13 | 10 | 10 | | | | |
| Mean SDS | 0.110 | -0.544 | -0.900 | -0.953 | -0.954 | -1.053 | -1.142 | -1.301 | -1.675 | -1.741 | -1.593 | -1.734 | -1.356 | -1.154 | -1.235 | -1.332 | | | | |
| Lower 95% CI | 0.012 | -0.634 | -0.982 | -1.033 | -1.044 | -1.160 | -1.279 | -1.501 | -1.955 | -2.167 | -2.286 | -2.297 | -2.040 | -1.817 | -2.043 | -2.225 | | | | |
| Upper 95% CI | 0.207 | -0.453 | -0.819 | -0.872 | -0.863 | -0.946 | -1.005 | -1.101 | -1.395 | -1.315 | -0.900 | -1.171 | -0.672 | -0.491 | -0.427 | -0.439 | | | | |

Supplementary Table 2: Gestation-band specific weekly mean standard deviation score (SDS) of weight for girls, with 95% confidence interval (CI) of the mean, from birth up to the 20th week of life. SDS was calculated by comparing with the UK1990 birth centiles data at each gestation.

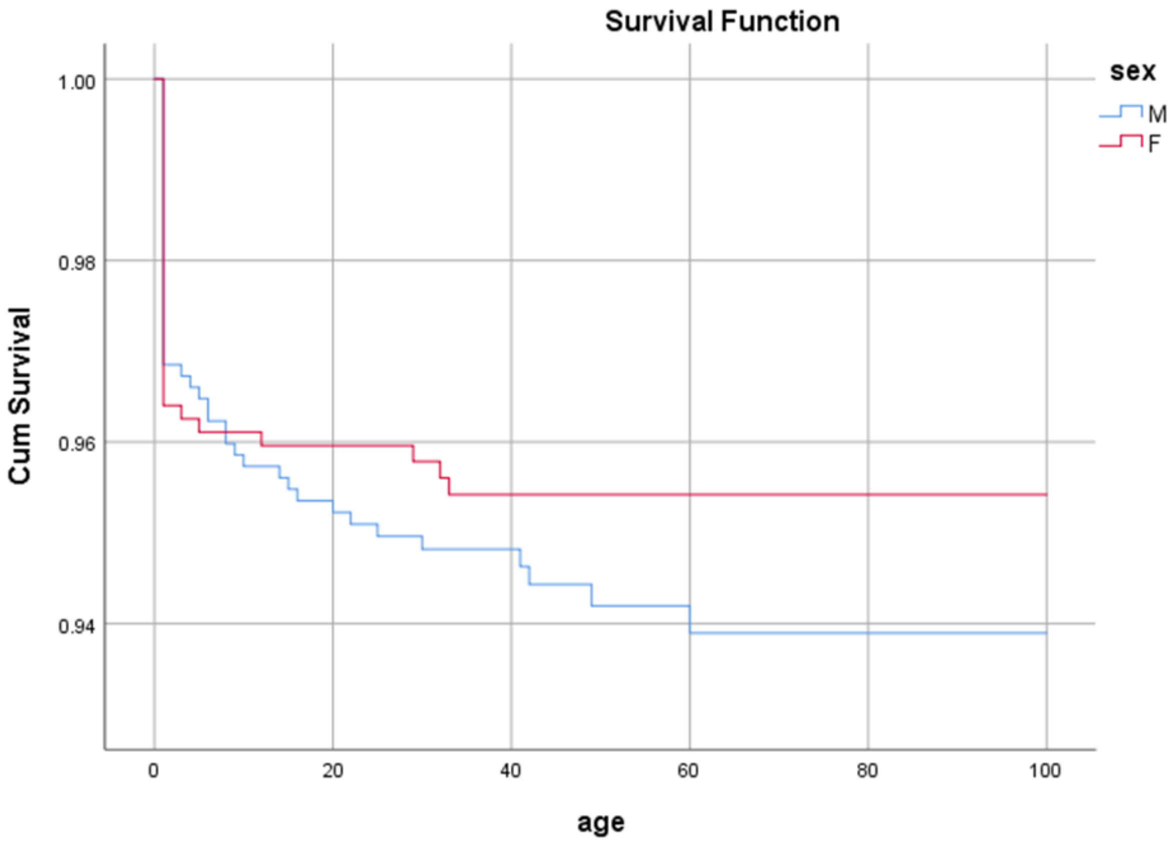


Supplementary Figure 2: Time of death of preterm infants after live birth (blue line representing males and pink line for females). Age in days is represented on the x-axis with cumulative proportion surviving at each stage is represented on the y-axis.

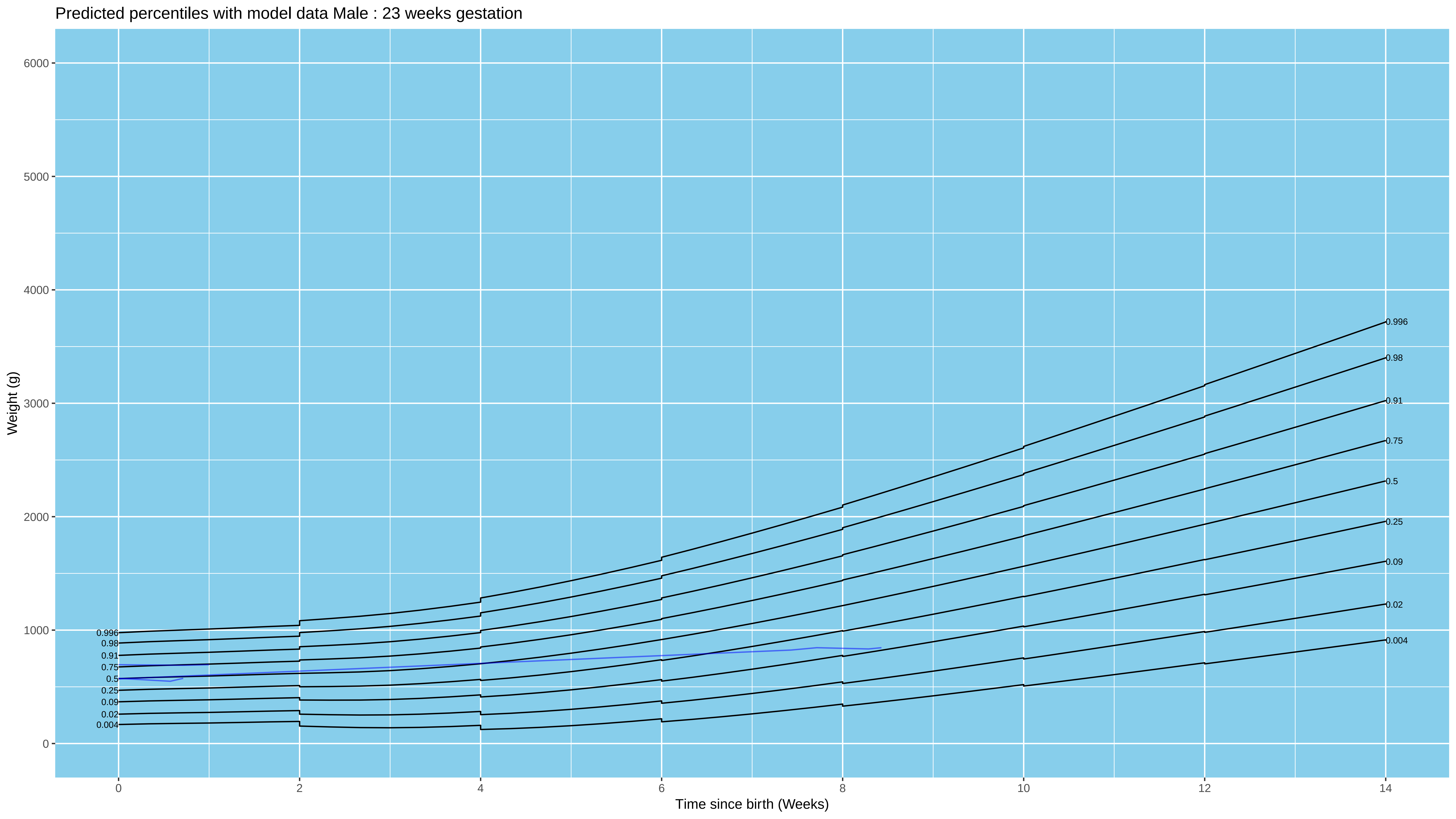
Predicted percentiles with model data Male : 23 weeks gestation
Weight (g)
Time since birth (Weeks)
0
1000
2000
3000
4000
5000
6000
0
2
4
6
8
10
12
14
0.996
0.98
0.91
0.75
0.5
0.25
0.09
0.02
0.004

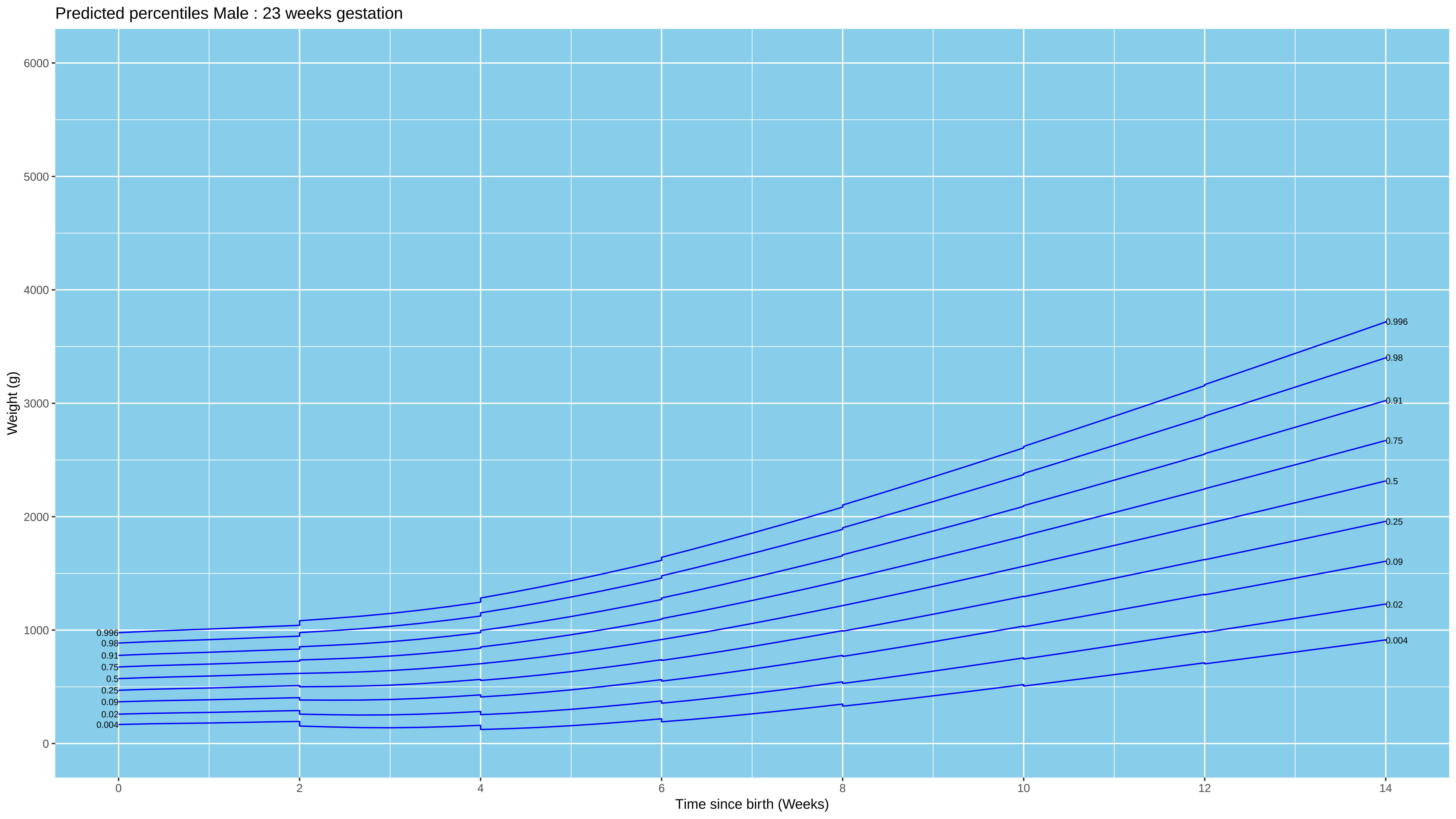

Predicted percentiles Male : 23 weeks gestation
Weight (g)
Time since birth (Weeks)
0
1000
2000
3000
4000
5000
6000
0
2
4
6
8
10
12
14
0.996
0.98
0.91
0.75
0.5
0.25
0.09
0.02
0.004

Predicted percentiles with Test data Male : 23 weeks gestation

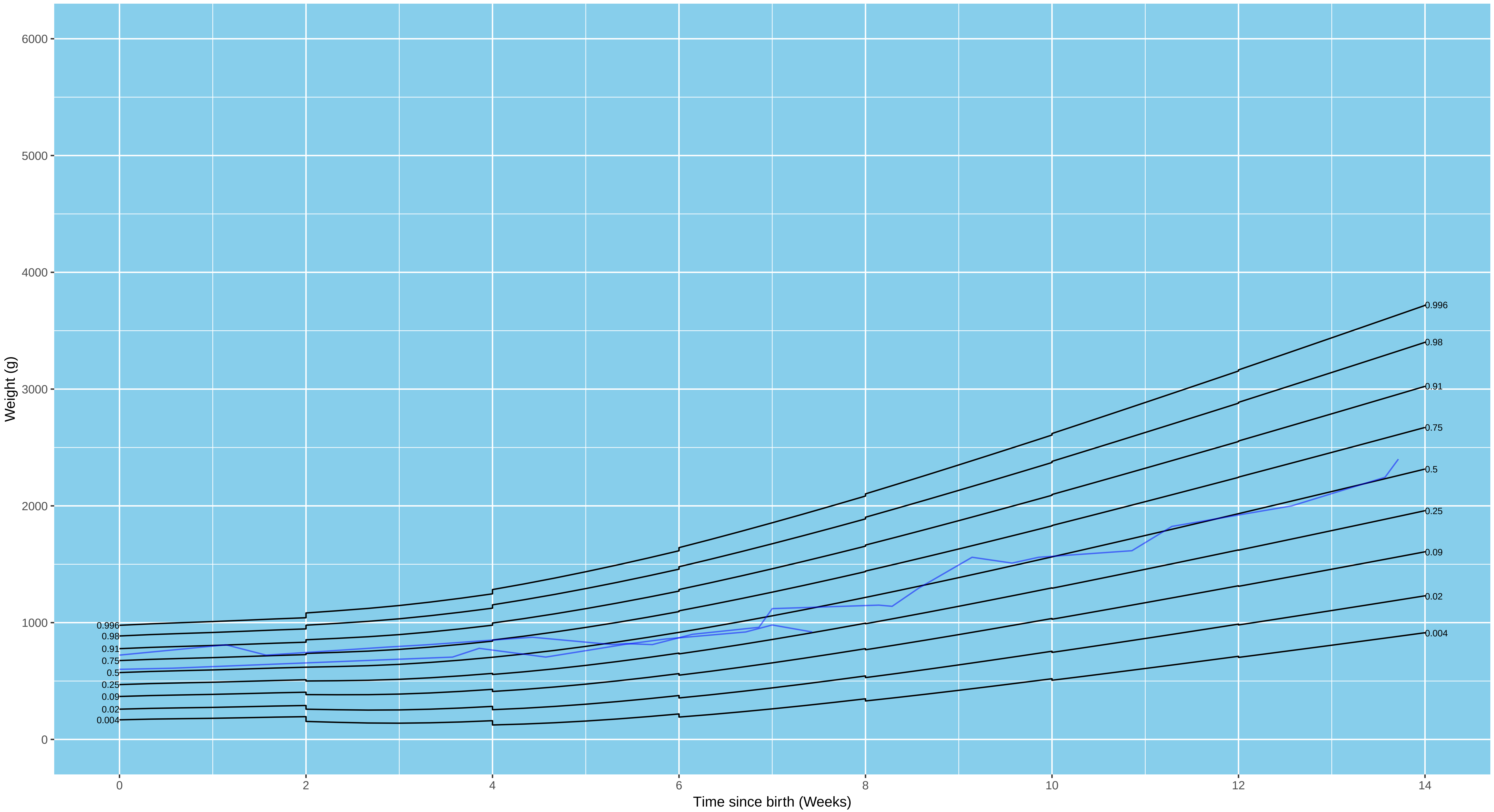

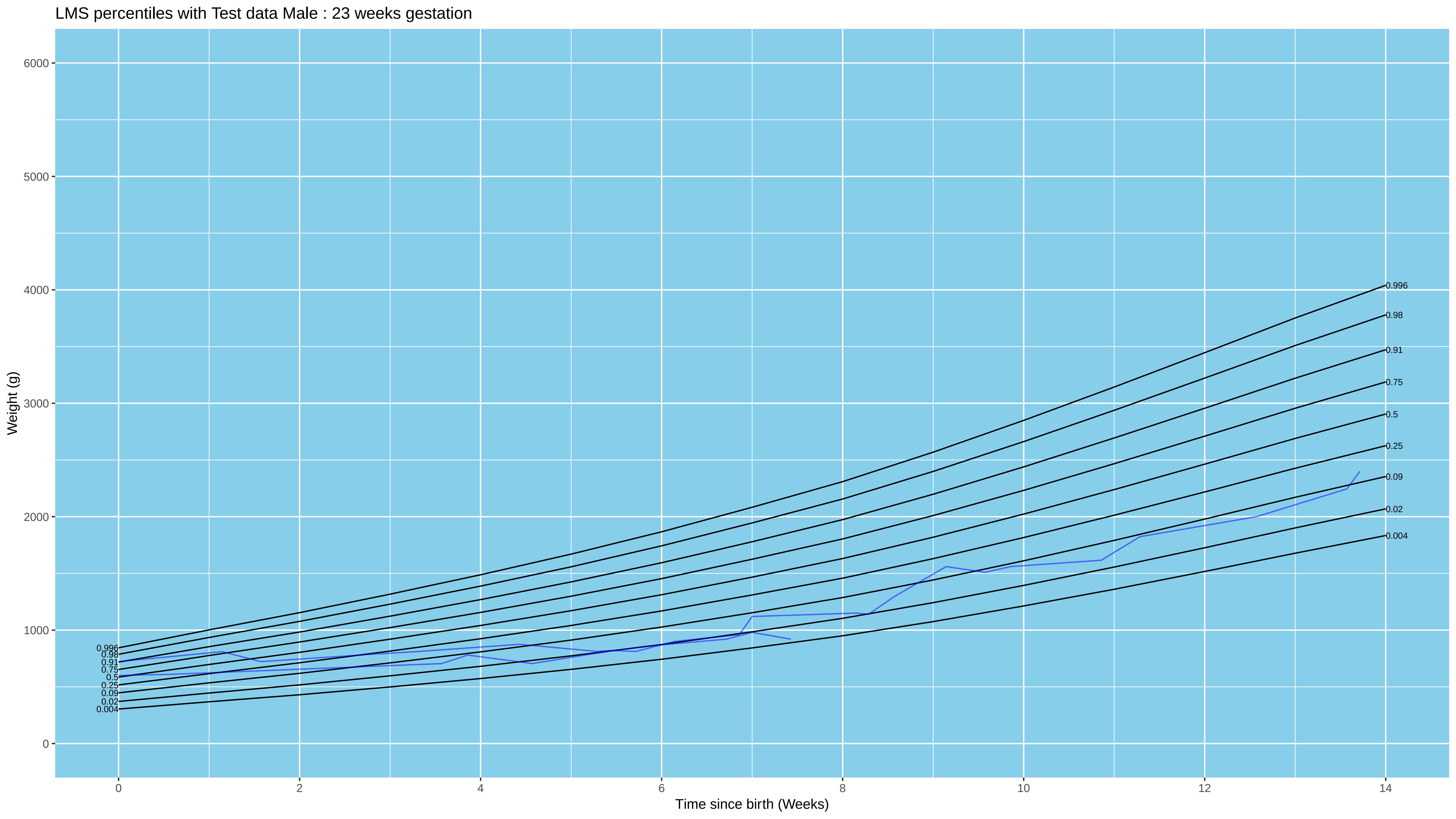

LMS percentiles with Test data Male : 23 weeks gestation
Weight (g)
Time since birth (Weeks)
0
1000
2000
3000
4000
5000
6000
0
2
4
6
8
10
12
14
0.996
0.98
0.91
0.75
0.5
0.25
0.09
0.02
0.004

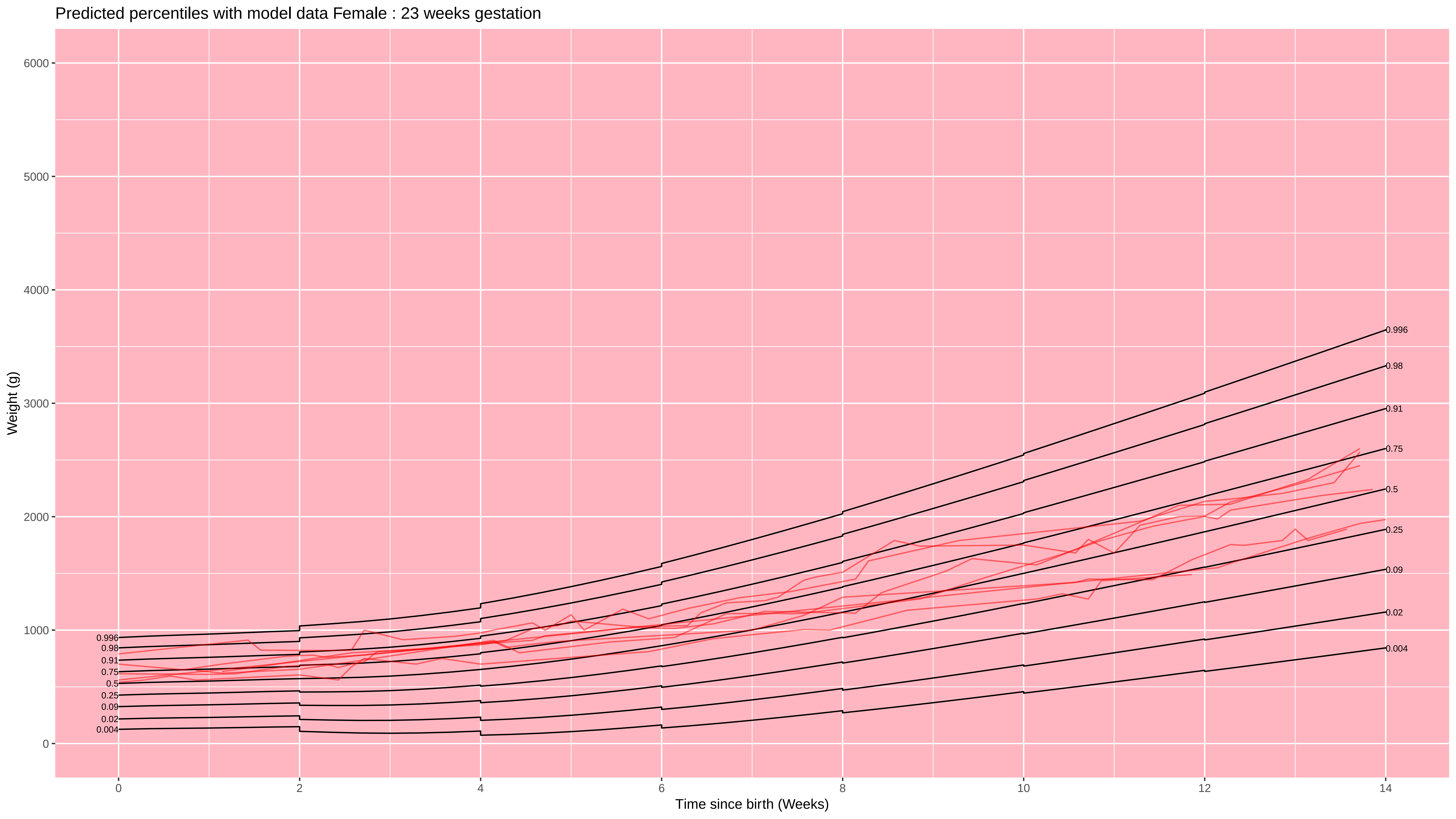

Predicted percentiles with model data Female : 23 weeks gestation
Weight (g)
Time since birth (Weeks)
0
1000
2000
3000
4000
5000
6000
0
2
4
6
8
10
12
14
0.996
0.98
0.91
0.75
0.5
0.25
0.09
0.02
0.004

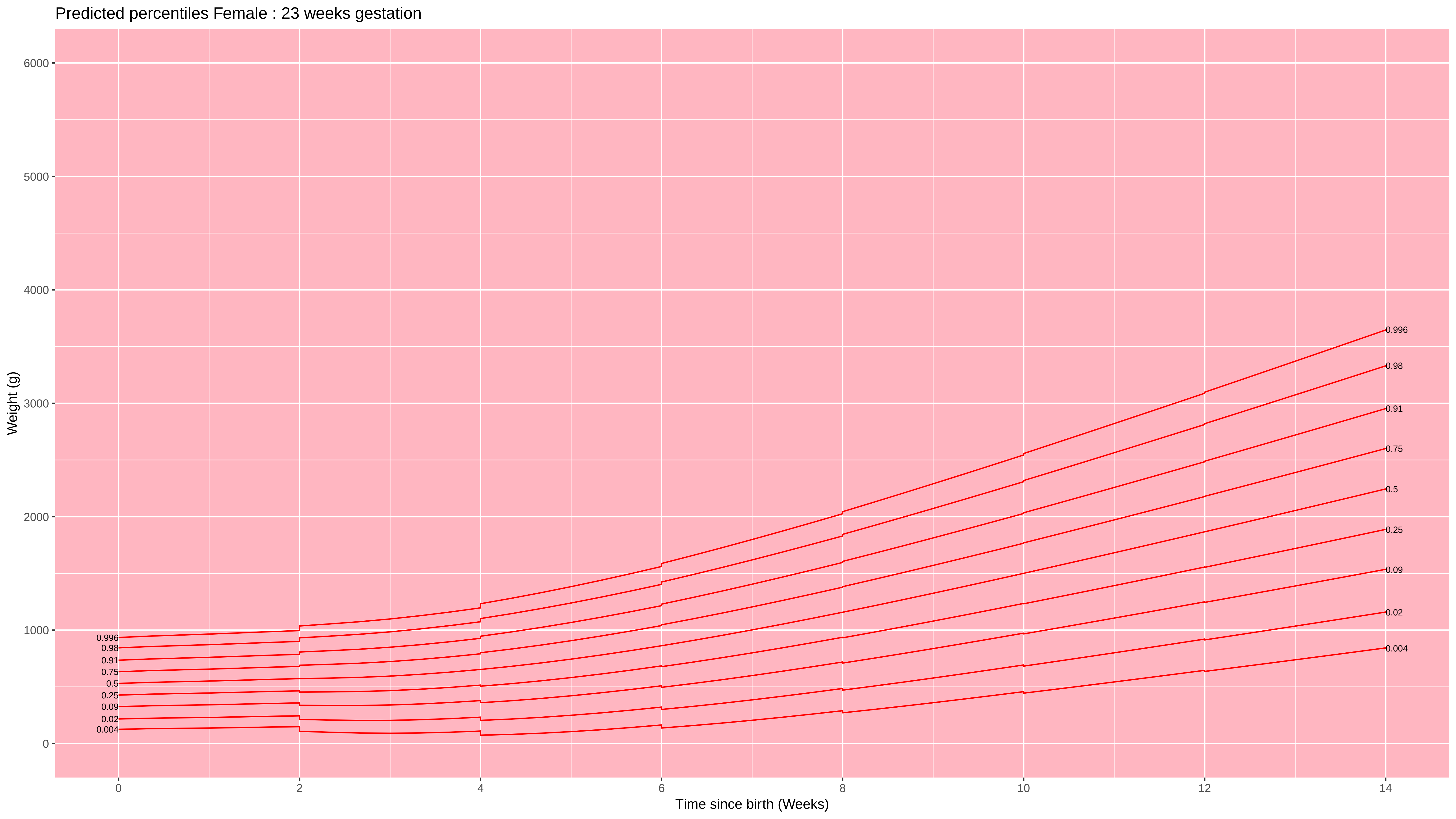

Predicted percentiles Female : 23 weeks gestation
Weight (g)
6000
5000
4000
3000
2000
1000
0
Time since birth (Weeks)
0
2
4
6
8
10
12
14
0.996
0.98
0.91
0.75
0.5
0.25
0.09
0.02
0.004

Predicted percentiles with Test data Female : 23 weeks gestation

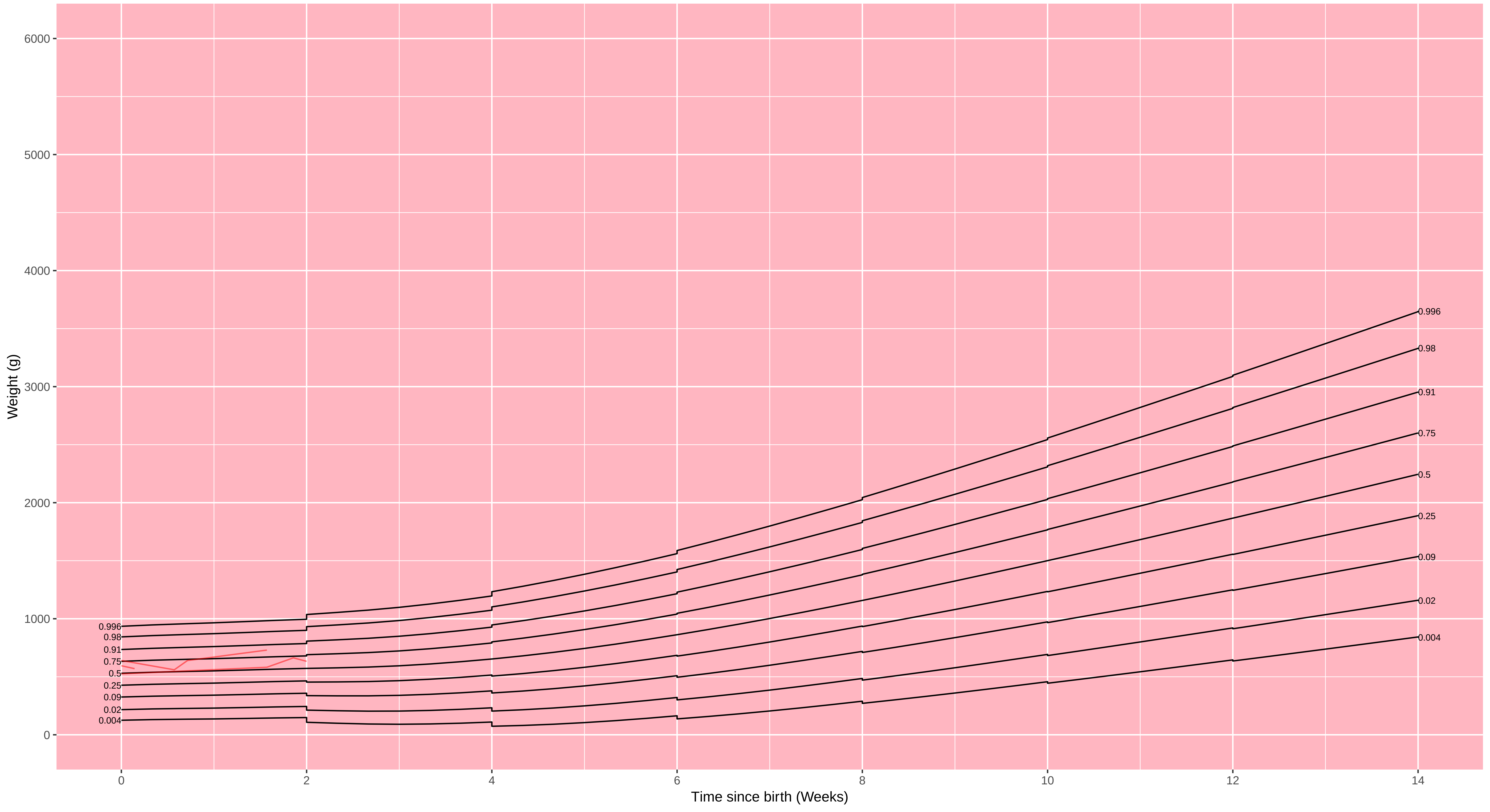

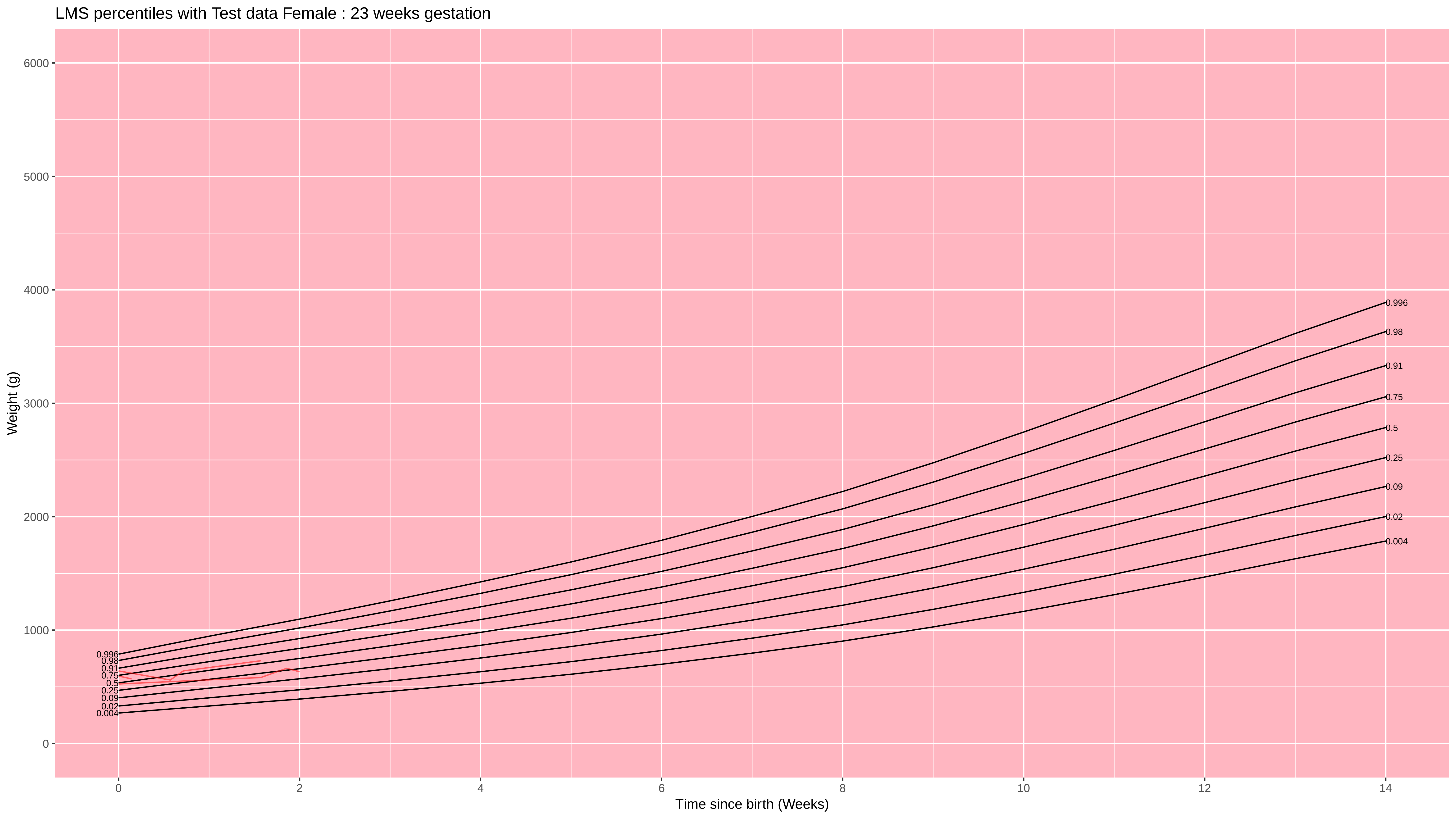
LMS percentiles with Test data Female : 23 weeks gestation
Weight (g)
Time since birth (Weeks)
0
1000
2000
3000
4000
5000
6000
0
2
4
6
8
10
12
14
0.996
0.98
0.91
0.75
0.5
0.25
0.09
0.02
0.004

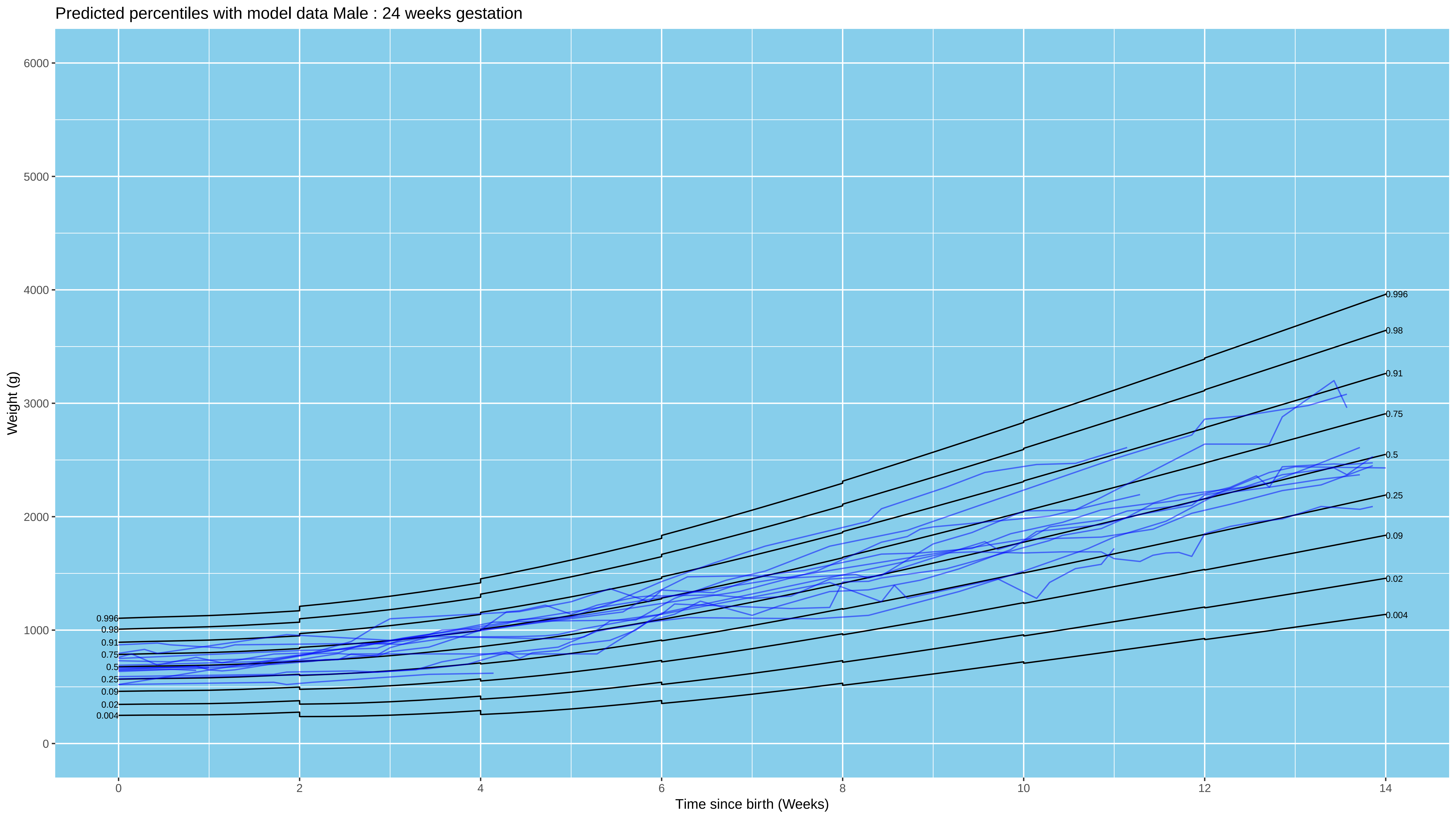

Predicted percentiles with model data Male : 24 weeks gestation
Weight (g)
Time since birth (Weeks)
0
1000
2000
3000
4000
5000
6000
0
2
4
6
8
10
12
14
0.996
0.98
0.91
0.75
0.5
0.25
0.09
0.02
0.004

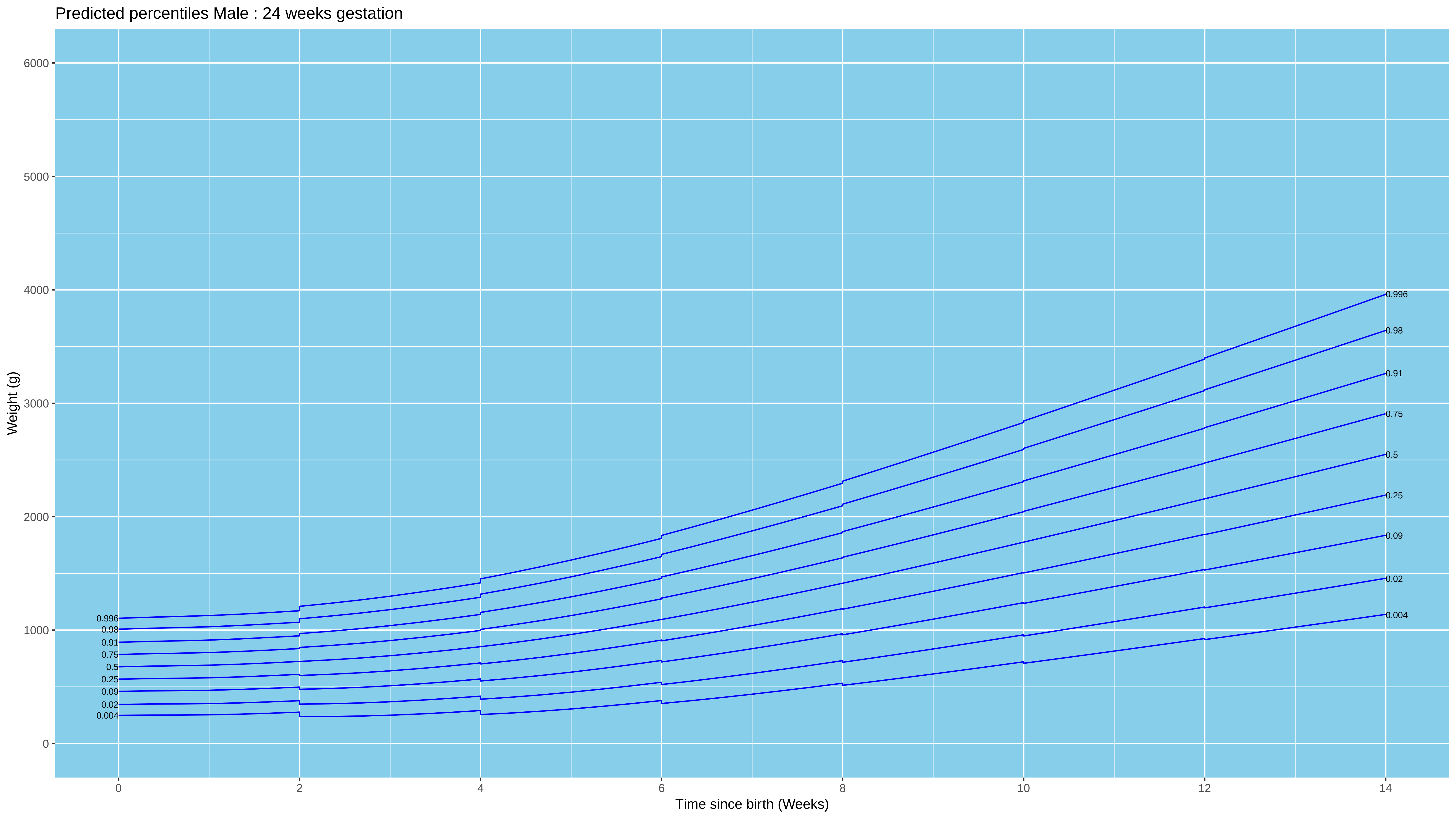

Predicted percentiles Male : 24 weeks gestation
Weight (g)
Time since birth (Weeks)
0
1000
2000
3000
4000
5000
6000
0
2
4
6
8
10
12
14
0.996
0.98
0.91
0.75
0.5
0.25
0.09
0.02
0.004

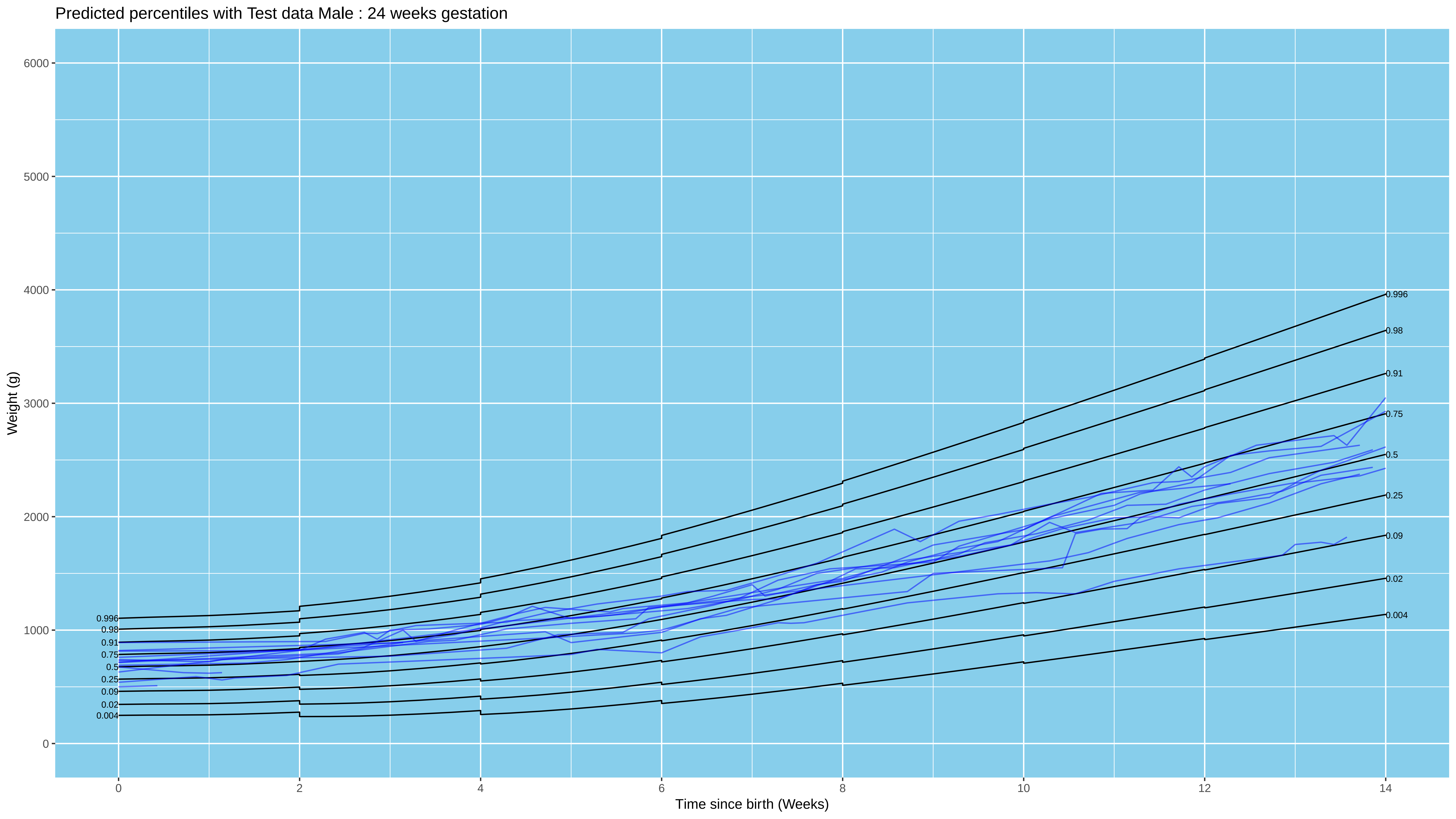
Predicted percentiles with Test data Male : 24 weeks gestation
Weight (g)
Time since birth (Weeks)
0
1000
2000
3000
4000
5000
6000
0
2
4
6
8
10
12
14
0.996
0.98
0.91
0.75
0.5
0.25
0.09
0.02
0.004

LMS percentiles with Test data Male : 24 weeks gestation

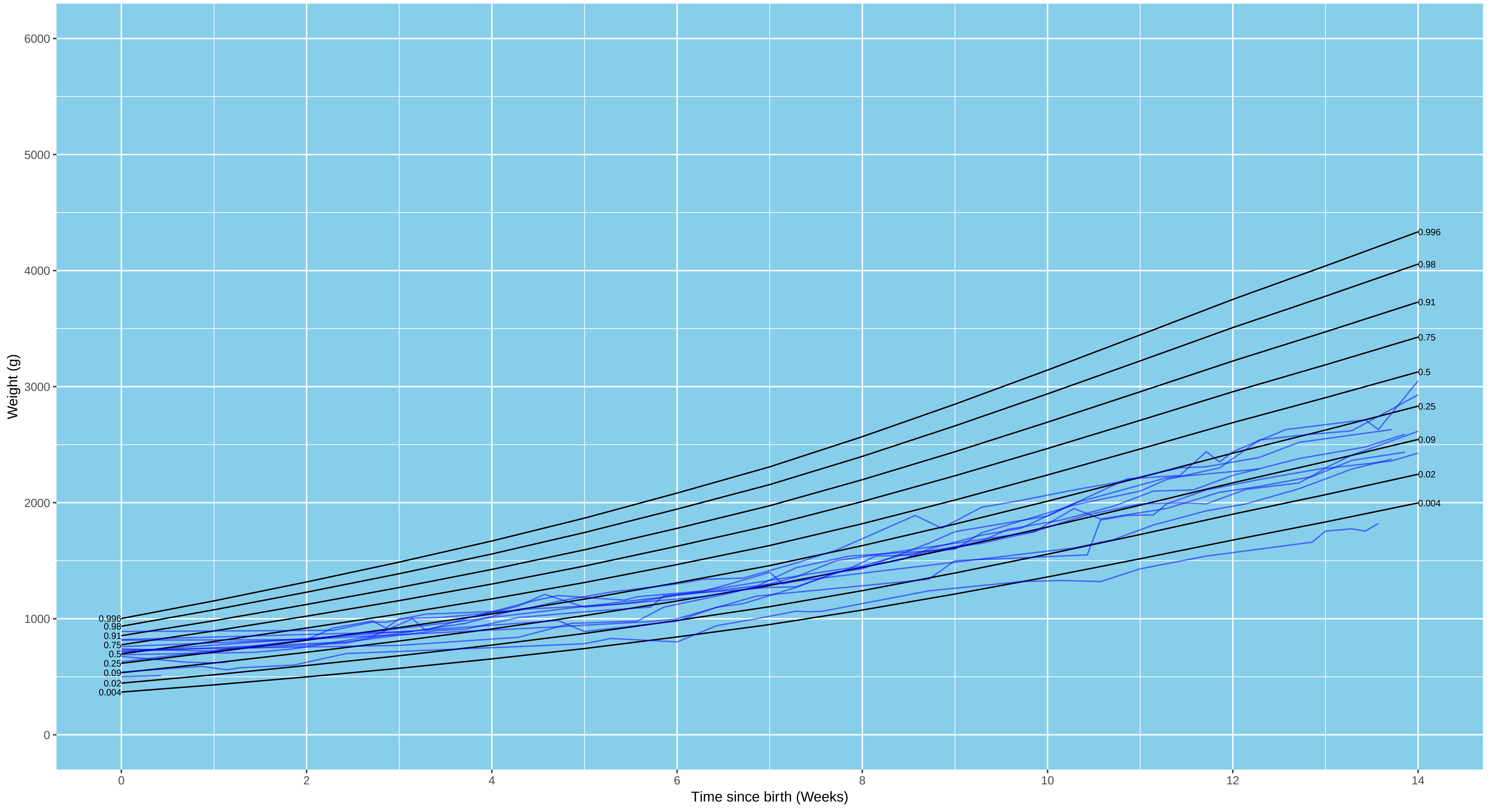

Predicted percentiles with model data Female : 24 weeks gestation

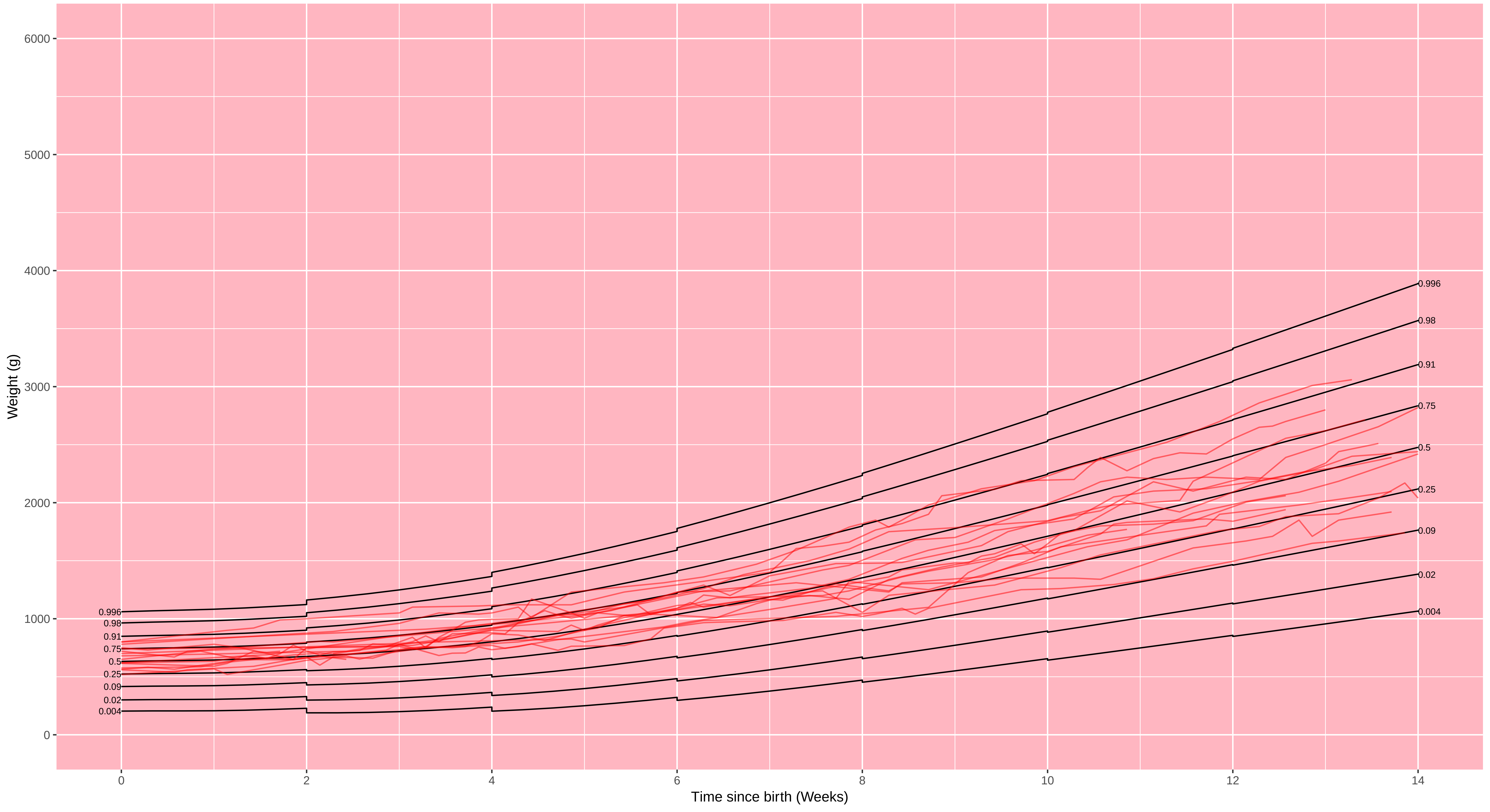

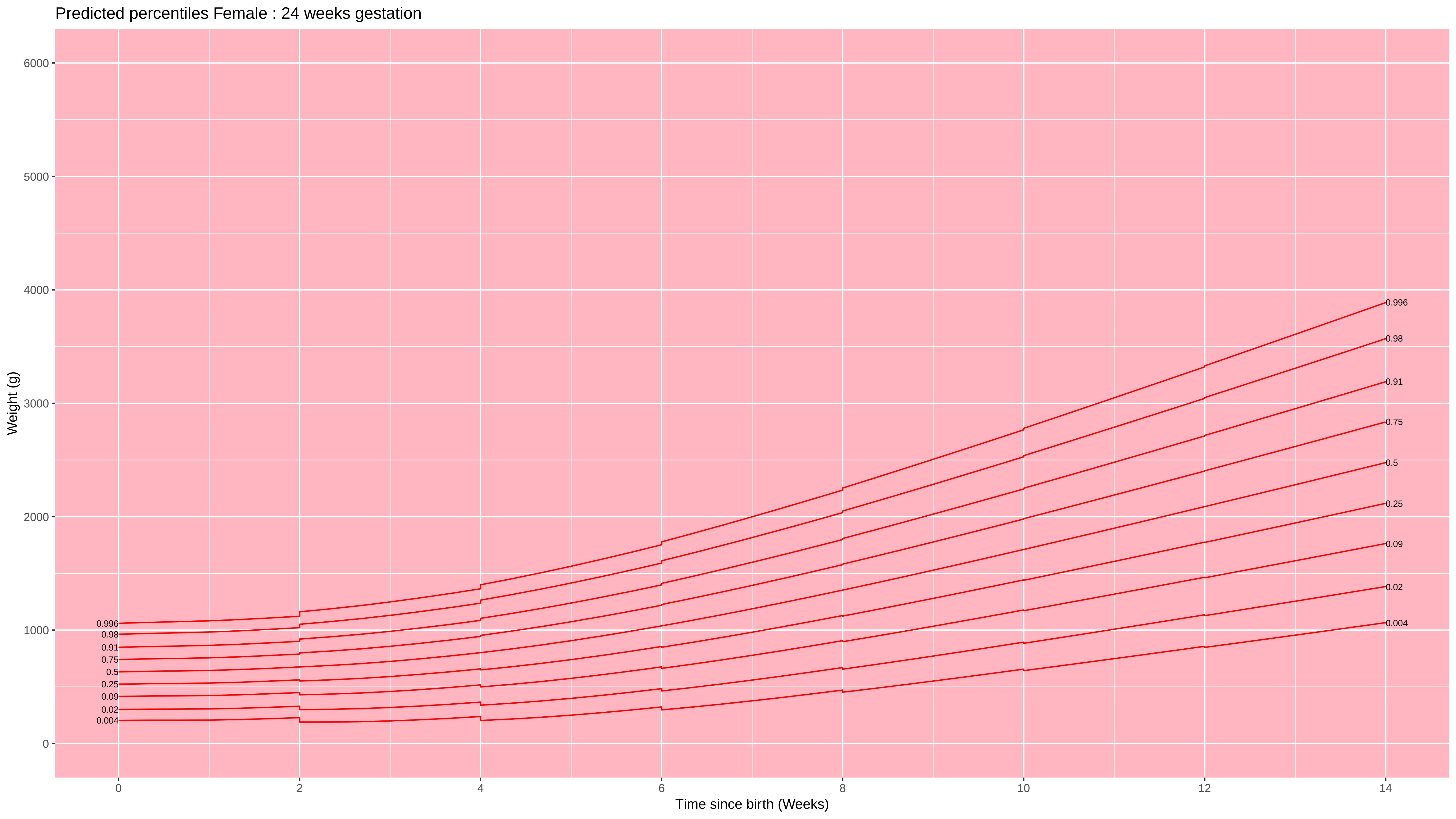
Predicted percentiles Female : 24 weeks gestation
Weight (g)
6000
5000
4000
3000
2000
1000
0
0.996
0.98
0.91
0.75
0.5
0.25
0.09
0.02
0.004
0
2
4
6
8
10
12
14
Time since birth (Weeks)

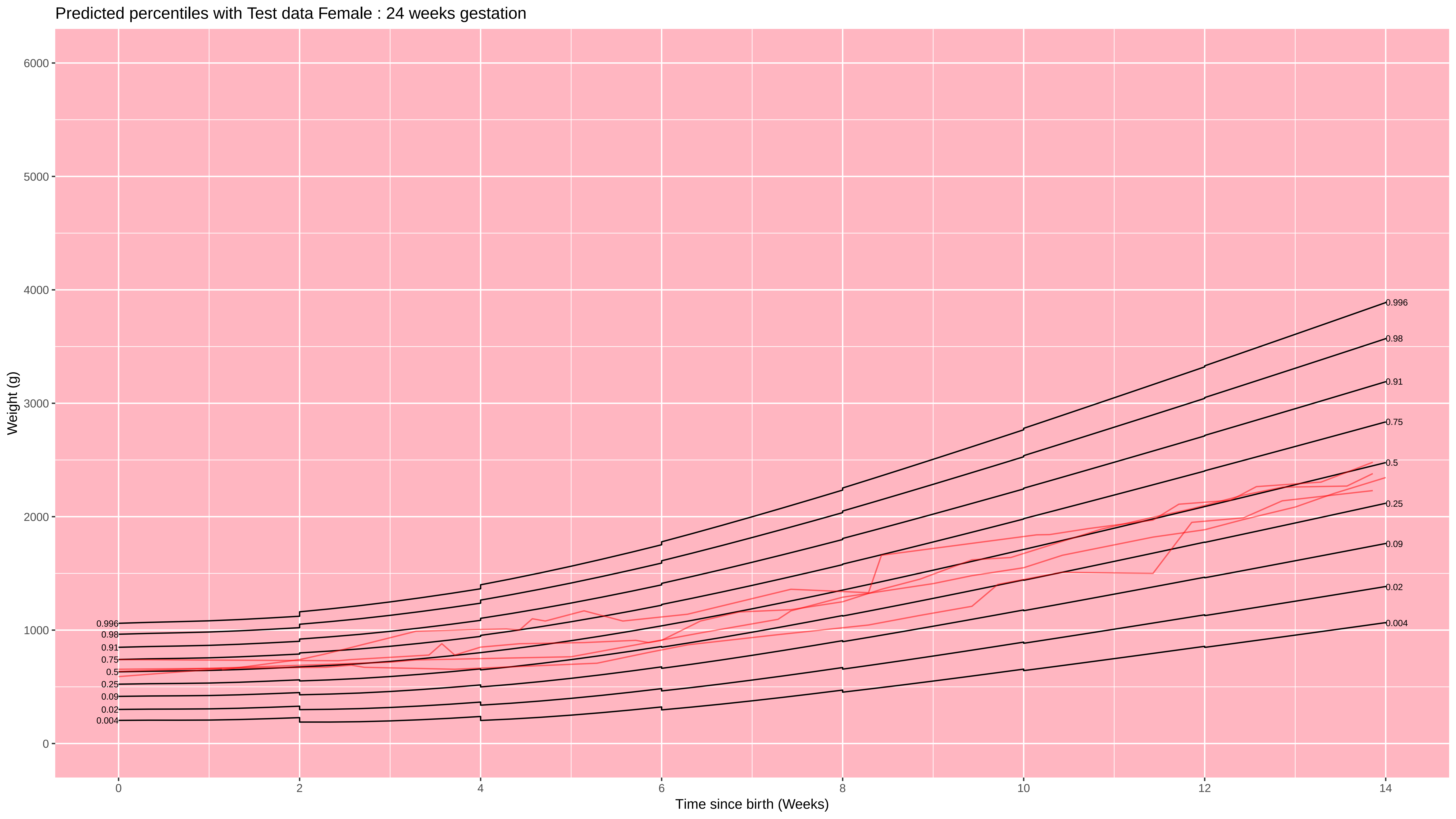

Predicted percentiles with Test data Female : 24 weeks gestation
Weight (g)
Time since birth (Weeks)
0
1000
2000
3000
4000
5000
6000
0
2
4
6
8
10
12
14
0.996
0.98
0.91
0.75
0.5
0.25
0.09
0.02
0.004

LMS percentiles with Test data Female : 24 weeks gestation

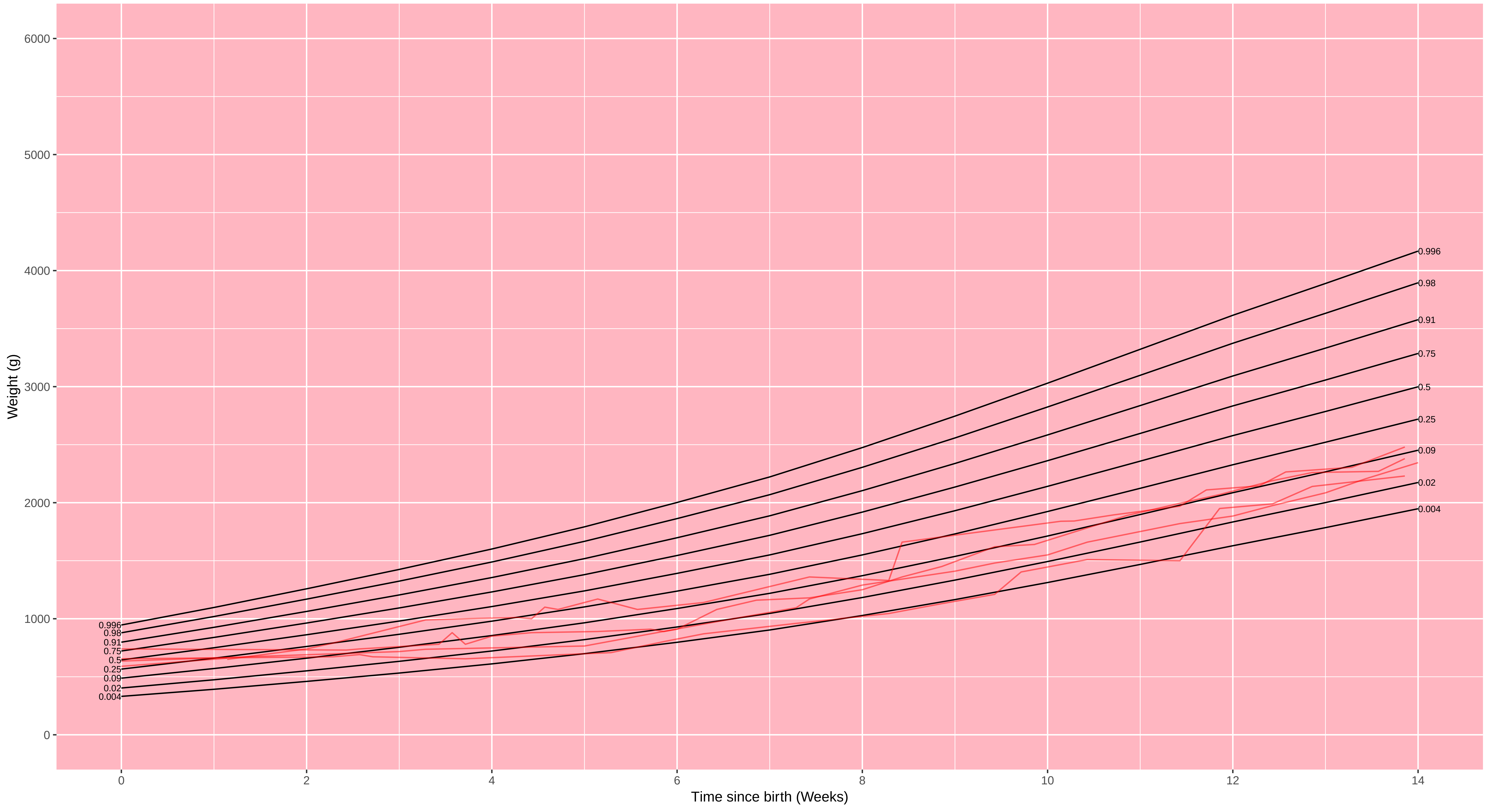

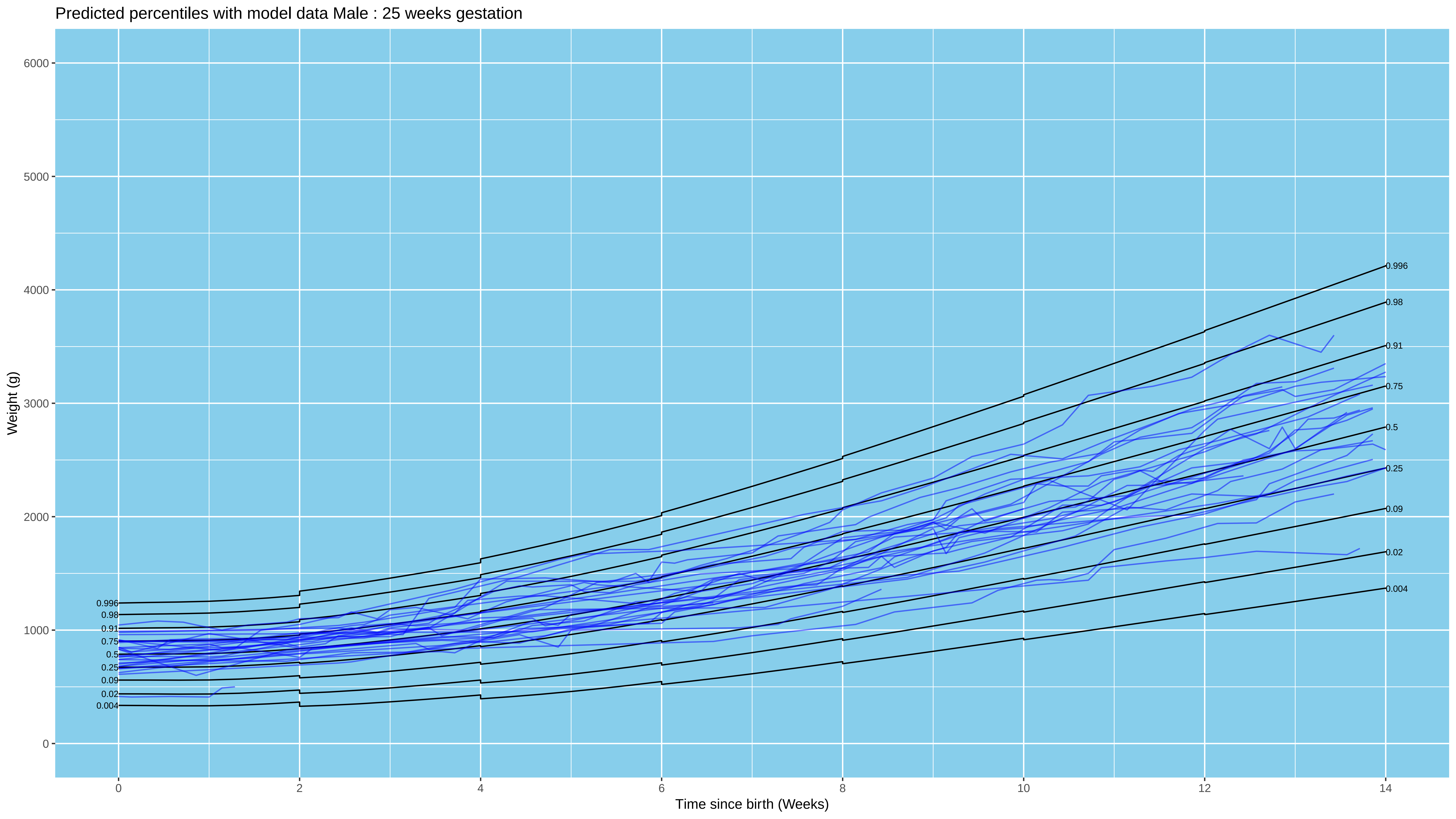
Predicted percentiles with model data Male : 25 weeks gestation
Weight (g)
Time since birth (Weeks)
0
1000
2000
3000
4000
5000
6000
0
2
4
6
8
10
12
14
0.996
0.98
0.91
0.75
0.5
0.25
0.09
0.02
0.004

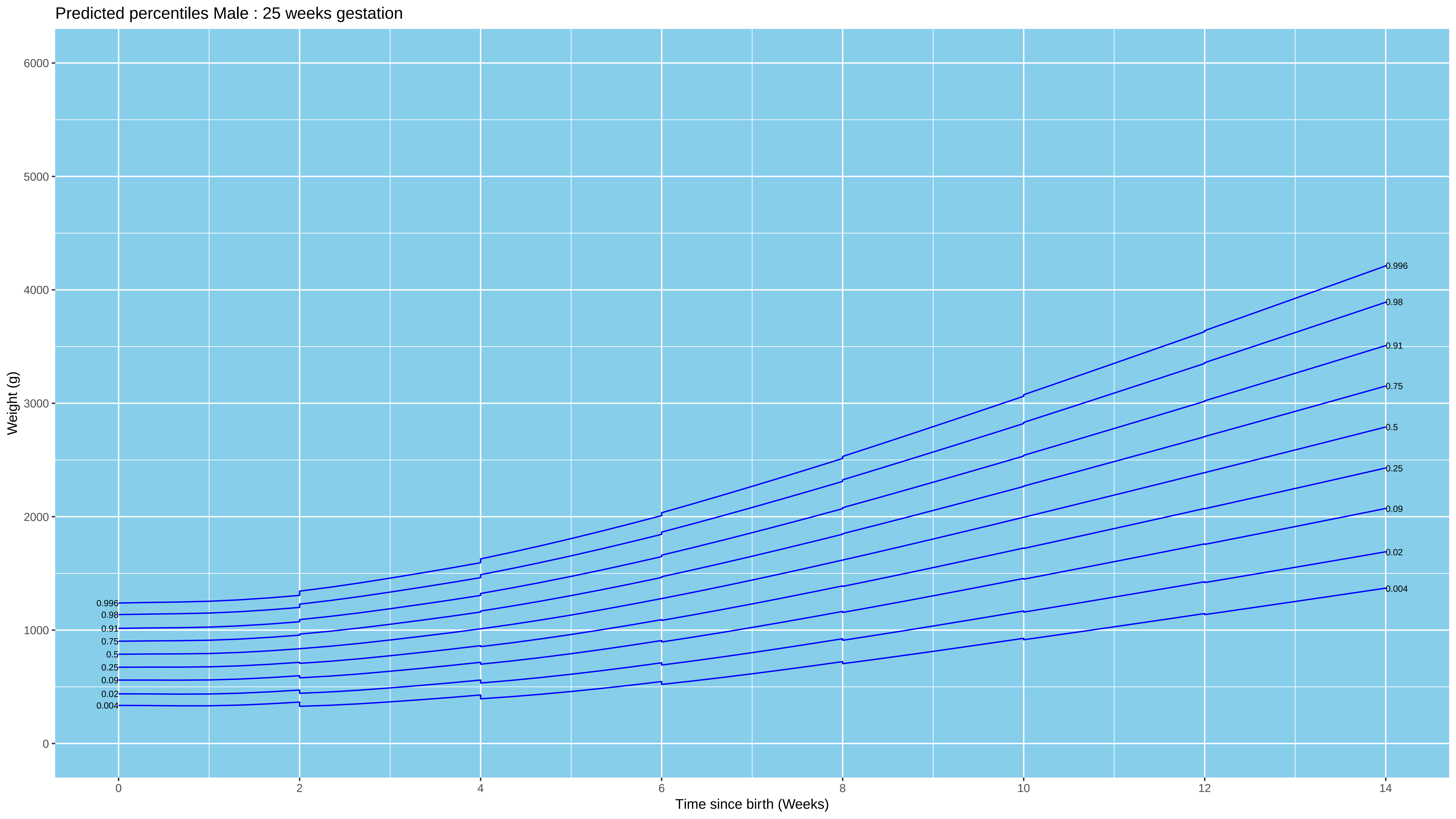

Predicted percentiles Male : 25 weeks gestation
Weight (g)
0
1000
2000
3000
4000
5000
6000
Time since birth (Weeks)
0
2
4
6
8
10
12
14
0.996
0.98
0.91
0.75
0.5
0.25
0.09
0.02
0.004

Predicted percentiles with Test data Male : 25 weeks gestation

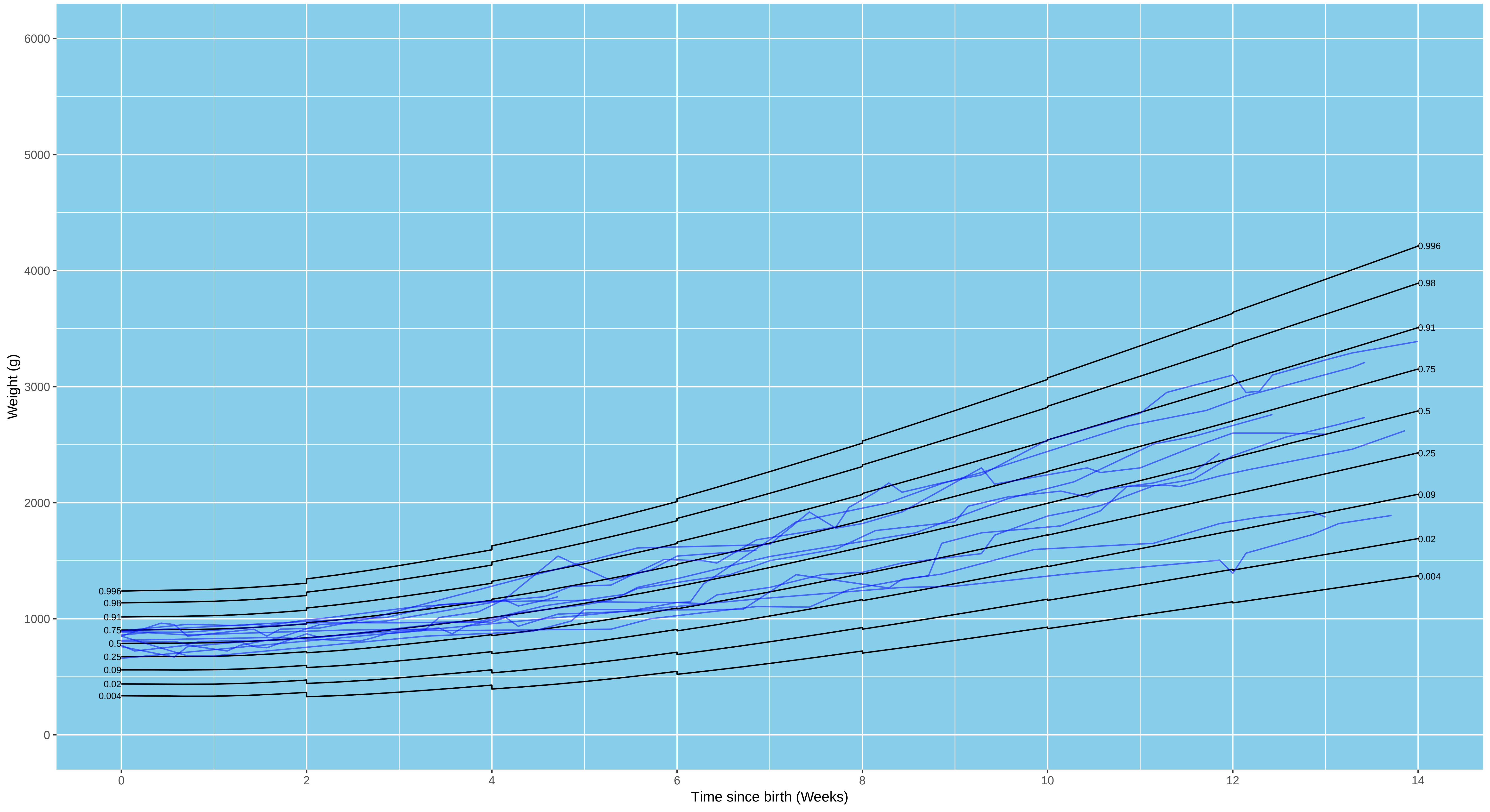

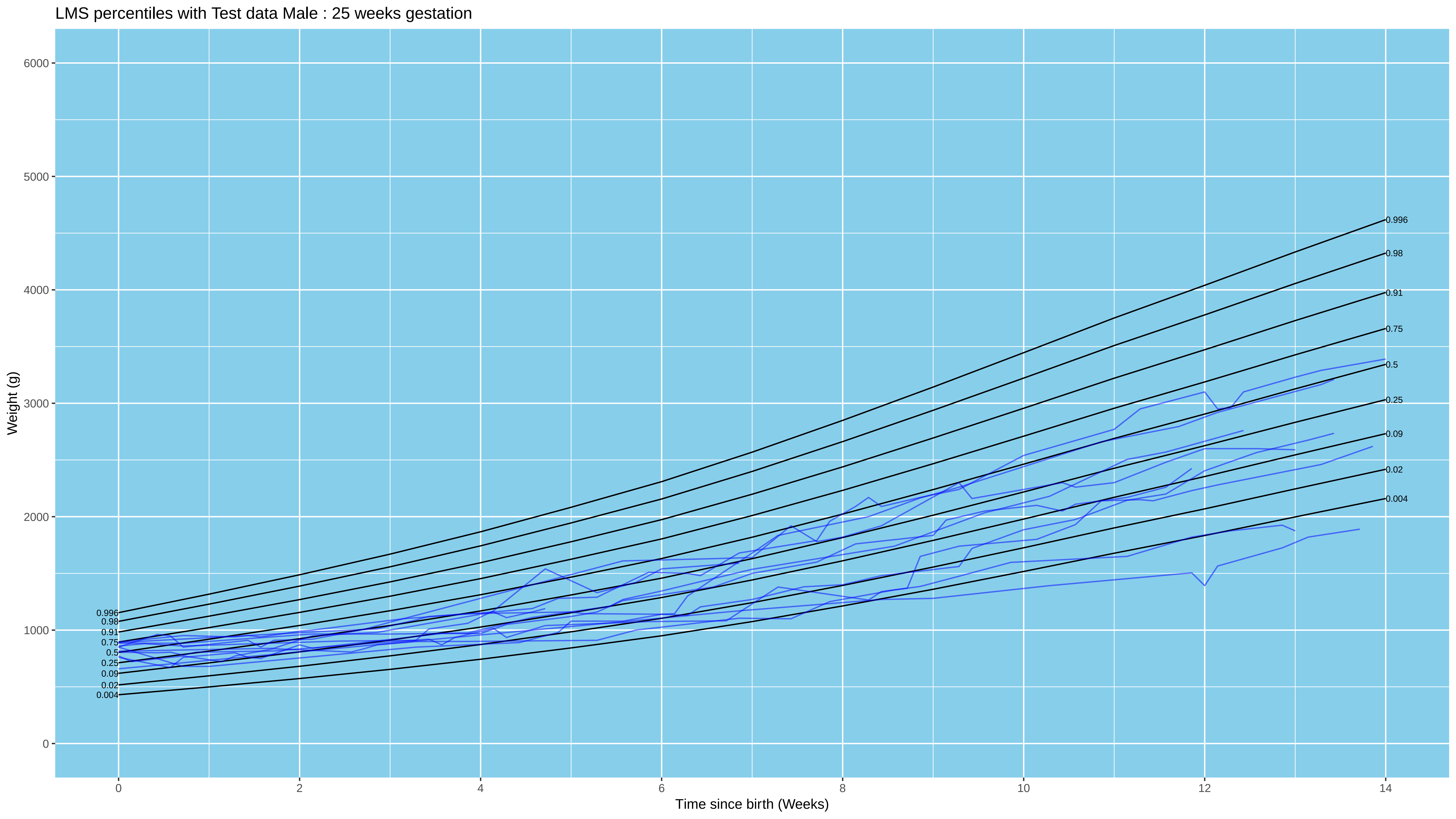

LMS percentiles with Test data Male : 25 weeks gestation
Weight (g)
Time since birth (Weeks)
0
1000
2000
3000
4000
5000
6000
0
2
4
6
8
10
12
14
0.996
0.98
0.91
0.75
0.5
0.25
0.09
0.02
0.004

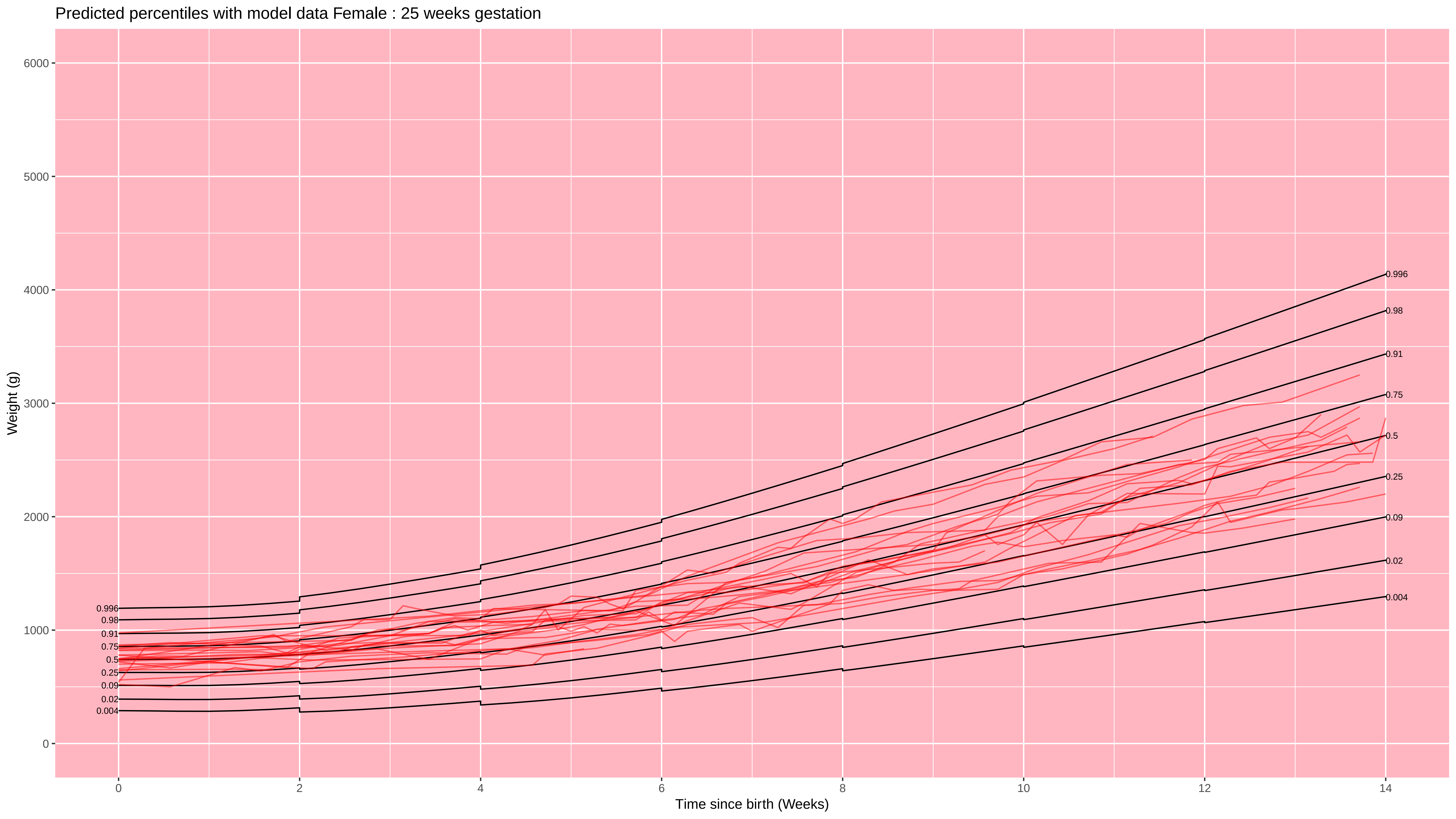

Predicted percentiles with model data Female : 25 weeks gestation
Weight (g)
Time since birth (Weeks)
0
1000
2000
3000
4000
5000
6000
0
2
4
6
8
10
12
14
0.996
0.98
0.91
0.75
0.5
0.25
0.09
0.02
0.004

Predicted percentiles Female : 25 weeks gestation

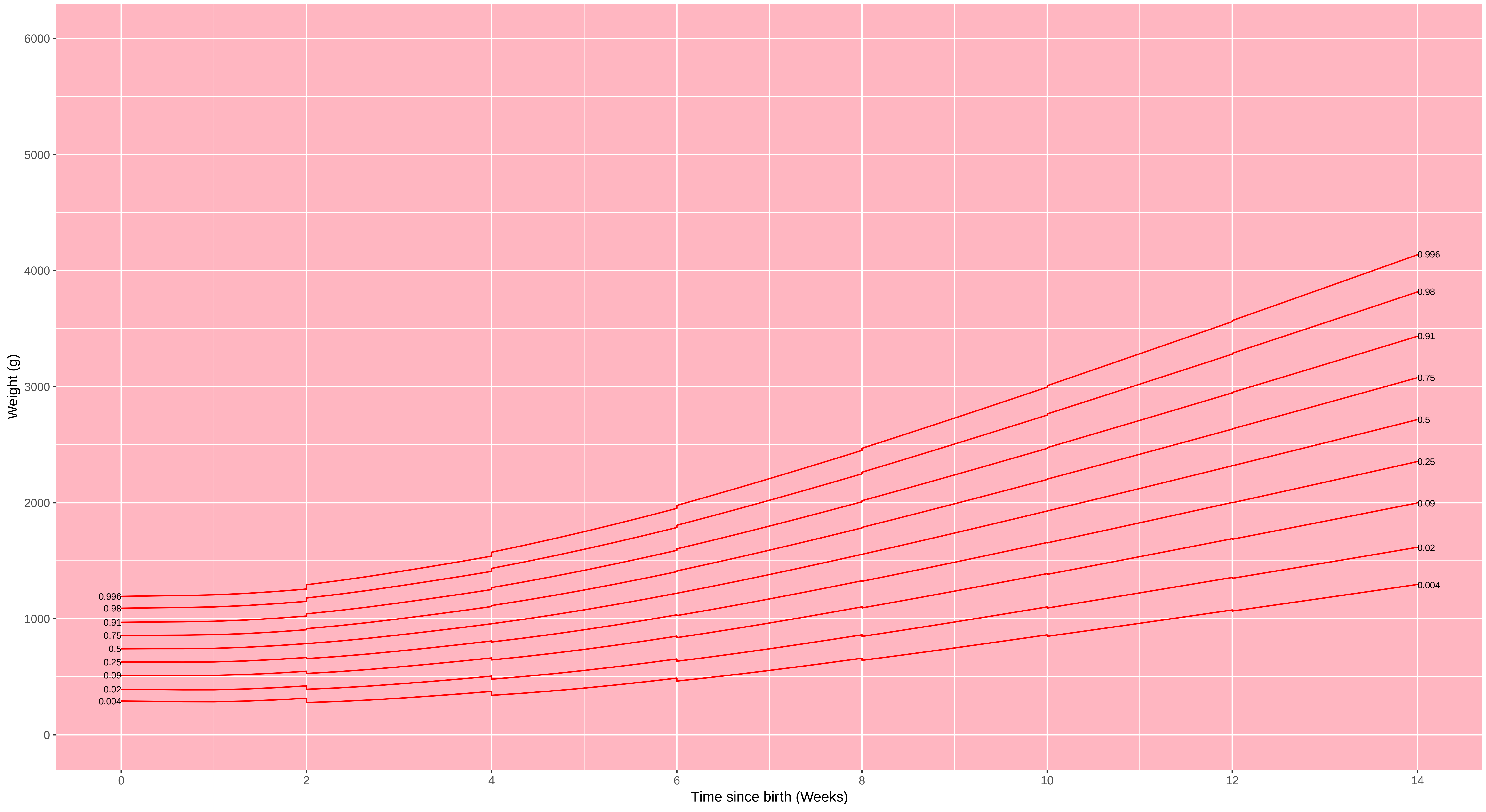

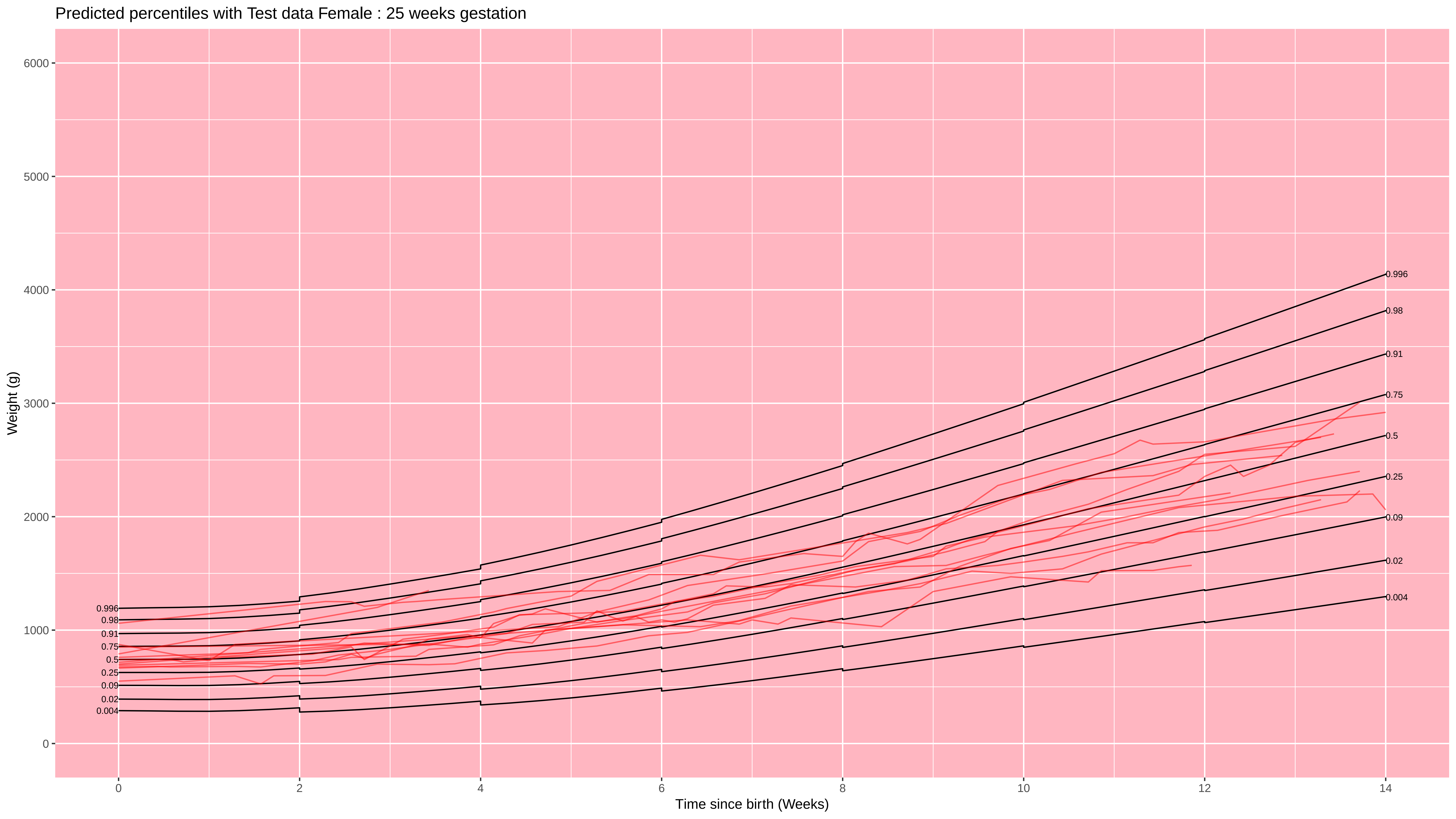

Predicted percentiles with Test data Female : 25 weeks gestation
Weight (g)
0
1000
2000
3000
4000
5000
6000
Time since birth (Weeks)
0
2
4
6
8
10
12
14
0.996
0.98
0.91
0.75
0.5
0.25
0.09
0.02
0.004

LMS percentiles with Test data Female : 25 weeks gestation

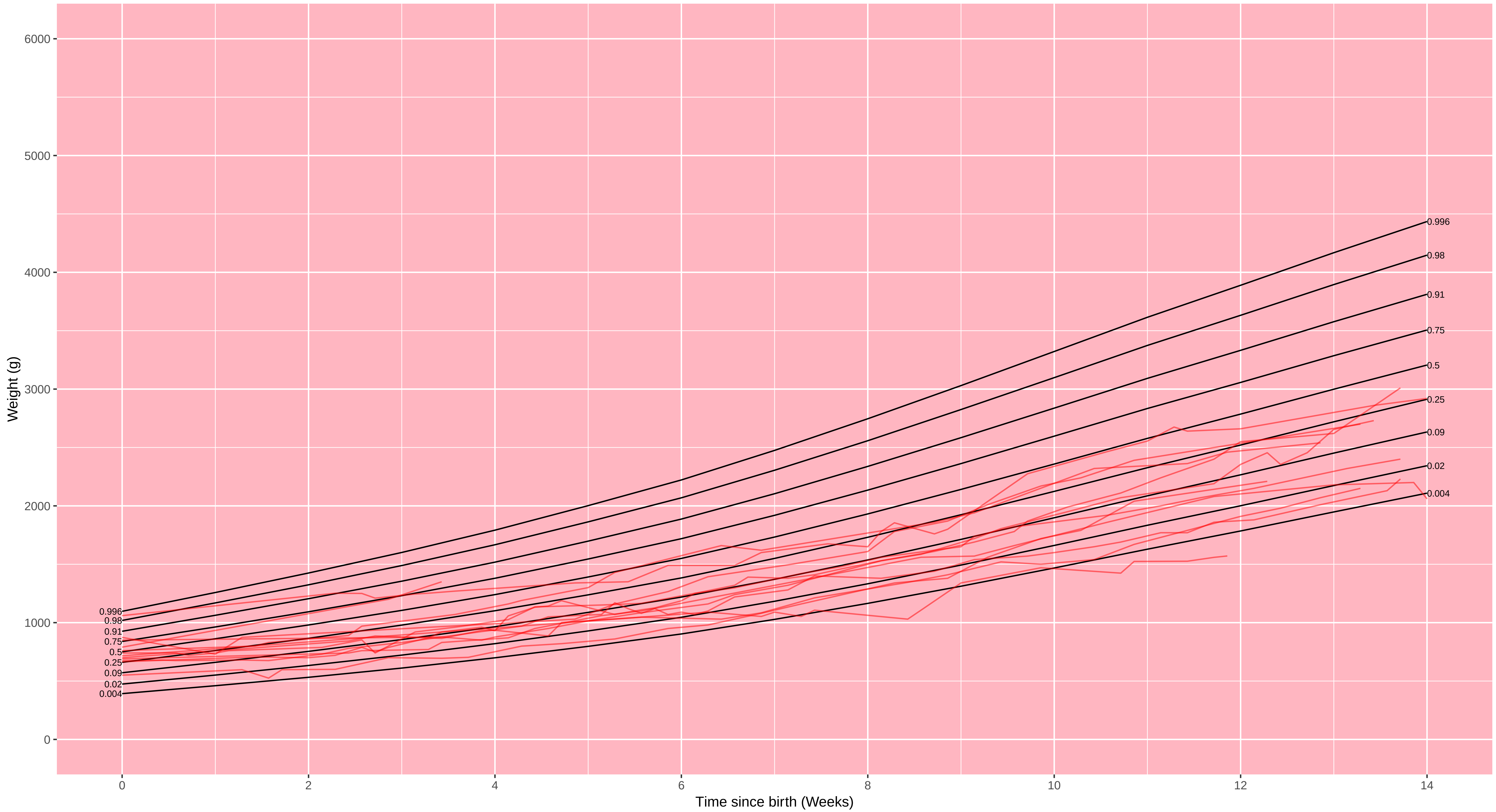

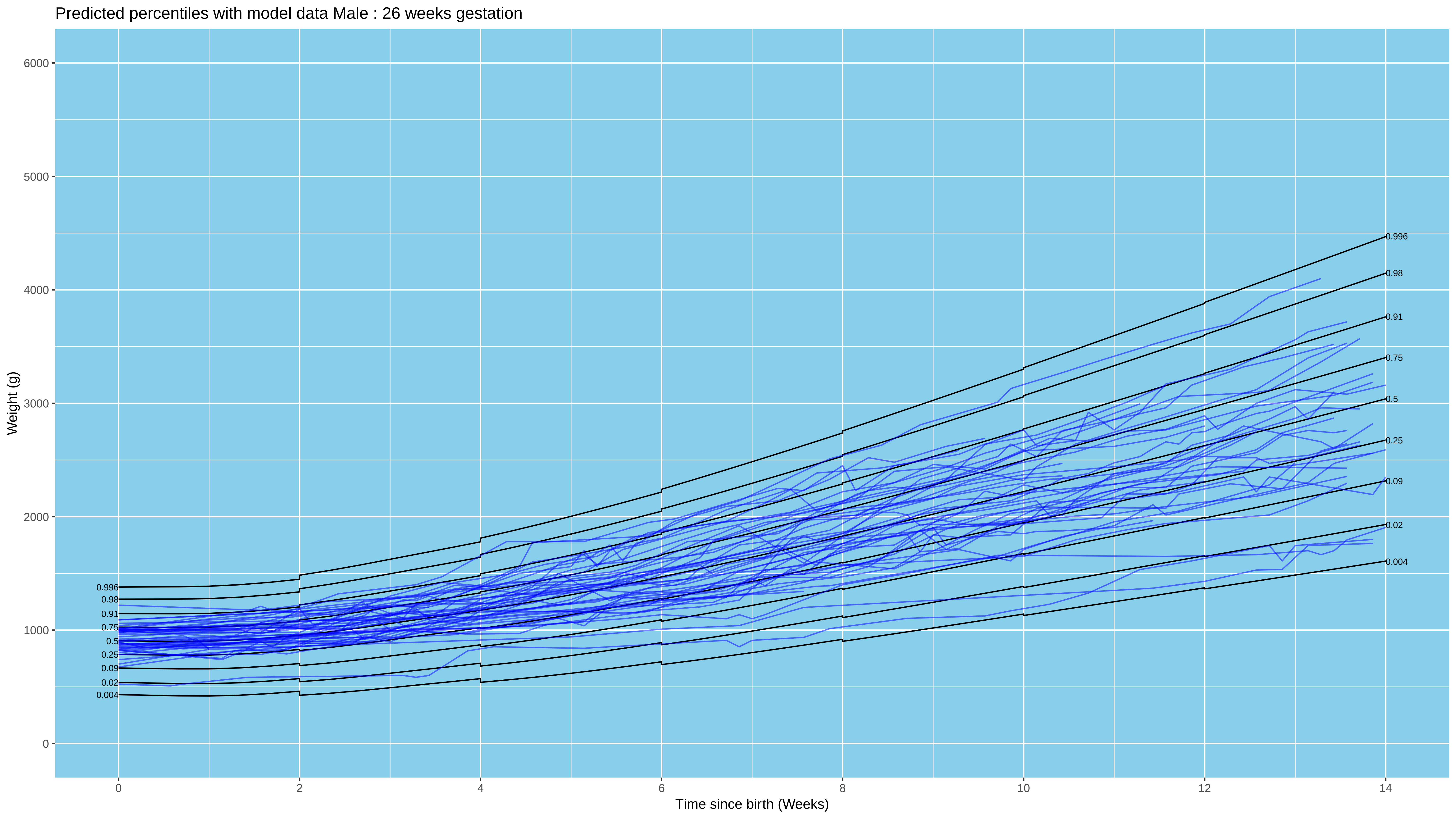
Predicted percentiles with model data Male : 26 weeks gestation
Weight (g)
Time since birth (Weeks)
0
1000
2000
3000
4000
5000
6000
0
2
4
6
8
10
12
14
0.996
0.98
0.91
0.75
0.5
0.25
0.09
0.02
0.004

Predicted percentiles Male : 26 weeks gestation

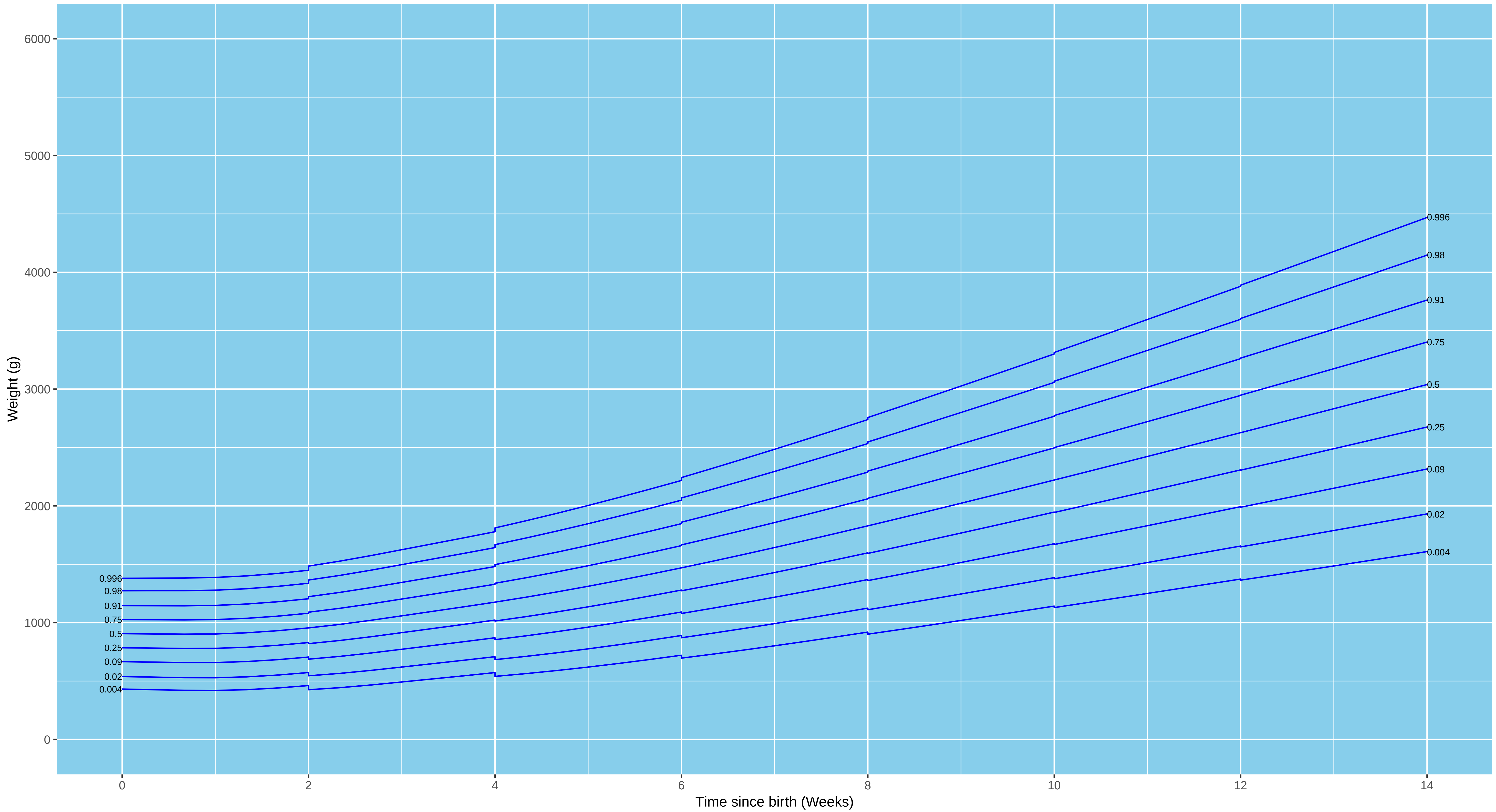

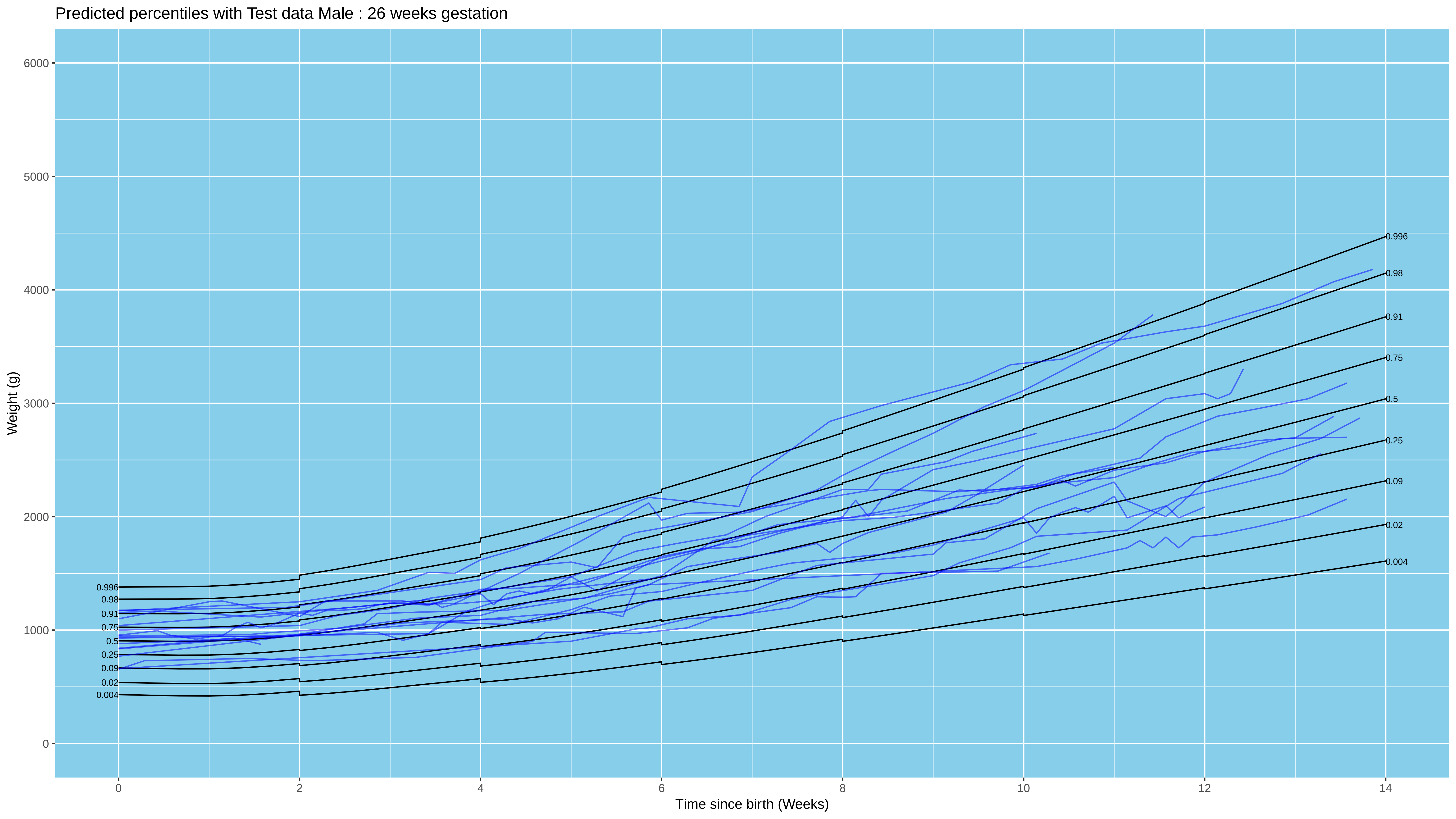
Predicted percentiles with Test data Male : 26 weeks gestation
Weight (g)
Time since birth (Weeks)
0
1000
2000
3000
4000
5000
6000
0
2
4
6
8
10
12
14
0.996
0.98
0.91
0.75
0.5
0.25
0.09
0.02
0.004

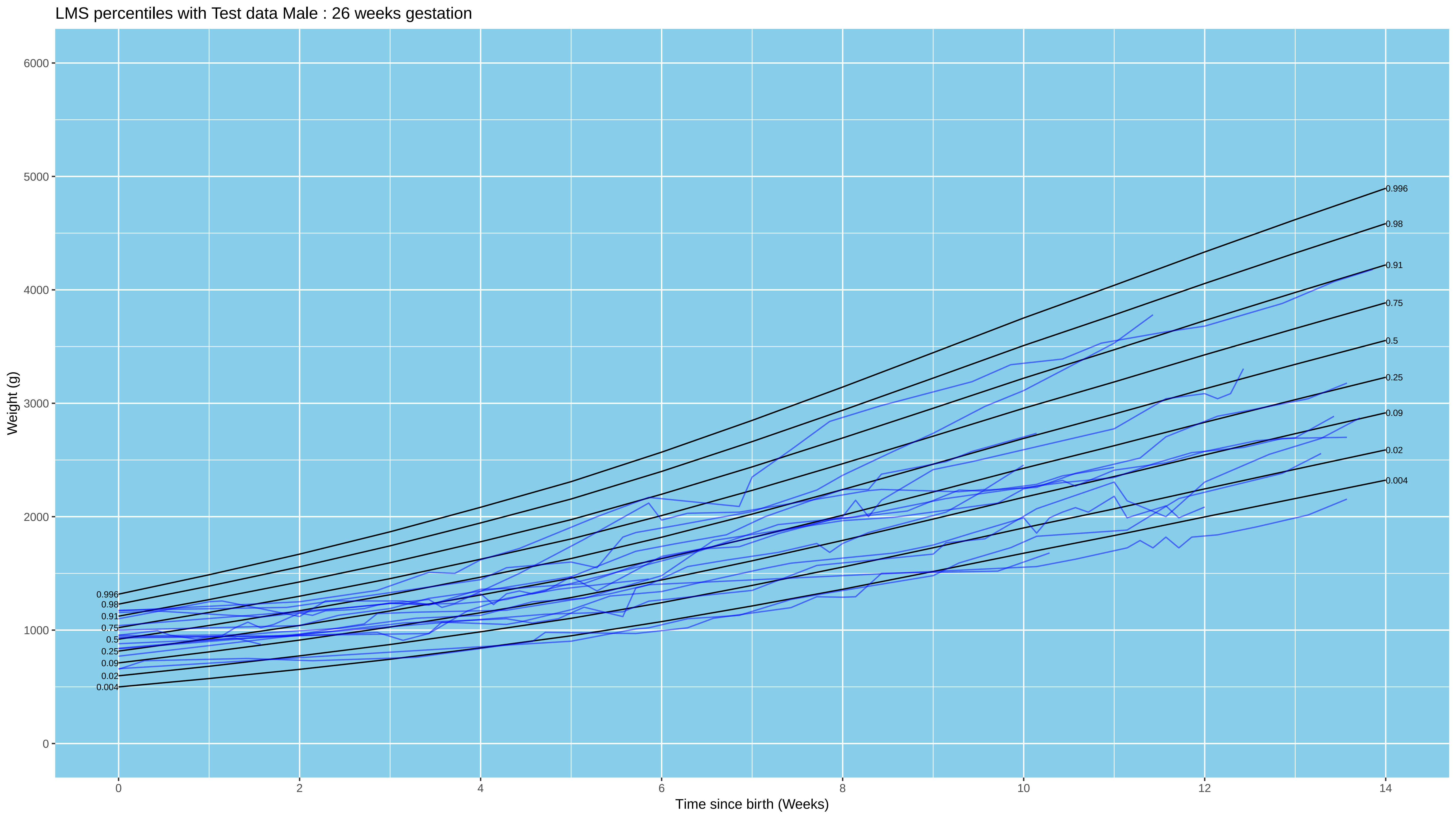

LMS percentiles with Test data Male : 26 weeks gestation
Weight (g)
Time since birth (Weeks)
0
1000
2000
3000
4000
5000
6000
0
2
4
6
8
10
12
14
0.996
0.98
0.91
0.75
0.5
0.25
0.09
0.02
0.004

Predicted percentiles with model data Female : 26 weeks gestation

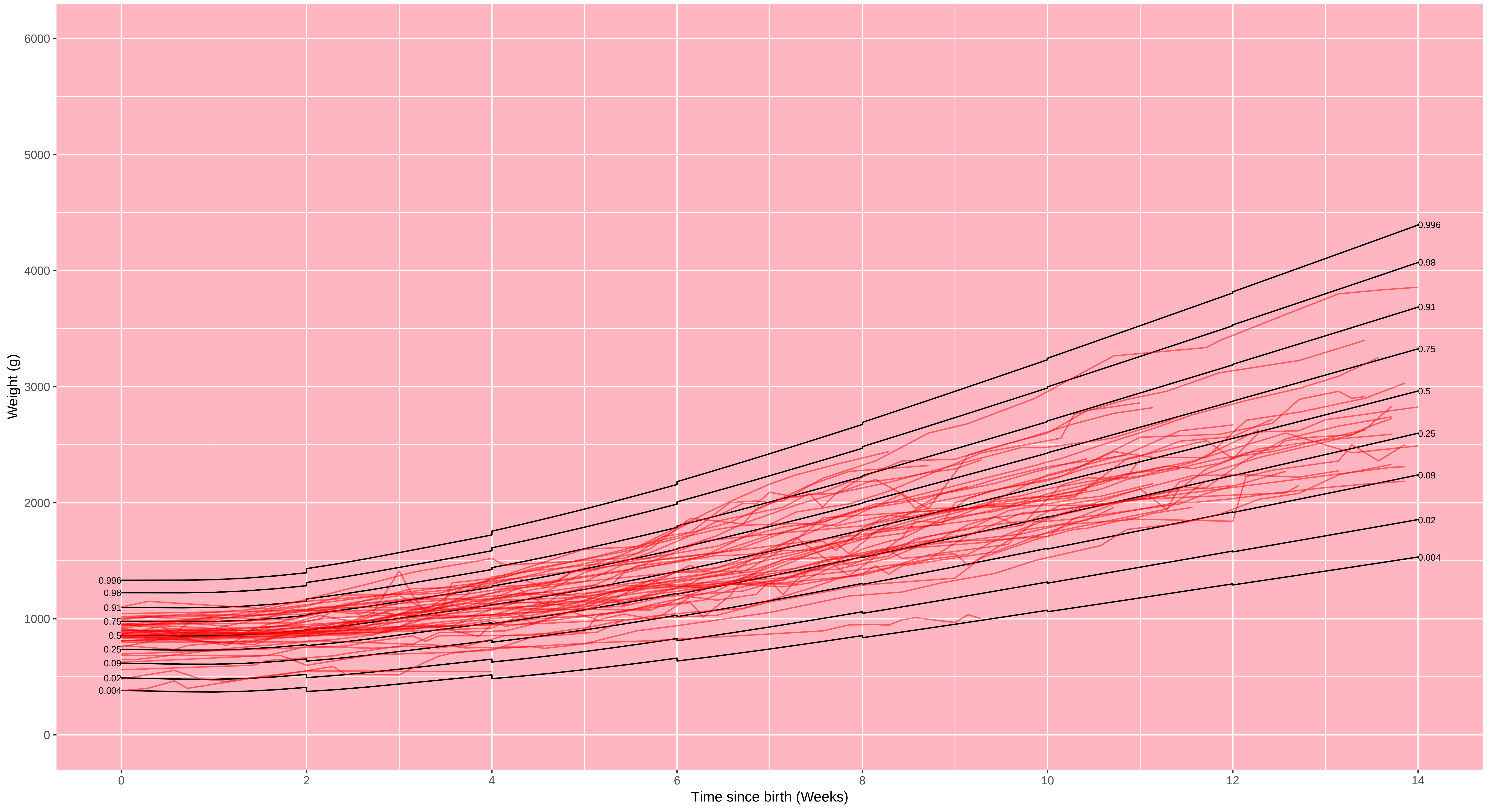

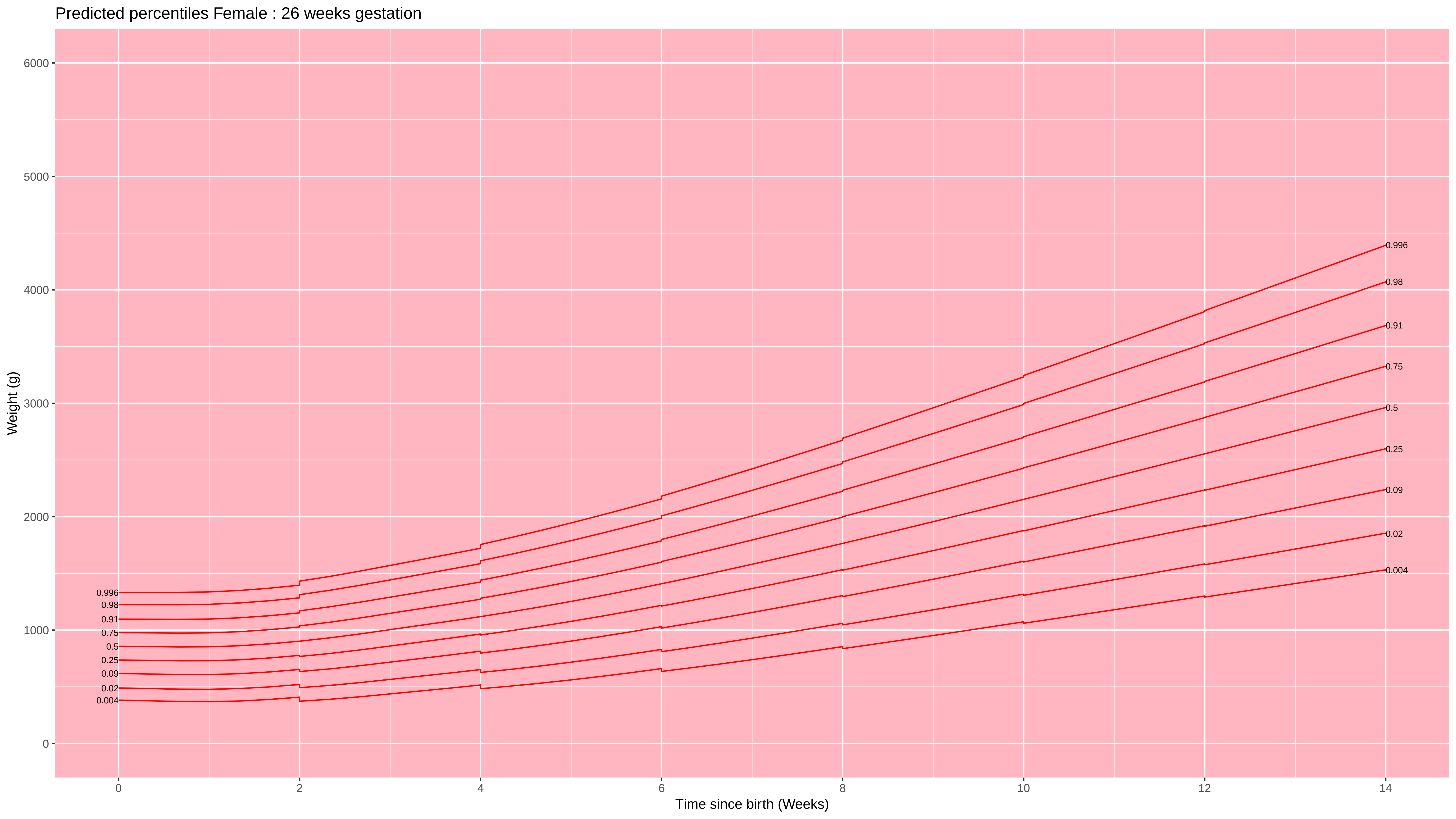

Predicted percentiles Female : 26 weeks gestation
Weight (g)
Time since birth (Weeks)
0
1000
2000
3000
4000
5000
6000
0
2
4
6
8
10
12
14
0.996
0.98
0.91
0.75
0.5
0.25
0.09
0.02
0.004
0.996
0.98
0.91
0.75
0.5
0.25
0.09
0.02
0.004

Predicted percentiles with Test data Female : 26 weeks gestation

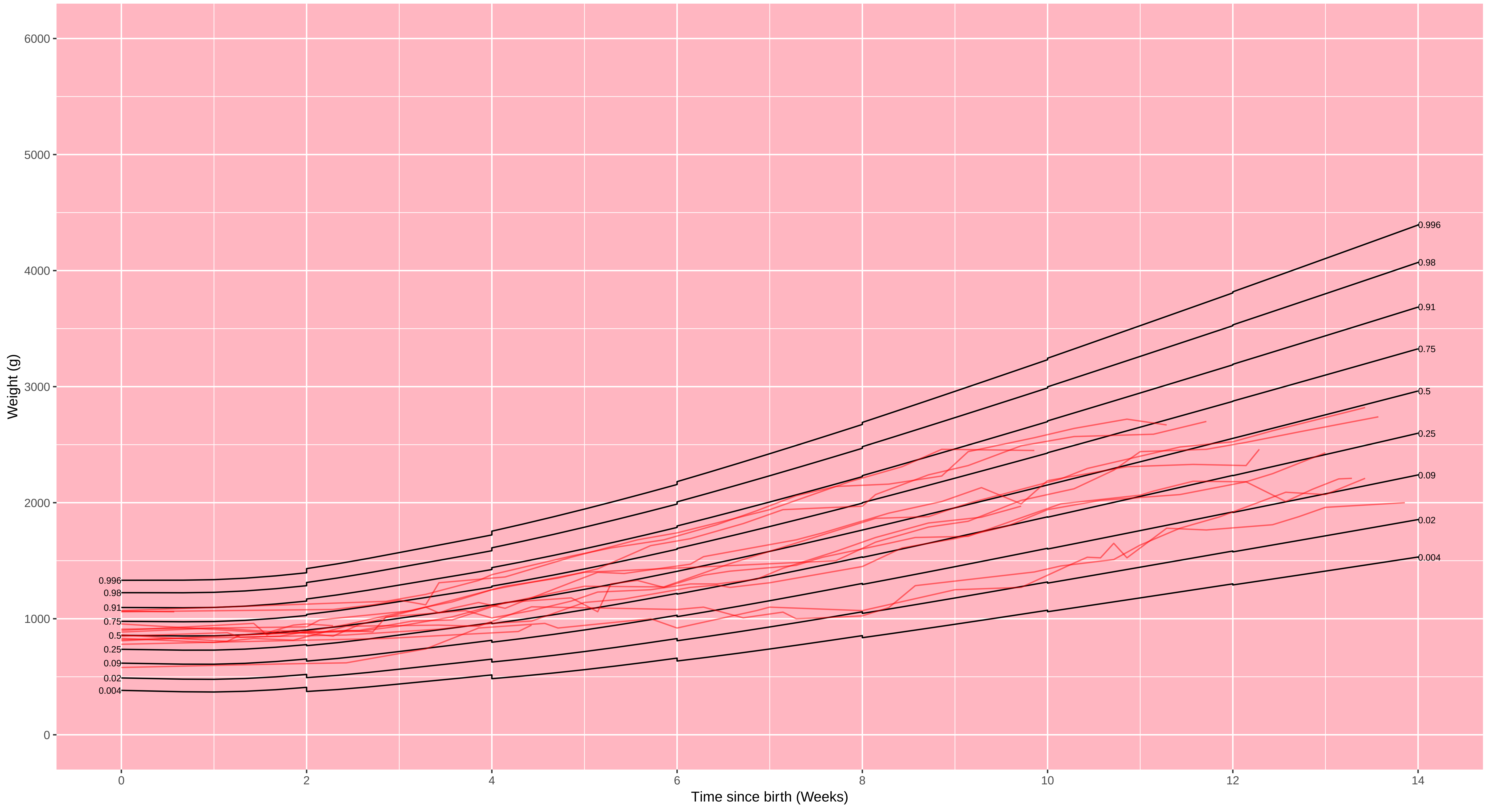

LMS percentiles with Test data Female : 26 weeks gestation

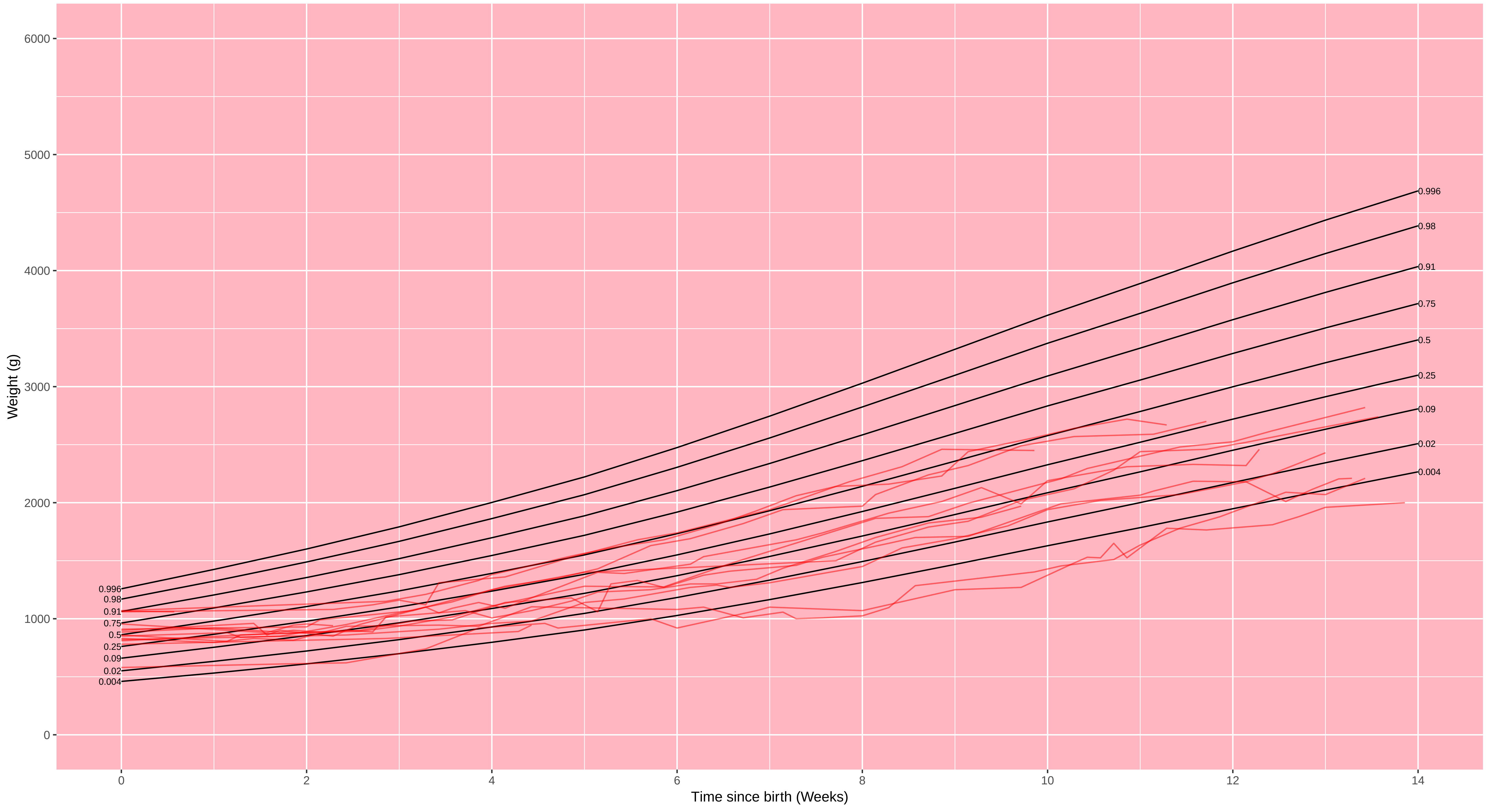

Predicted percentiles with model data Male : 27 weeks gestation

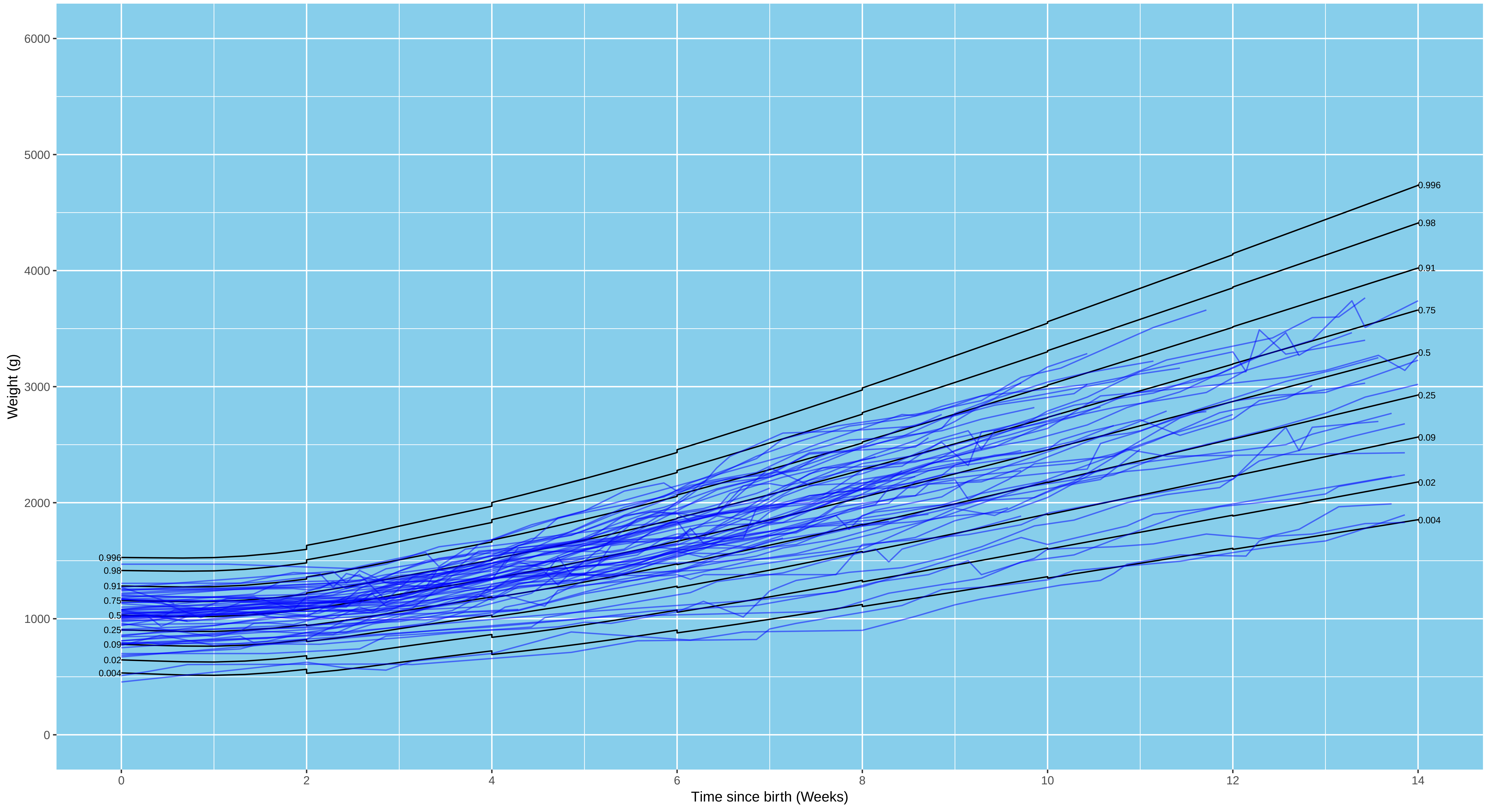

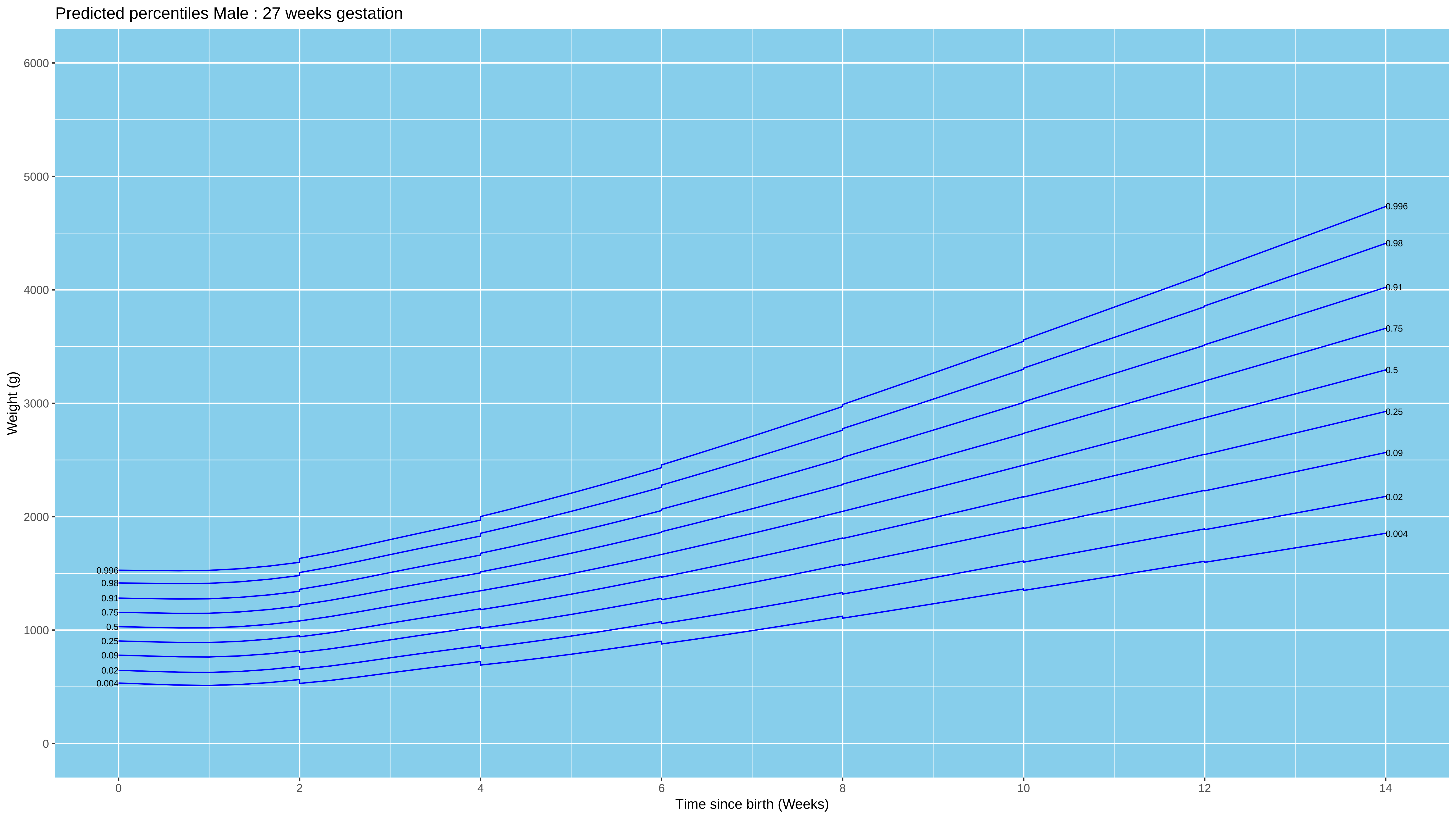

Predicted percentiles Male : 27 weeks gestation
Weight (g)
Time since birth (Weeks)
0
1000
2000
3000
4000
5000
6000
0
2
4
6
8
10
12
14
0.996
0.98
0.91
0.75
0.5
0.25
0.09
0.02
0.004

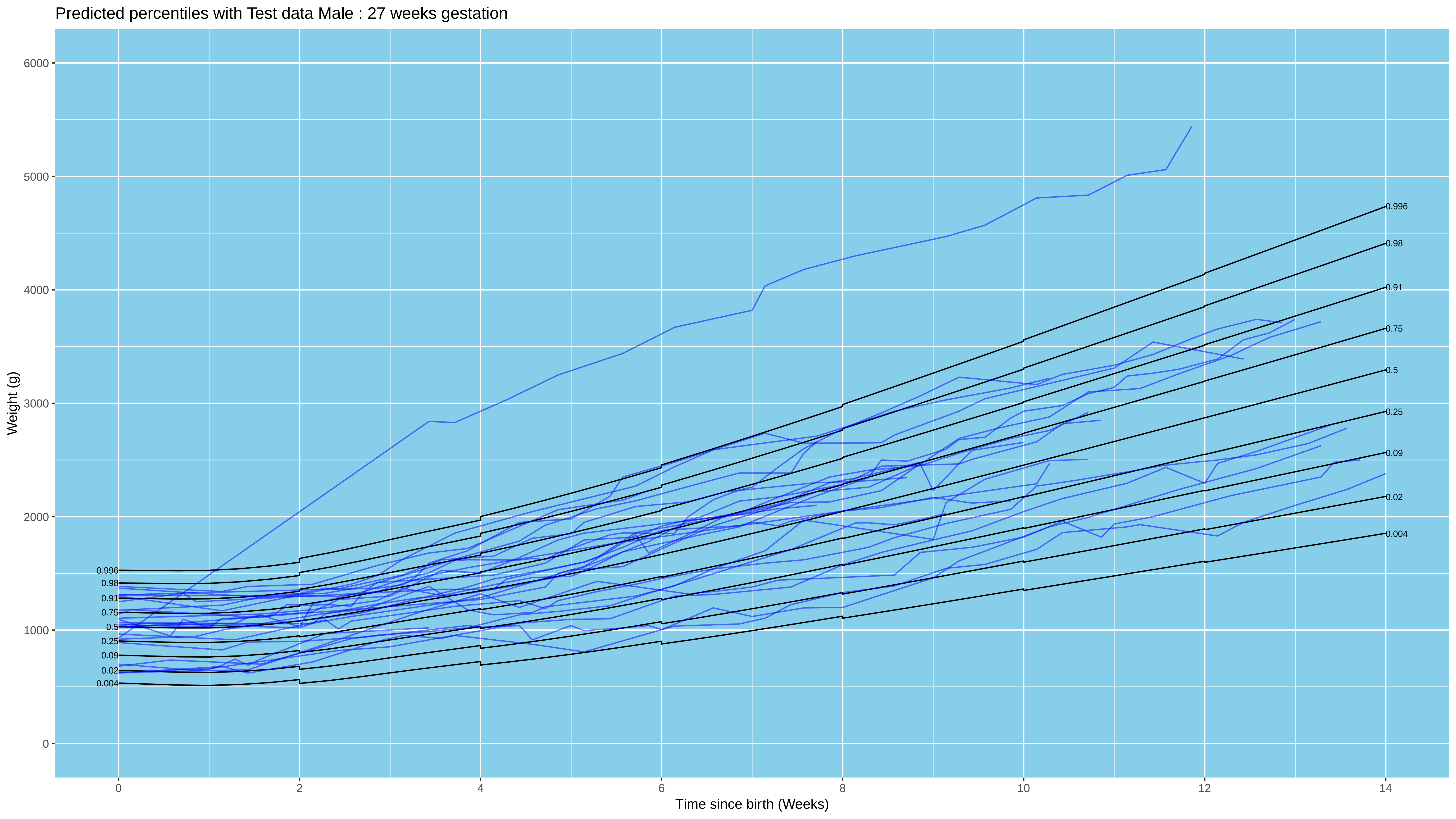
Predicted percentiles with Test data Male : 27 weeks gestation
Weight (g)
Time since birth (Weeks)
0
1000
2000
3000
4000
5000
6000
0
2
4
6
8
10
12
14
0.996
0.98
0.91
0.75
0.5
0.25
0.09
0.02
0.004

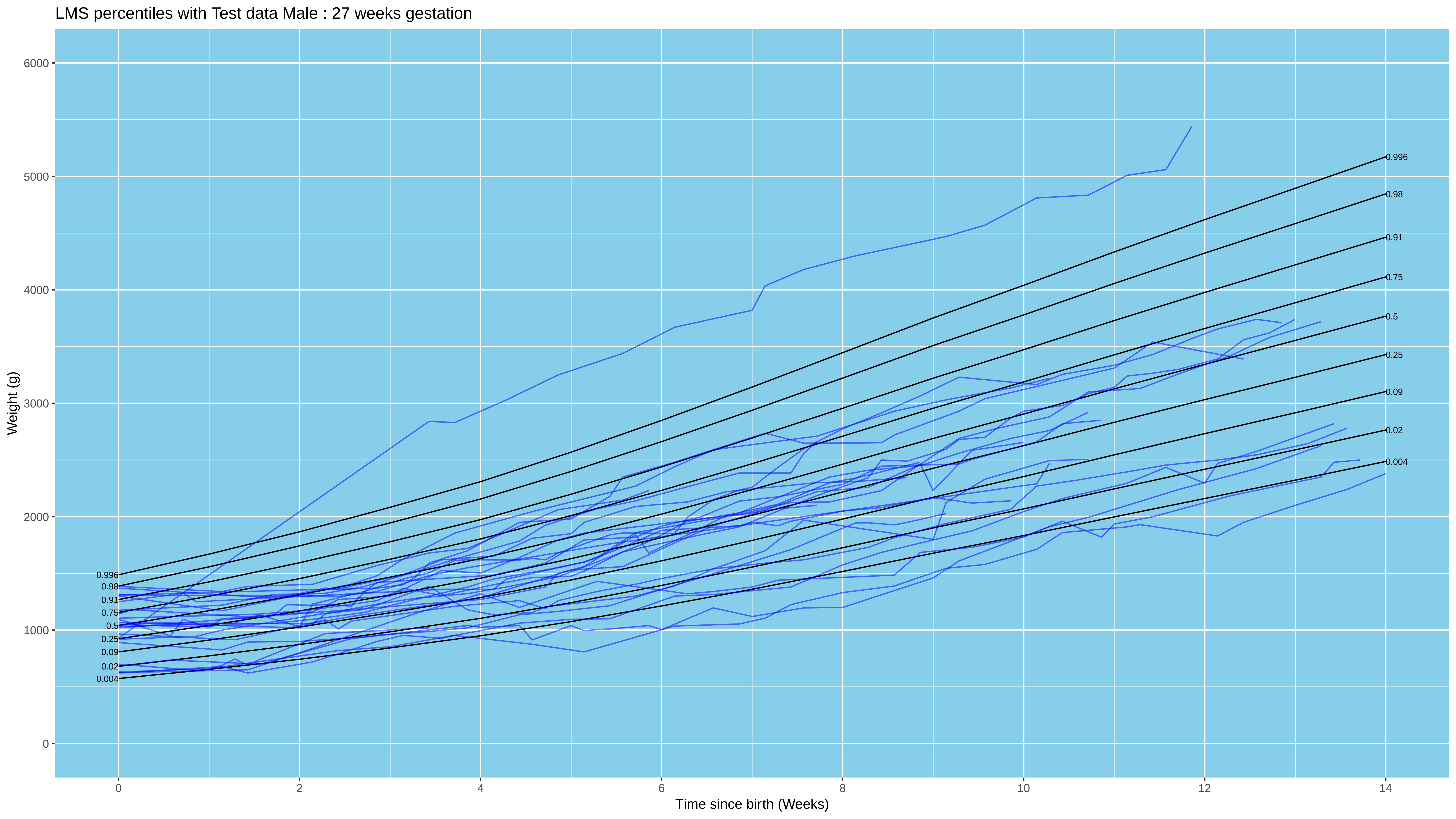
LMS percentiles with Test data Male : 27 weeks gestation
Weight (g)
Time since birth (Weeks)
0
1000
2000
3000
4000
5000
6000
0
2
4
6
8
10
12
14
0.996
0.98
0.91
0.75
0.5
0.25
0.09
0.02
0.004

Predicted percentiles with model data Female : 27 weeks gestation

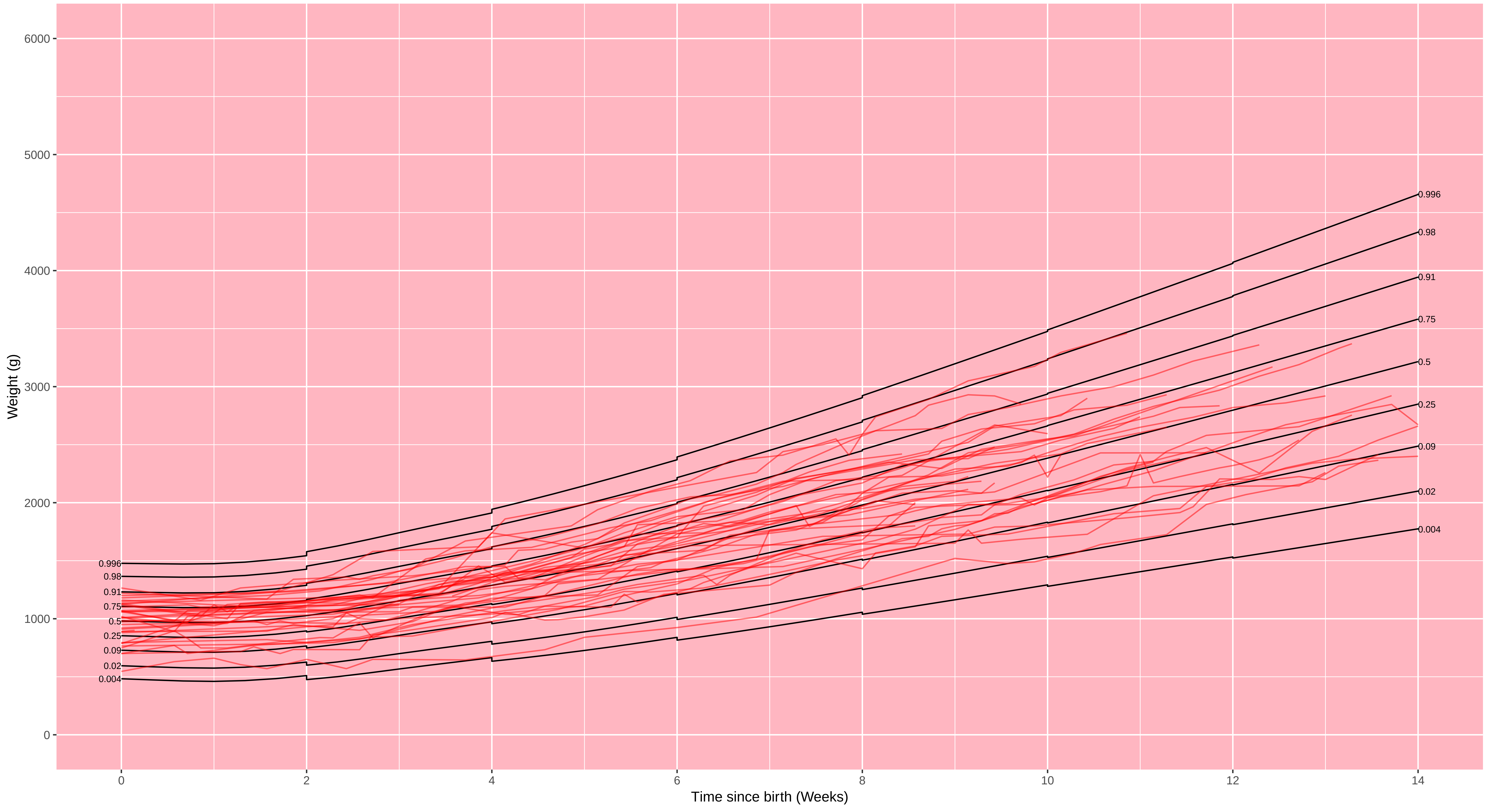

Predicted percentiles Female : 27 weeks gestation

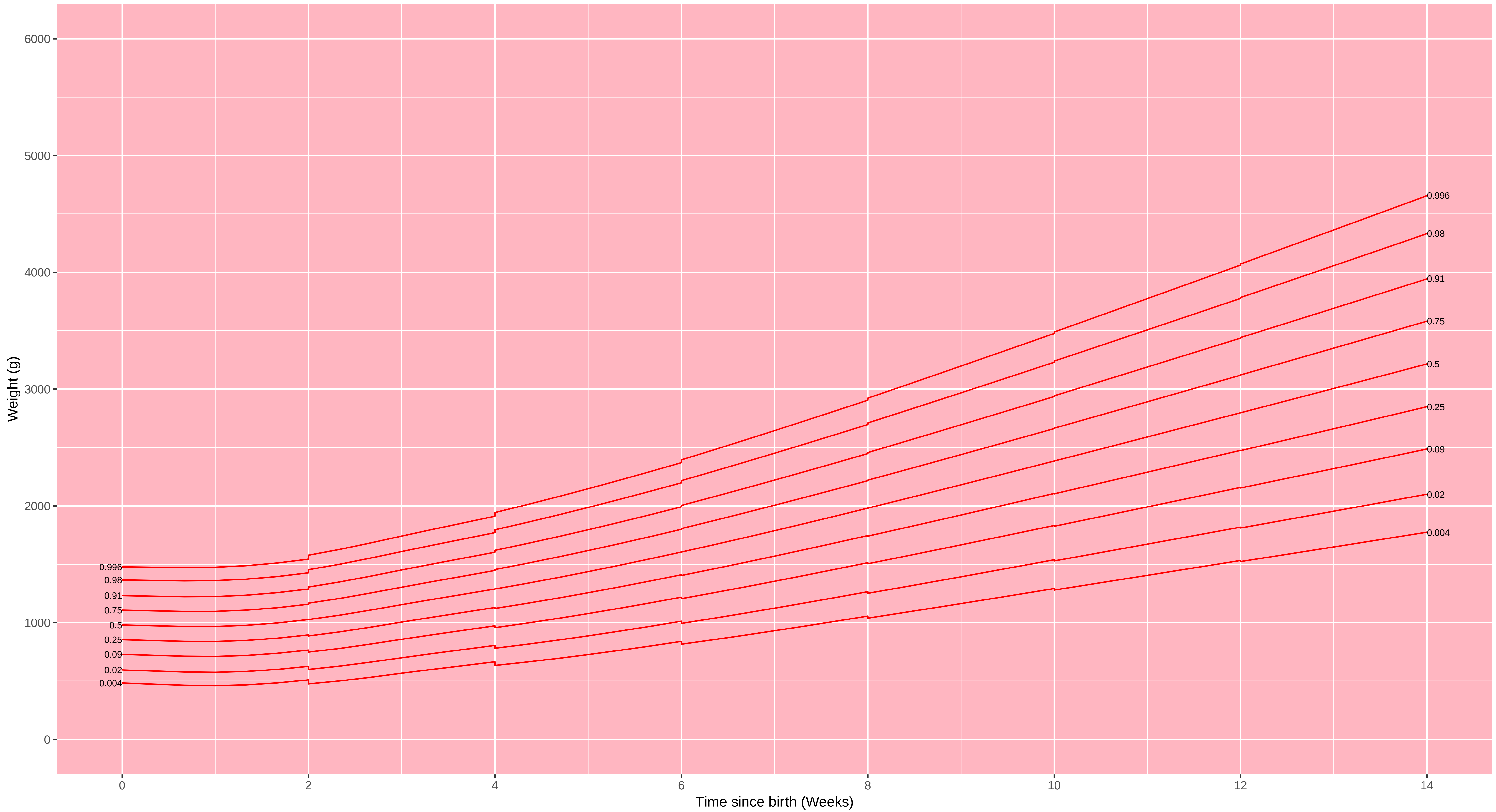

Predicted percentiles with Test data Female : 27 weeks gestation

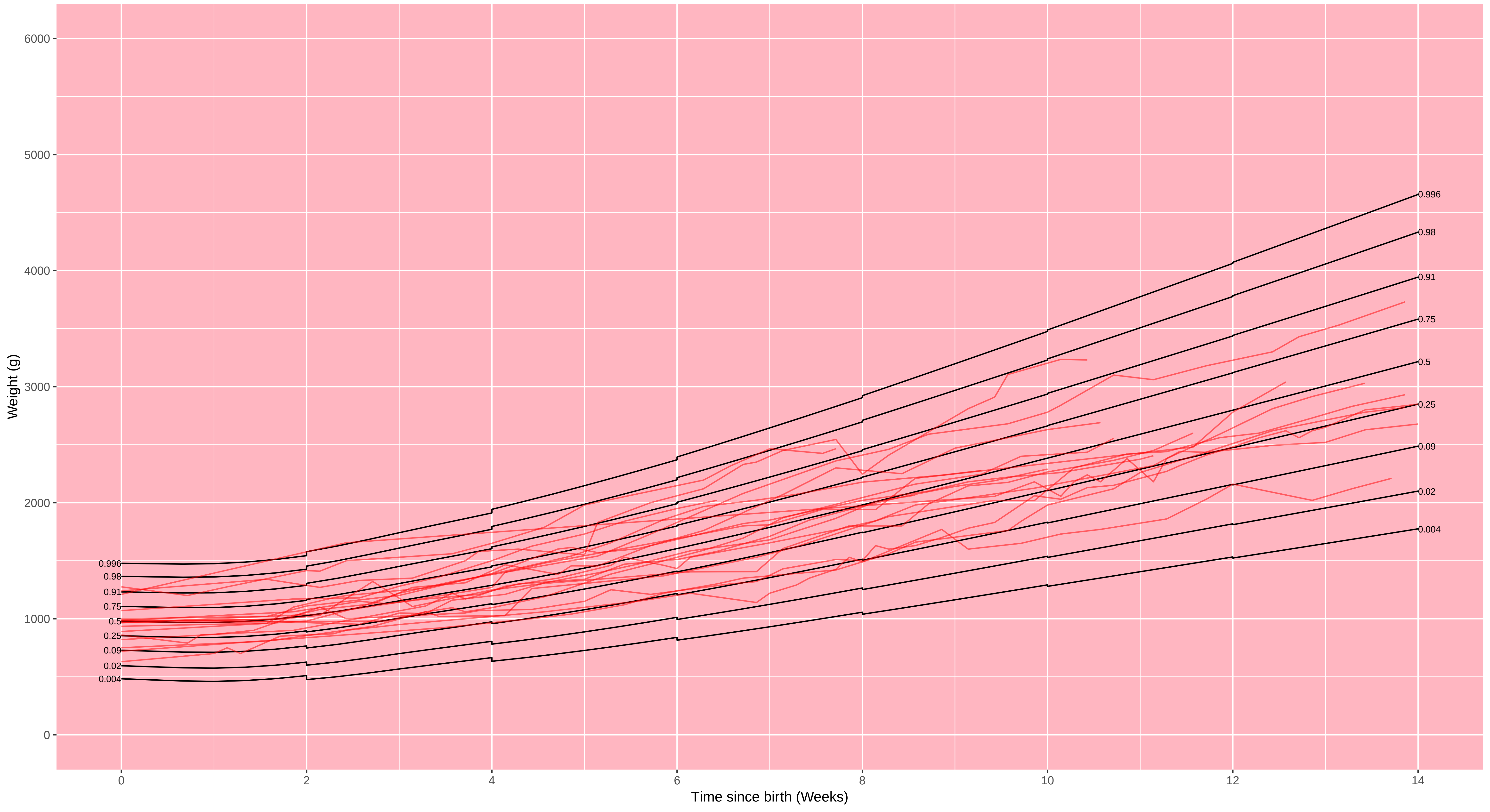

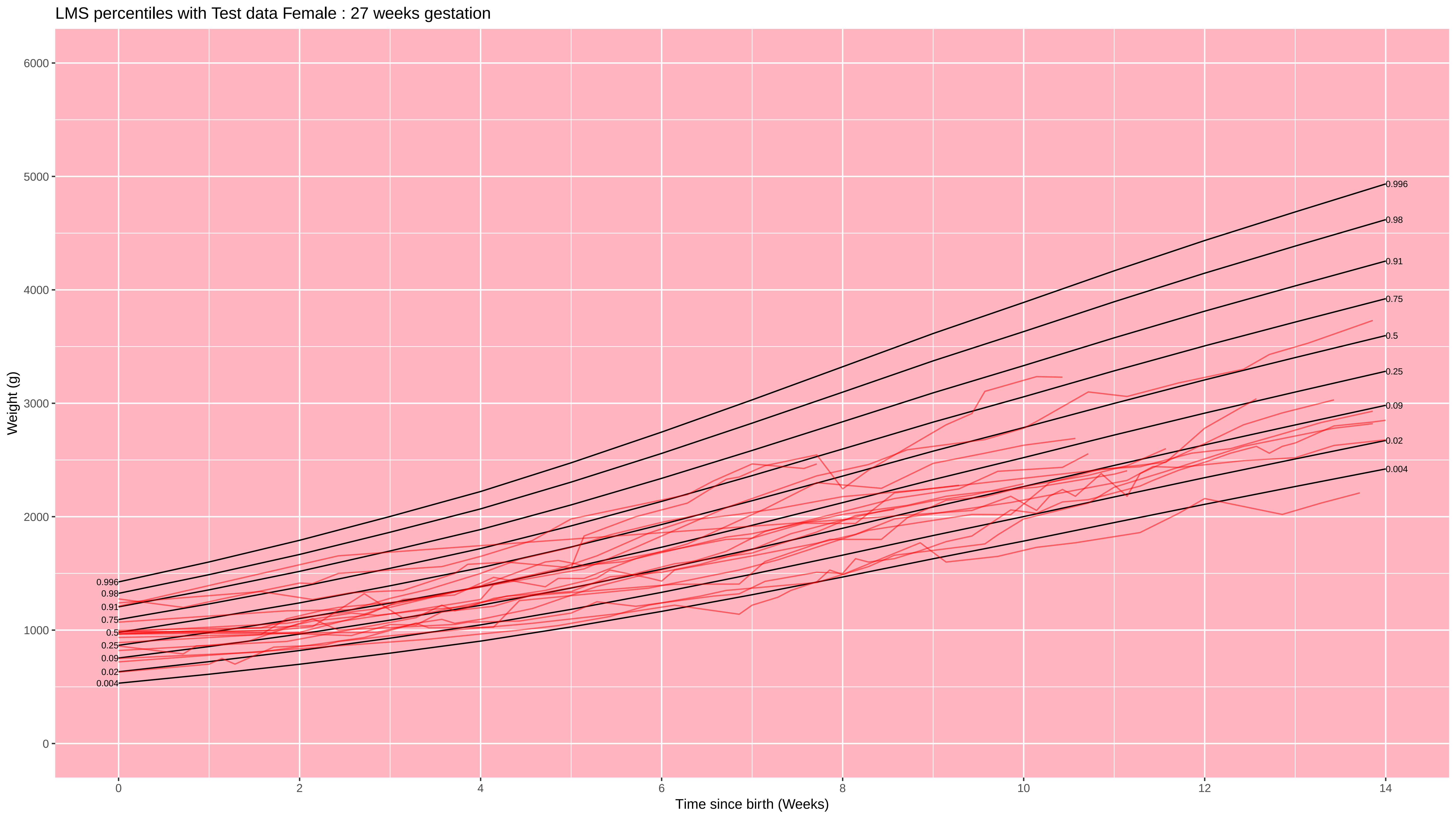

LMS percentiles with Test data Female : 27 weeks gestation
Weight (g)
Time since birth (Weeks)
0
1000
2000
3000
4000
5000
6000
0
2
4
6
8
10
12
14
0.996
0.98
0.91
0.75
0.5
0.25
0.09
0.02
0.004

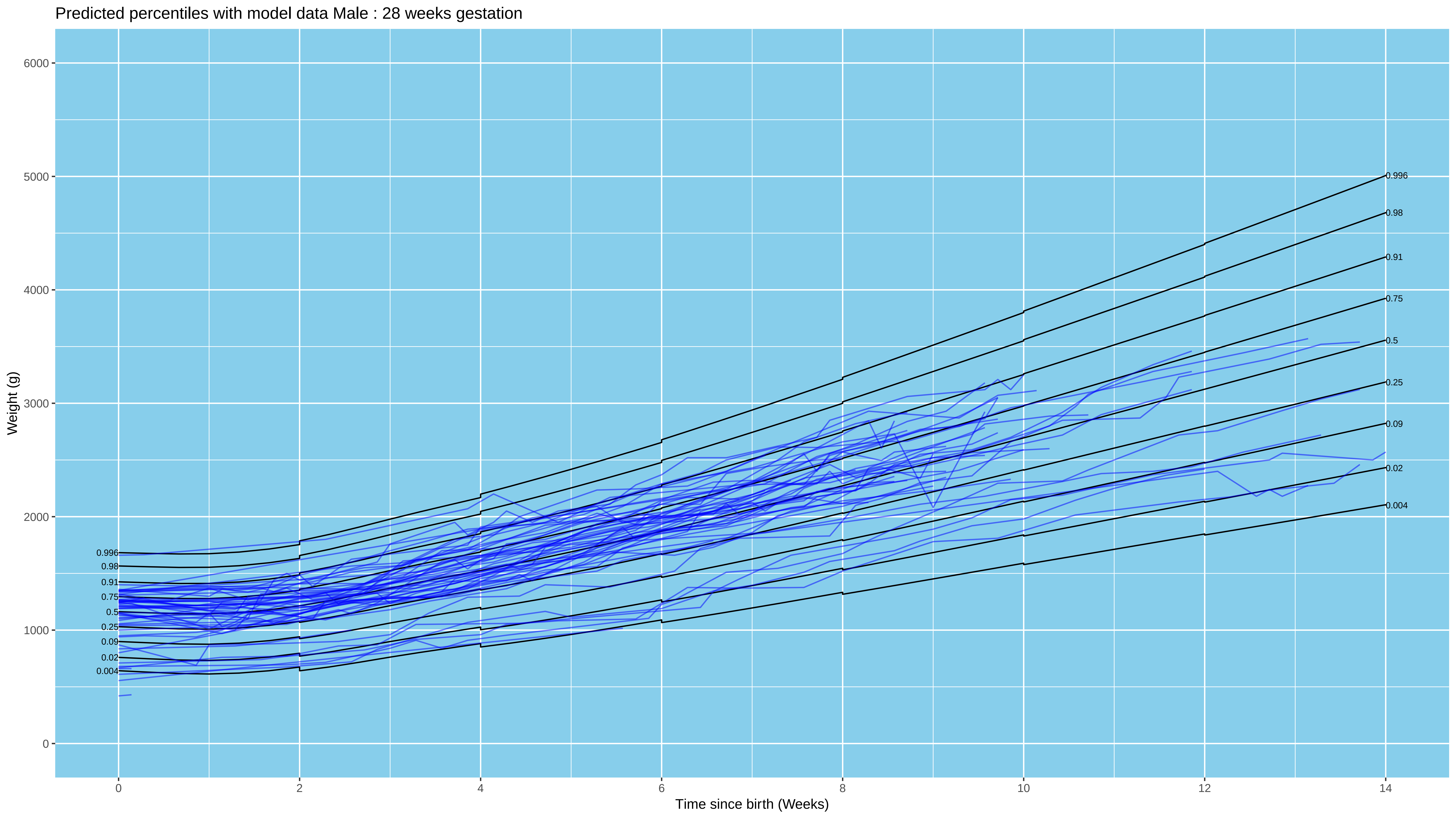

Predicted percentiles with model data Male : 28 weeks gestation
Weight (g)
Time since birth (Weeks)
0
1000
2000
3000
4000
5000
6000
0
2
4
6
8
10
12
14
0.996
0.98
0.91
0.75
0.5
0.25
0.09
0.02
0.004

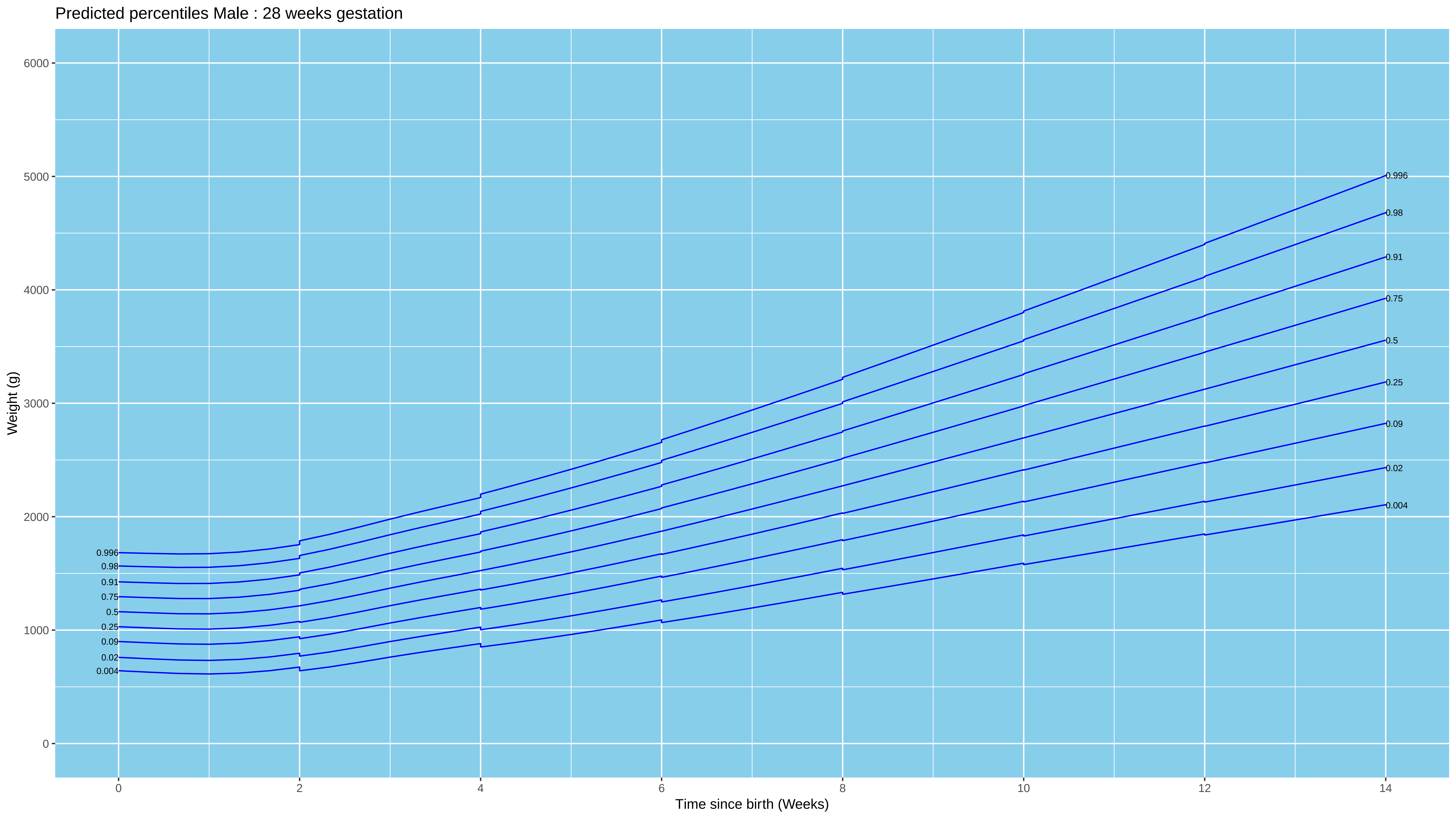

Predicted percentiles Male : 28 weeks gestation
Weight (g)
Time since birth (Weeks)
0
1000
2000
3000
4000
5000
6000
0
2
4
6
8
10
12
14
0.996
0.98
0.91
0.75
0.5
0.25
0.09
0.02
0.004

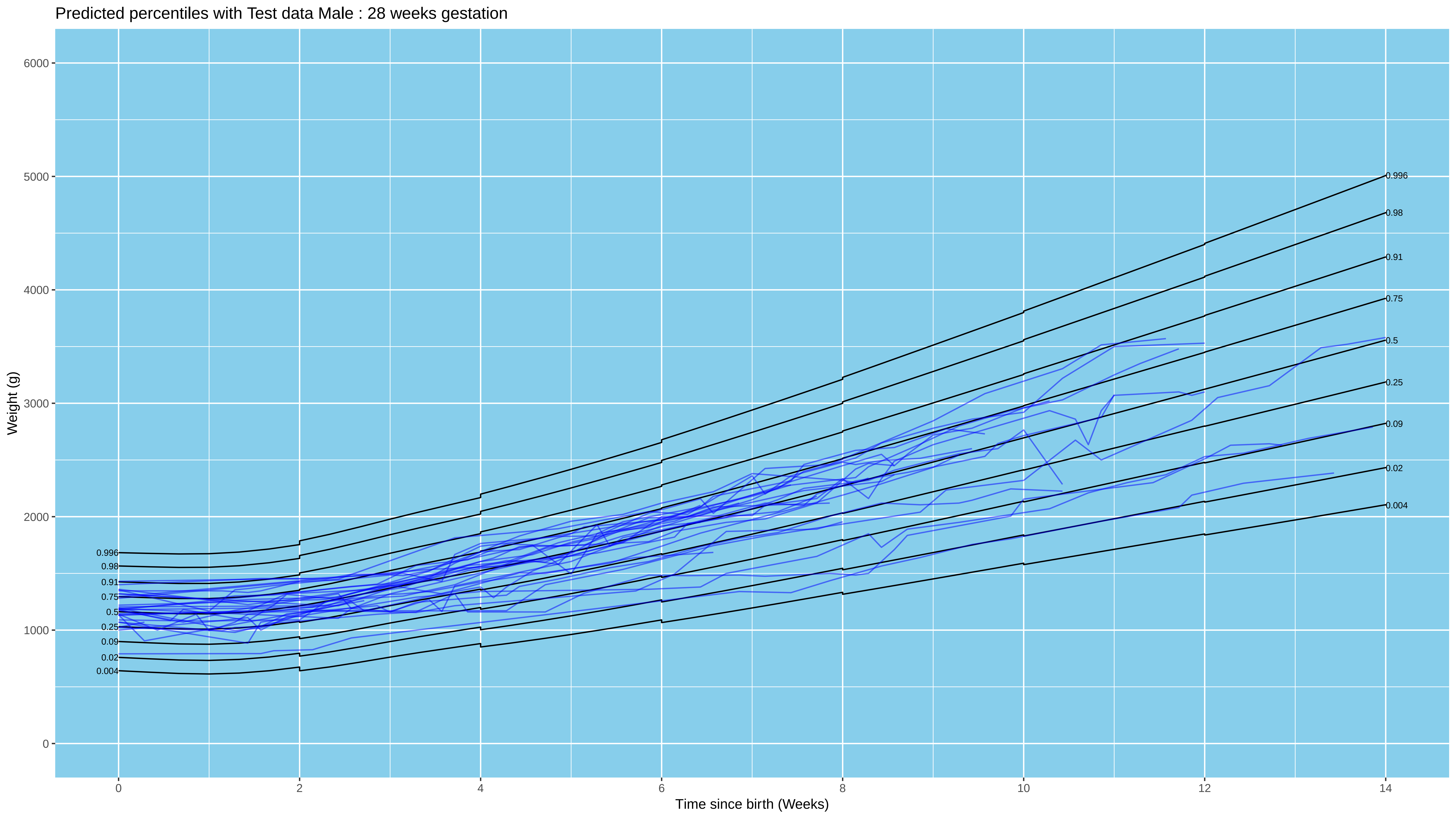

Predicted percentiles with Test data Male : 28 weeks gestation
Weight (g)
Time since birth (Weeks)
0
1000
2000
3000
4000
5000
6000
0
2
4
6
8
10
12
14
0.996
0.98
0.91
0.75
0.5
0.25
0.09
0.02
0.004

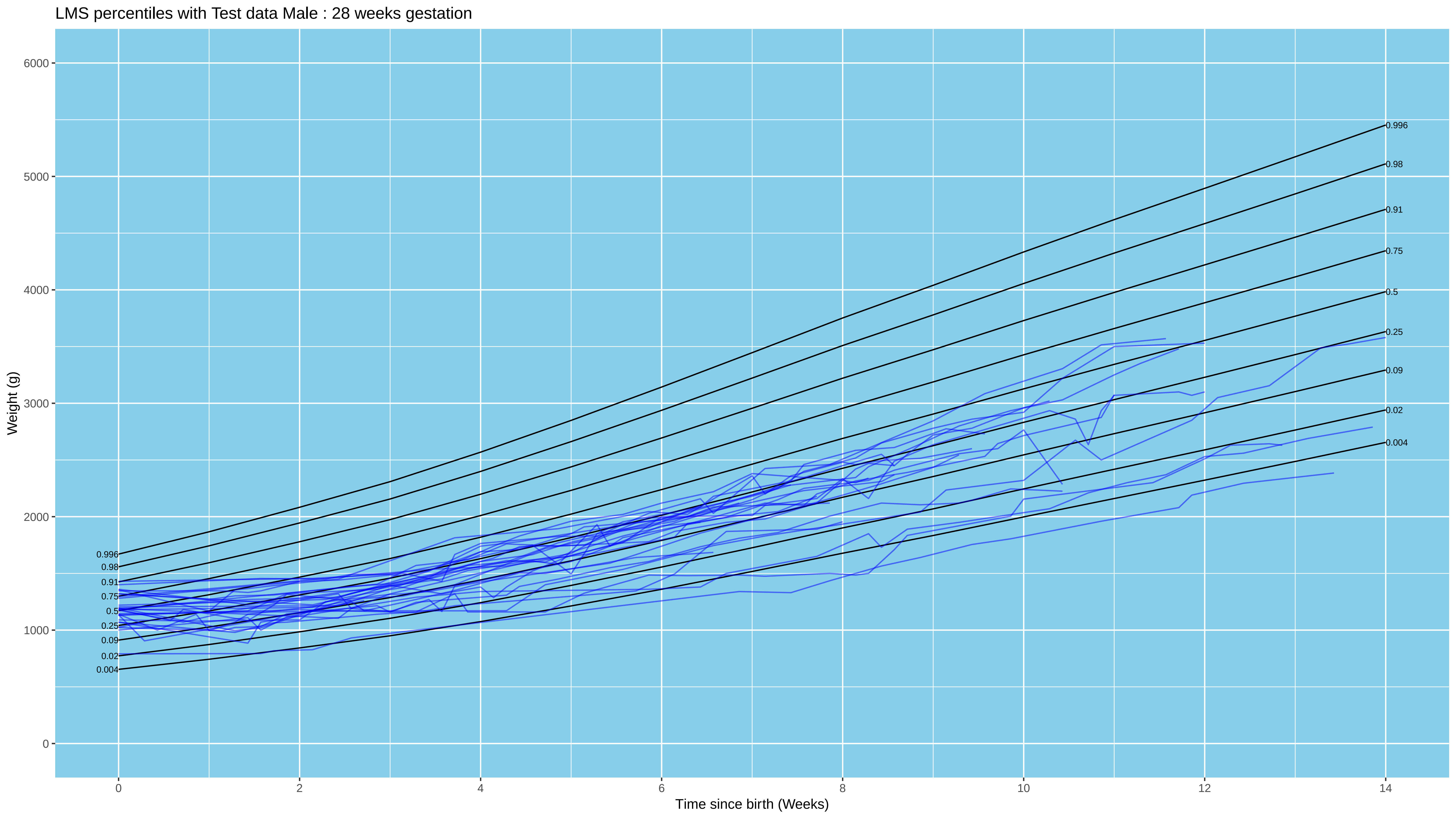
LMS percentiles with Test data Male : 28 weeks gestation
Weight (g)
Time since birth (Weeks)
0.996
0.98
0.91
0.75
0.5
0.25
0.09
0.02
0.004
0
1000
2000
3000
4000
5000
6000
0
2
4
6
8
10
12
14

Predicted percentiles with model data Female : 28 weeks gestation

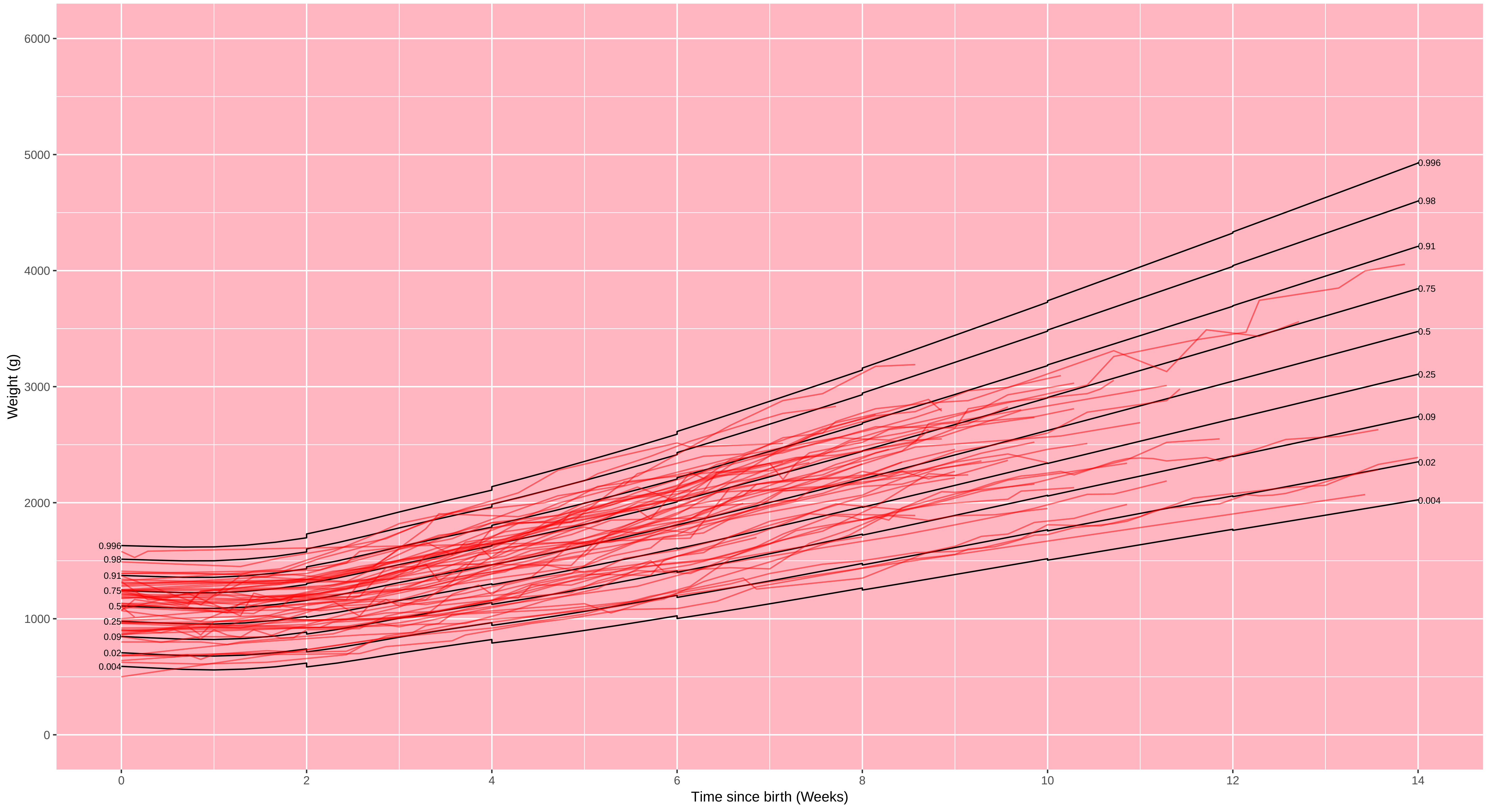

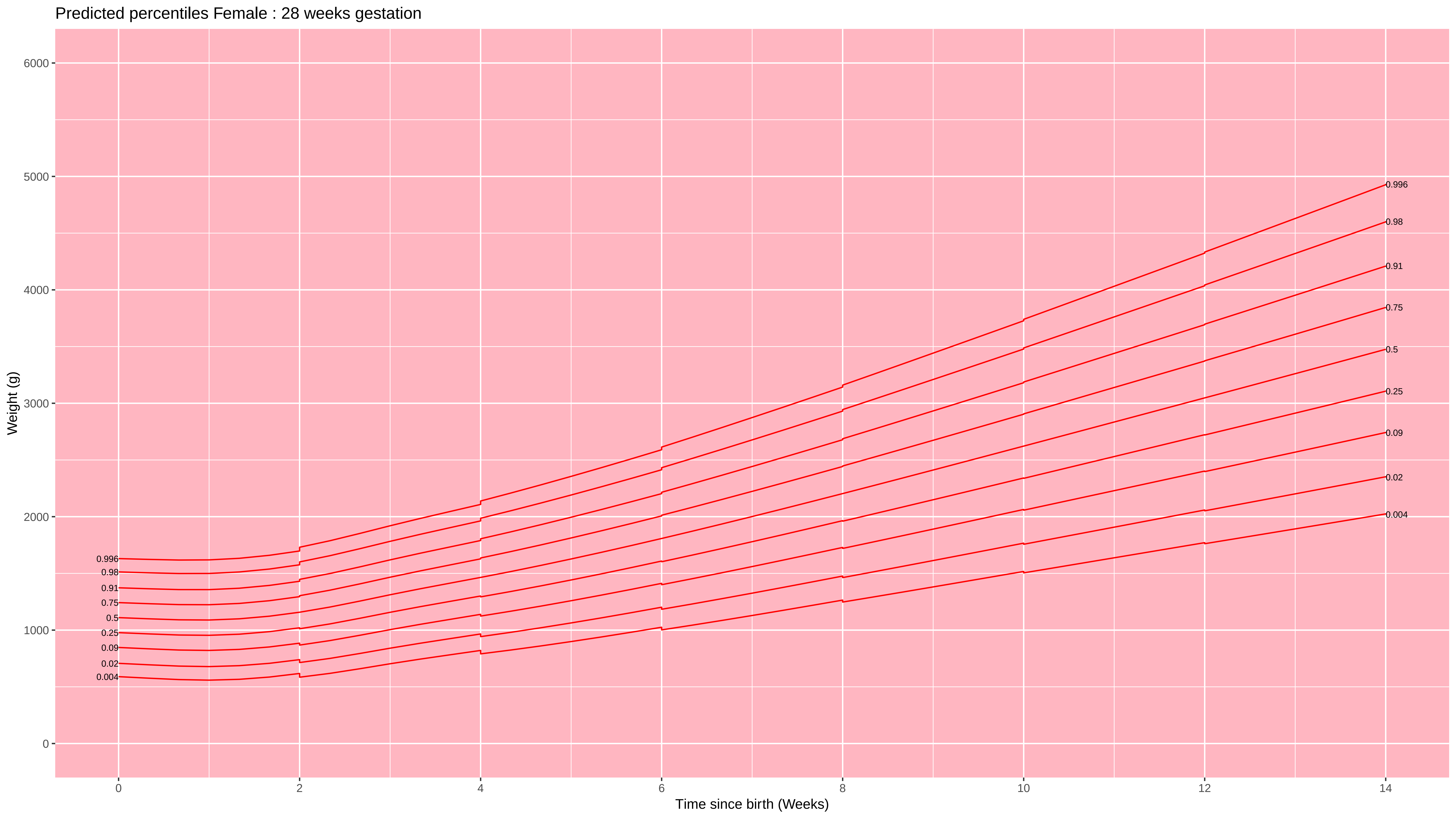
Predicted percentiles Female : 28 weeks gestation
Weight (g)
6000
5000
4000
3000
2000
1000
0
Time since birth (Weeks)
0
2
4
6
8
10
12
14
0.996
0.98
0.91
0.75
0.5
0.25
0.09
0.02
0.004

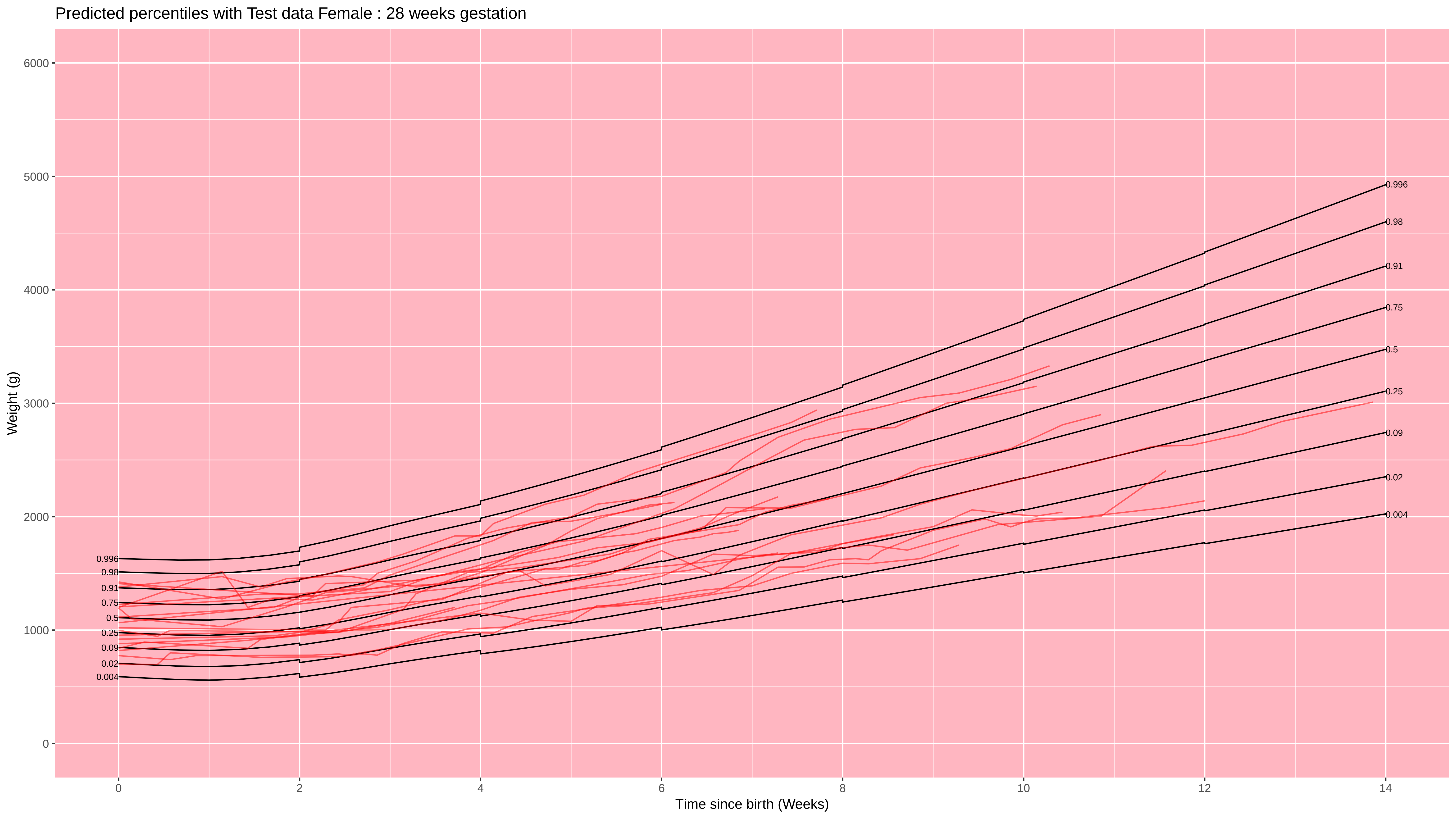
Predicted percentiles with Test data Female : 28 weeks gestation
Weight (g)
Time since birth (Weeks)
0
1000
2000
3000
4000
5000
6000
0
2
4
6
8
10
12
14
0.996
0.98
0.91
0.75
0.5
0.25
0.09
0.02
0.004

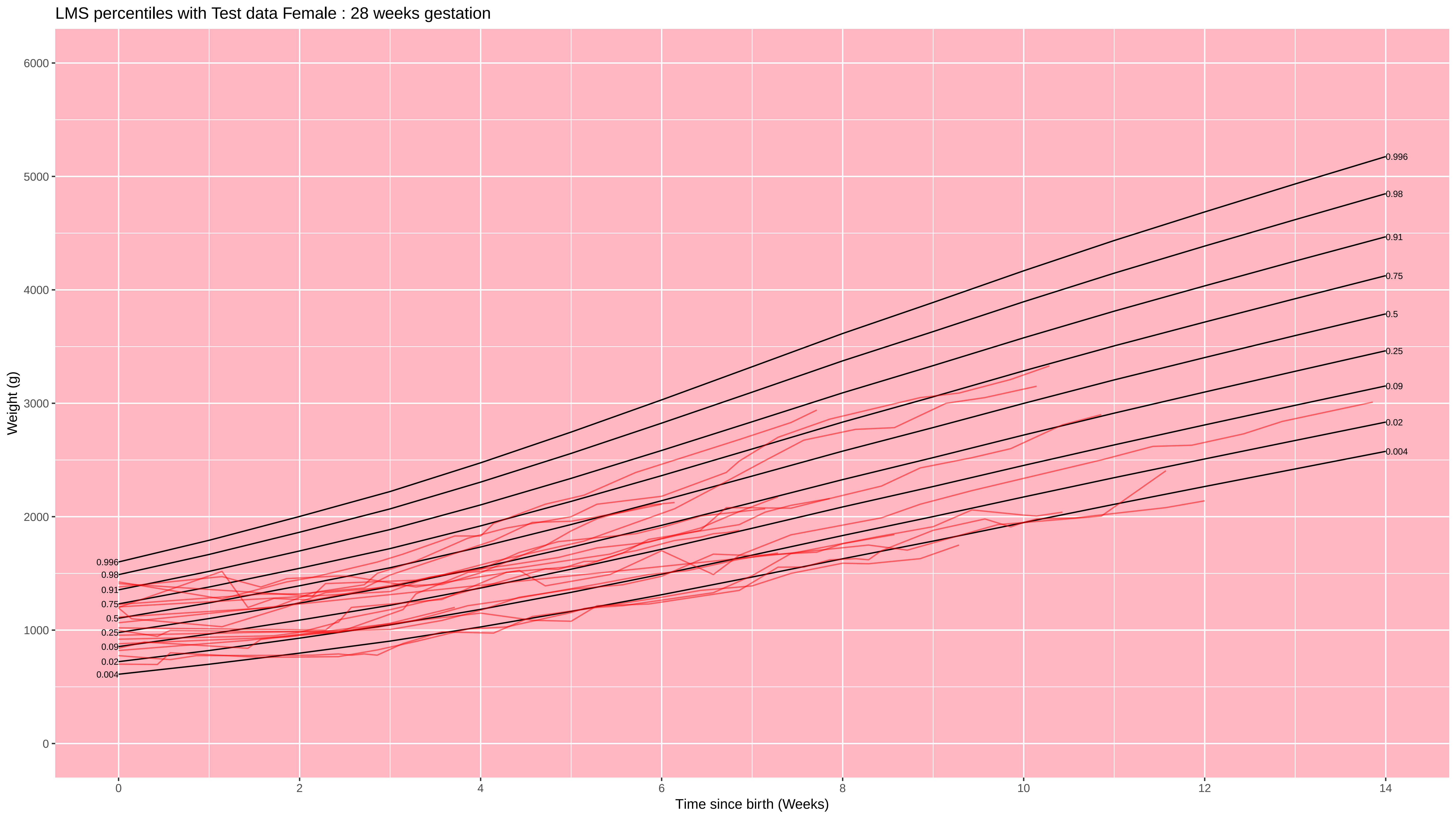

LMS percentiles with Test data Female : 28 weeks gestation
Weight (g)
Time since birth (Weeks)
0
1000
2000
3000
4000
5000
6000
0
2
4
6
8
10
12
14
0.996
0.98
0.91
0.75
0.5
0.25
0.09
0.02
0.004

Predicted percentiles with model data Male : 29 weeks gestation

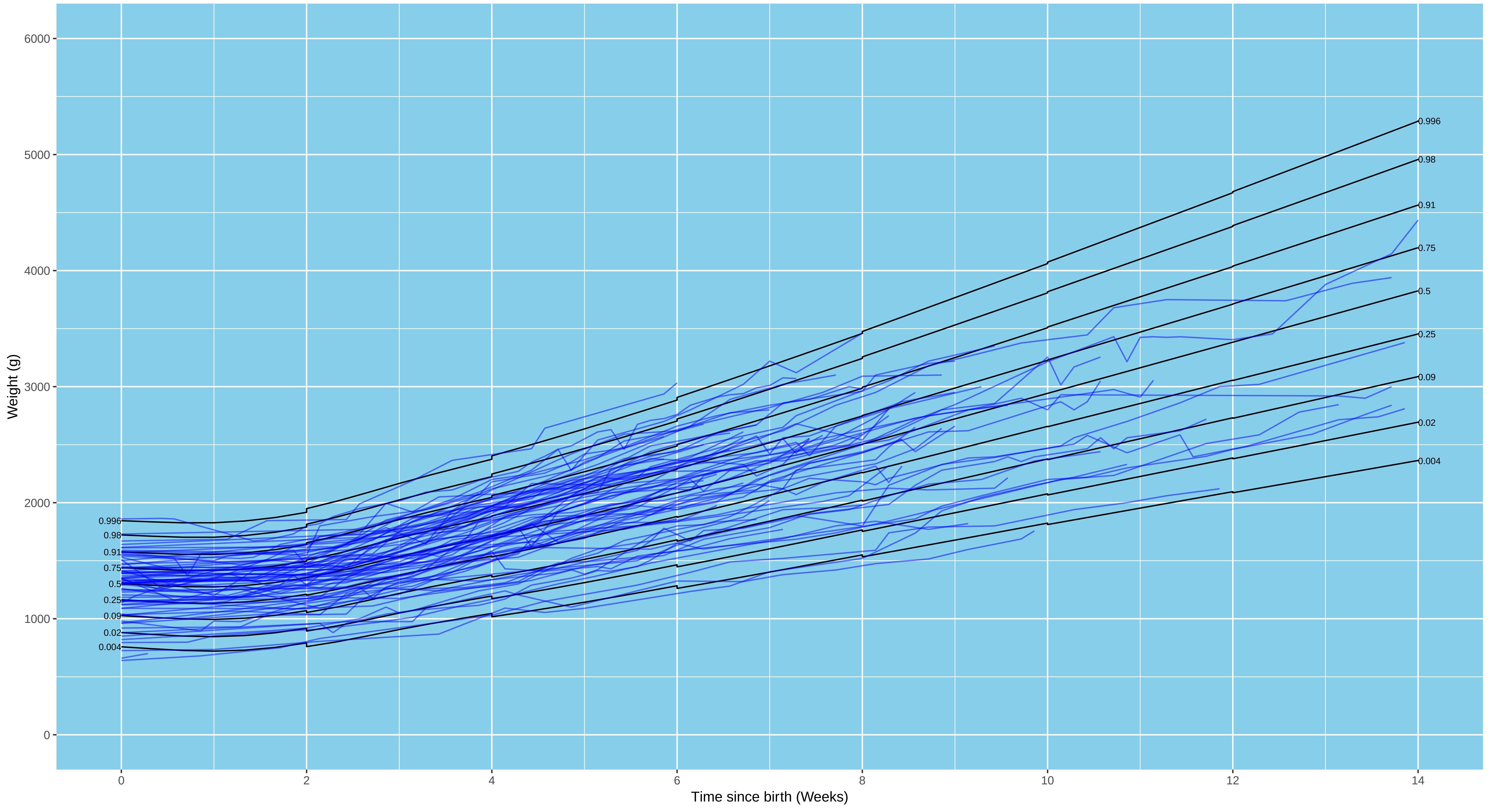

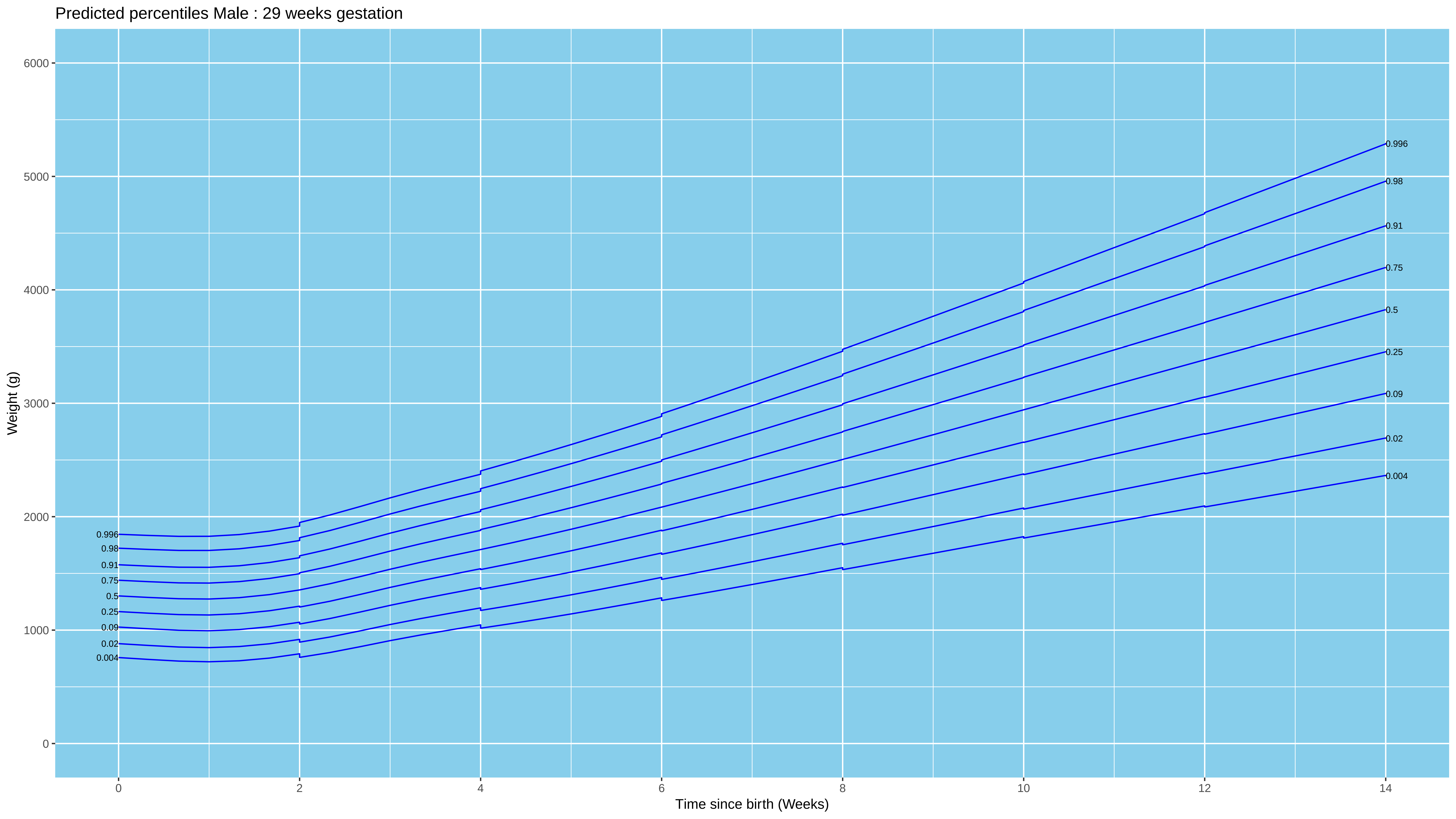
Predicted percentiles Male : 29 weeks gestation
Weight (g)
Time since birth (Weeks)
0
1000
2000
3000
4000
5000
6000
0
2
4
6
8
10
12
14
0.996
0.98
0.91
0.75
0.5
0.25
0.09
0.02
0.004

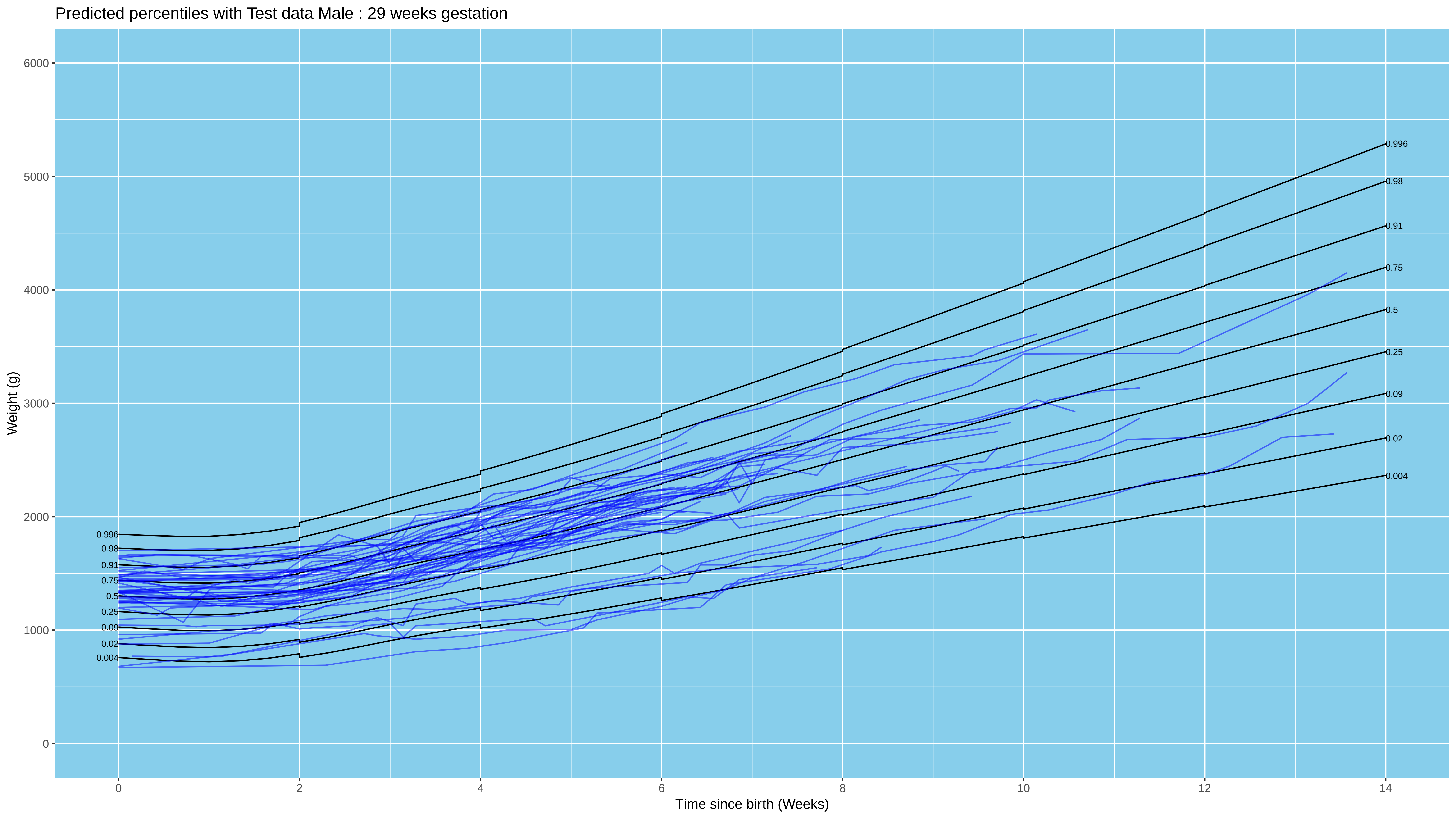

Predicted percentiles with Test data Male : 29 weeks gestation
Weight (g)
Time since birth (Weeks)
0
1000
2000
3000
4000
5000
6000
0
2
4
6
8
10
12
14
0.996
0.98
0.91
0.75
0.5
0.25
0.09
0.02
0.004

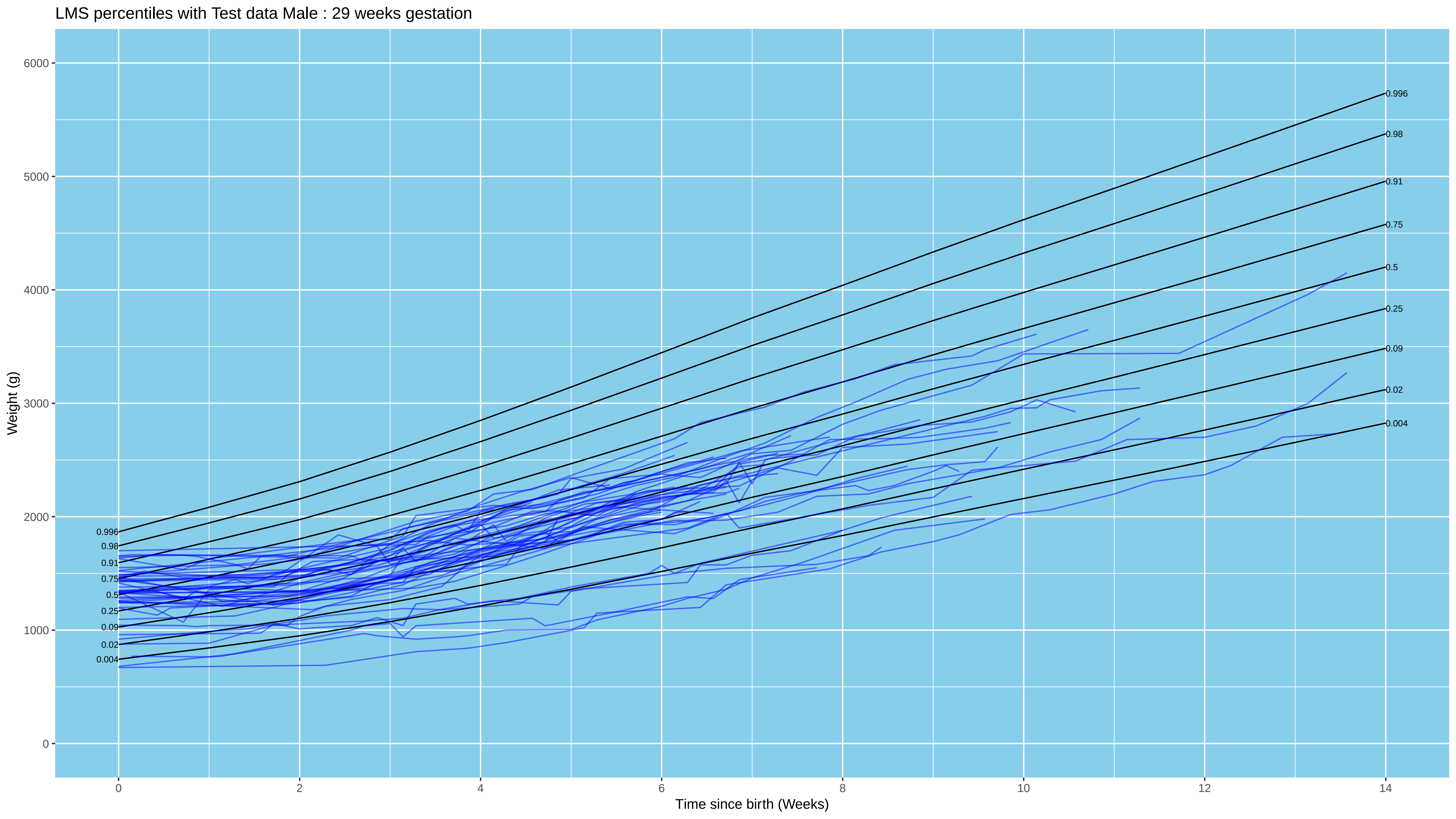

LMS percentiles with Test data Male : 29 weeks gestation
Weight (g)
Time since birth (Weeks)
0
1000
2000
3000
4000
5000
6000
0
2
4
6
8
10
12
14
0.996
0.98
0.91
0.75
0.5
0.25
0.09
0.02
0.004

Predicted percentiles with model data Female : 29 weeks gestation

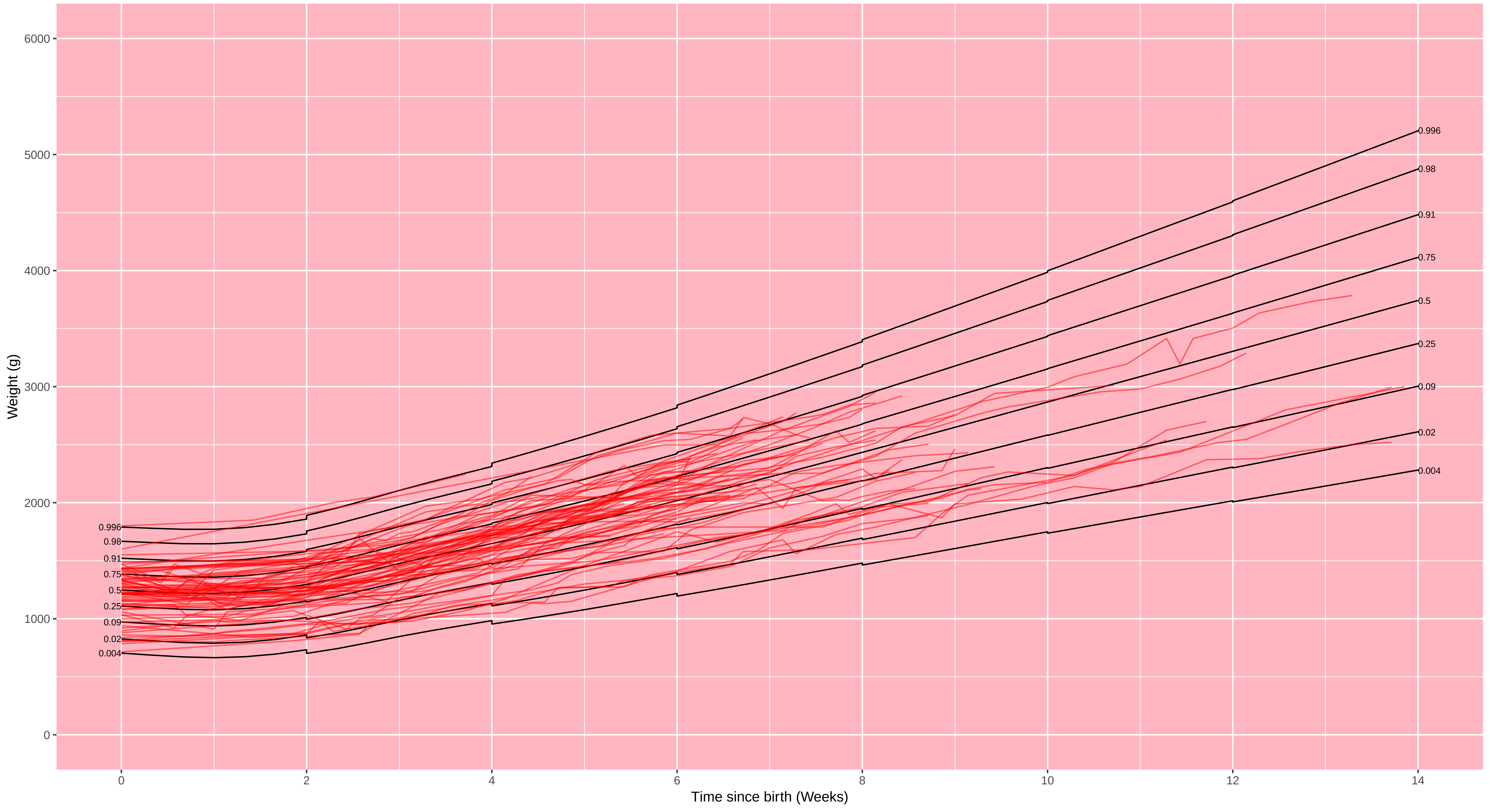

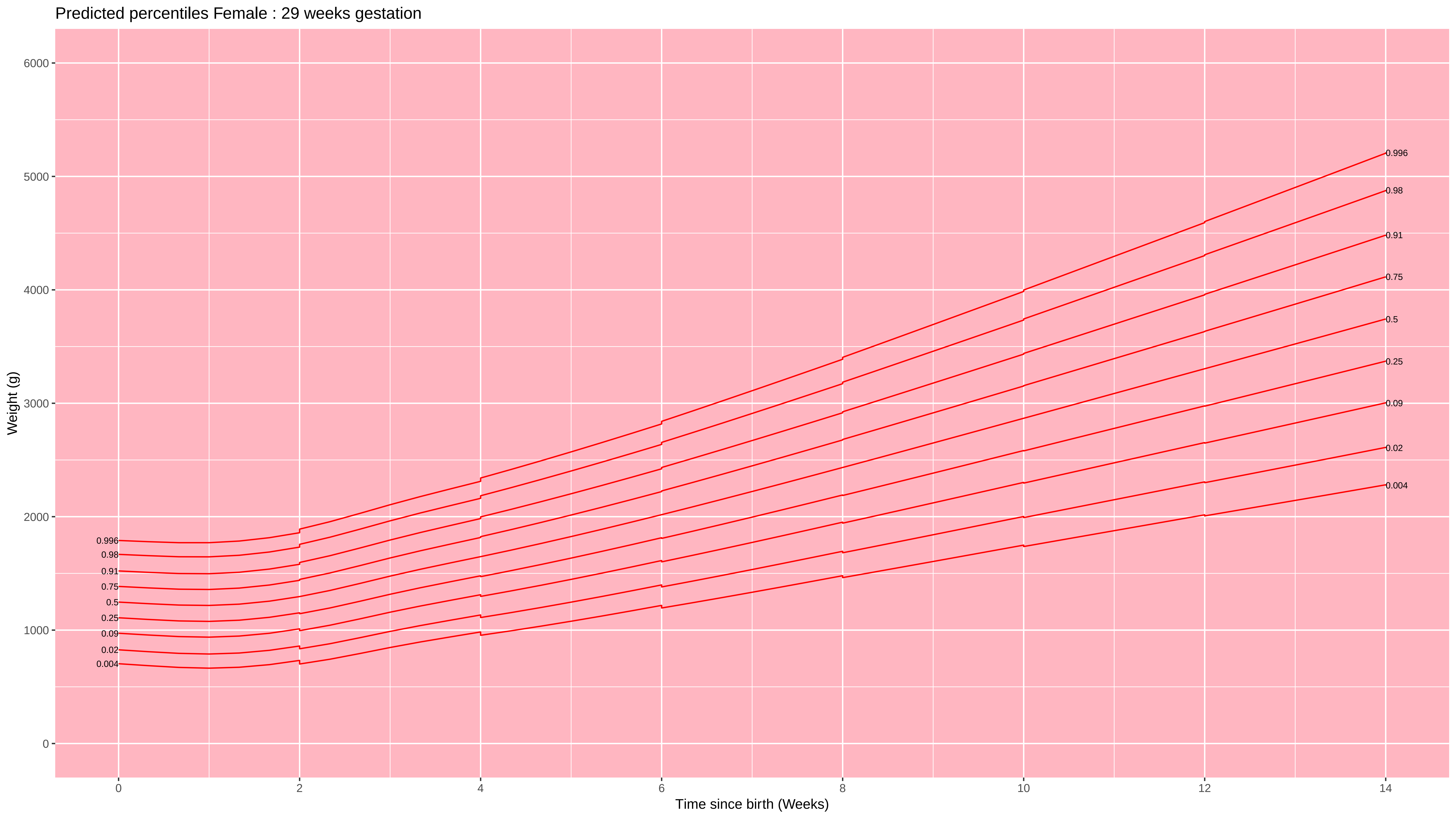
Predicted percentiles Female : 29 weeks gestation
Weight (g)
Time since birth (Weeks)
0
1000
2000
3000
4000
5000
6000
0
2
4
6
8
10
12
14
0.996
0.98
0.91
0.75
0.5
0.25
0.09
0.02
0.004

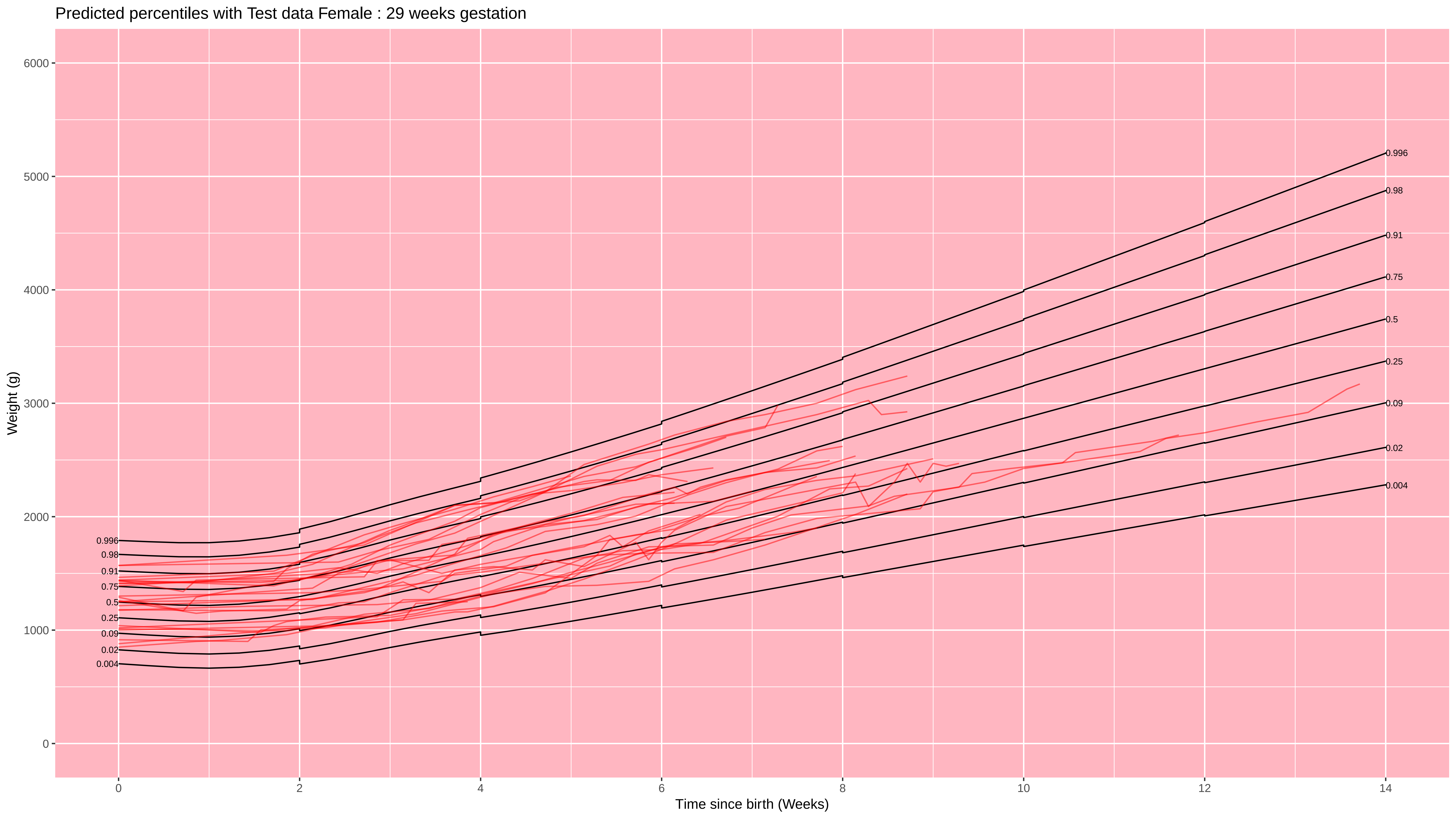
Predicted percentiles with Test data Female : 29 weeks gestation
Weight (g)
Time since birth (Weeks)
0
1000
2000
3000
4000
5000
6000
0
2
4
6
8
10
12
14
0.996
0.98
0.91
0.75
0.5
0.25
0.09
0.02
0.004

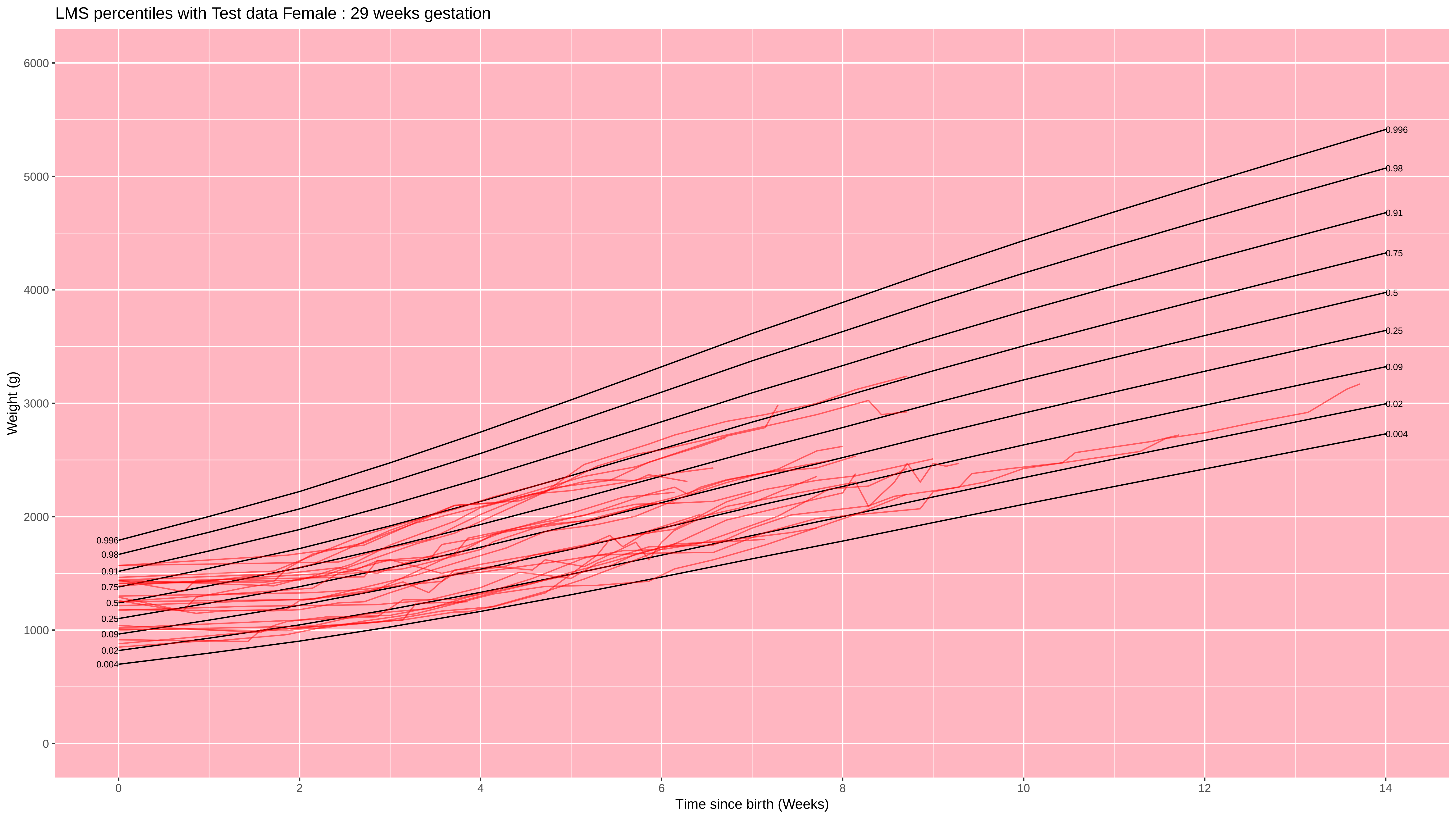
LMS percentiles with Test data Female : 29 weeks gestation
Weight (g)
6000
5000
4000
3000
2000
1000
0
0.996
0.98
0.91
0.75
0.5
0.25
0.09
0.02
0.004
0
2
4
6
8
10
12
14
Time since birth (Weeks)

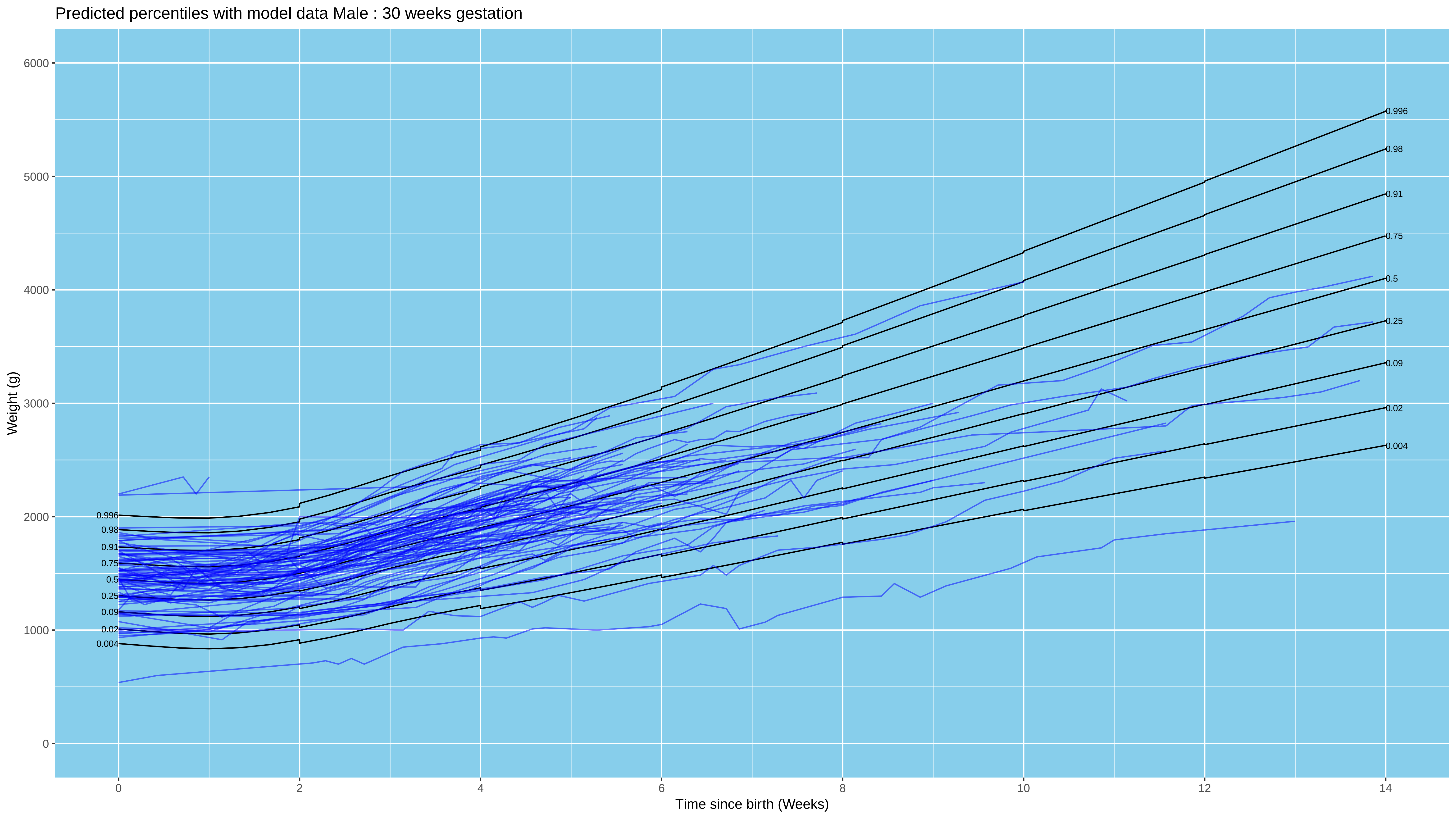
Predicted percentiles with model data Male : 30 weeks gestation
Weight (g)
Time since birth (Weeks)
0
1000
2000
3000
4000
5000
6000
0
2
4
6
8
10
12
14
0.996
0.98
0.91
0.75
0.5
0.25
0.09
0.02
0.004

Predicted percentiles Male : 30 weeks gestation

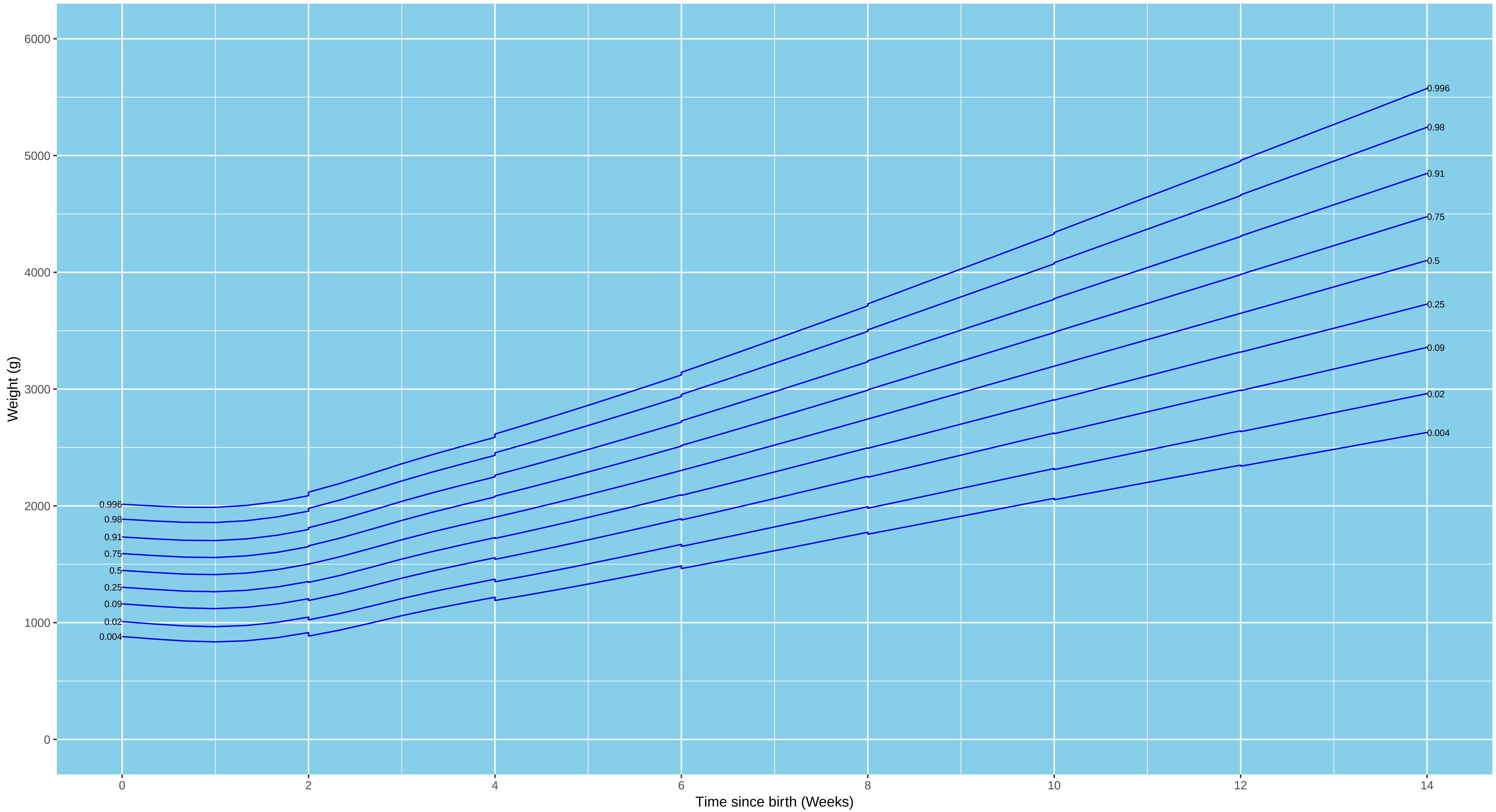

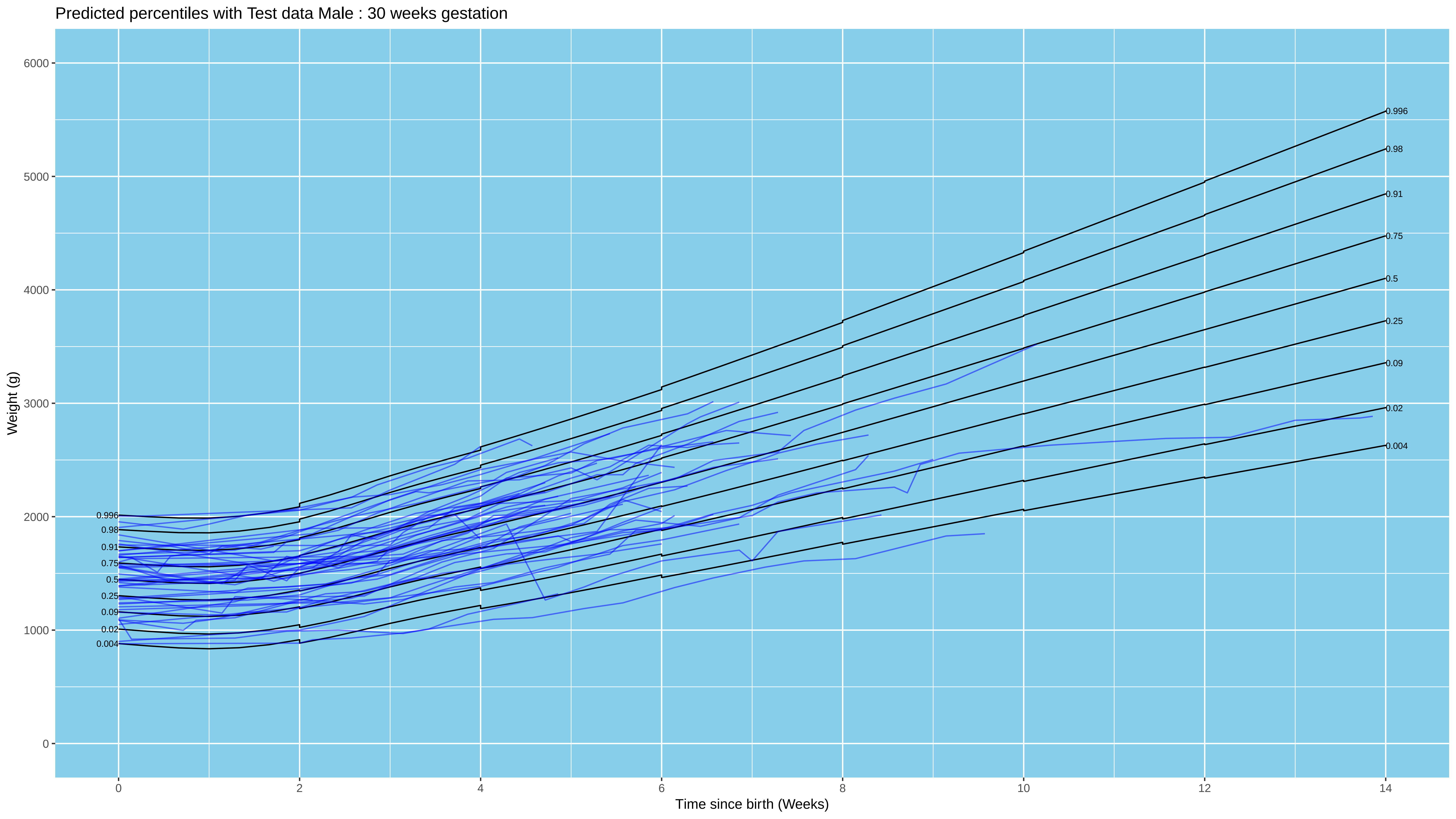

Predicted percentiles with Test data Male : 30 weeks gestation
Weight (g)
Time since birth (Weeks)
0
1000
2000
3000
4000
5000
6000
0
2
4
6
8
10
12
14
0.996
0.98
0.91
0.75
0.5
0.25
0.09
0.02
0.004

LMS percentiles with Test data Male : 30 weeks gestation

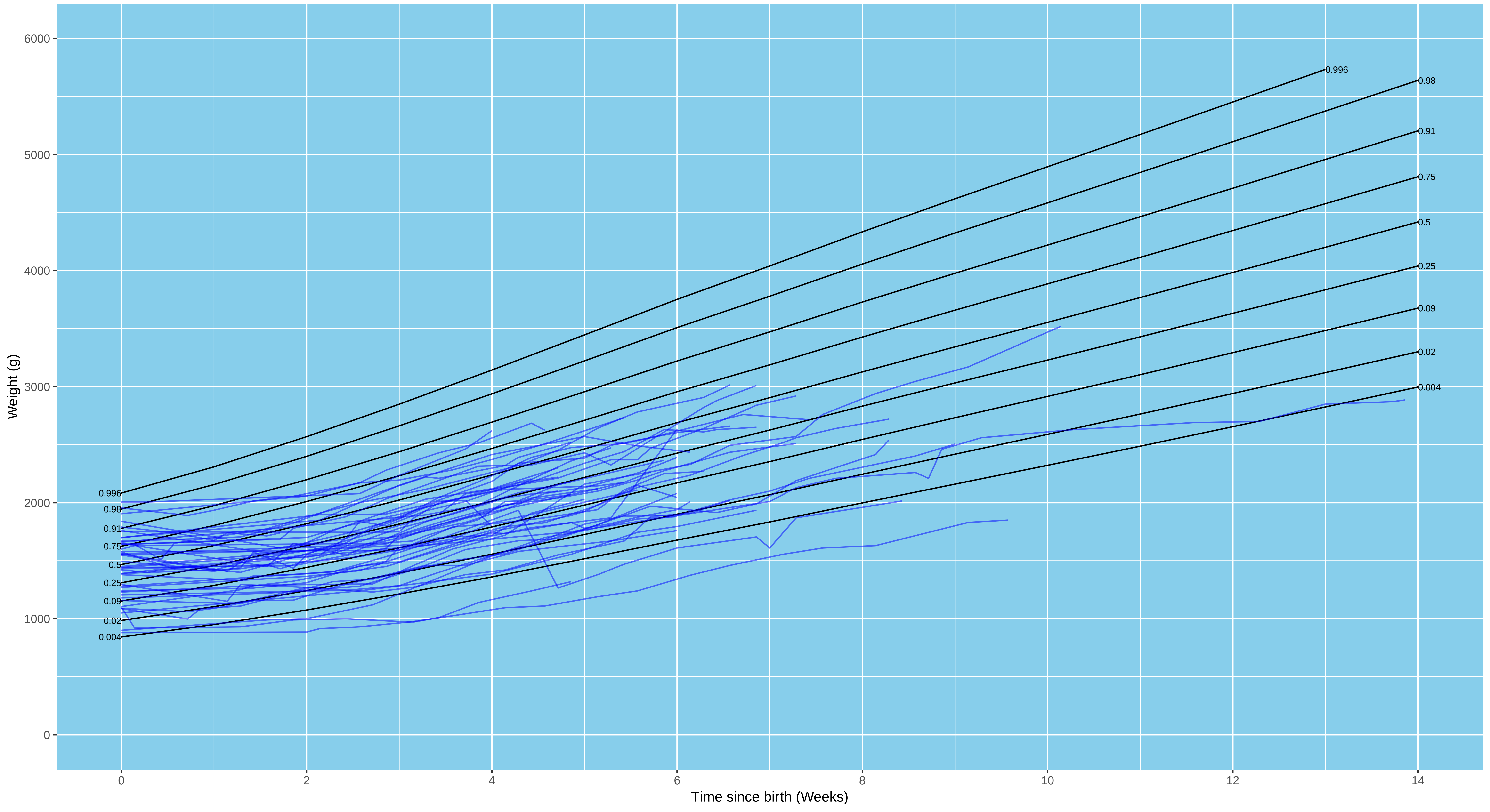

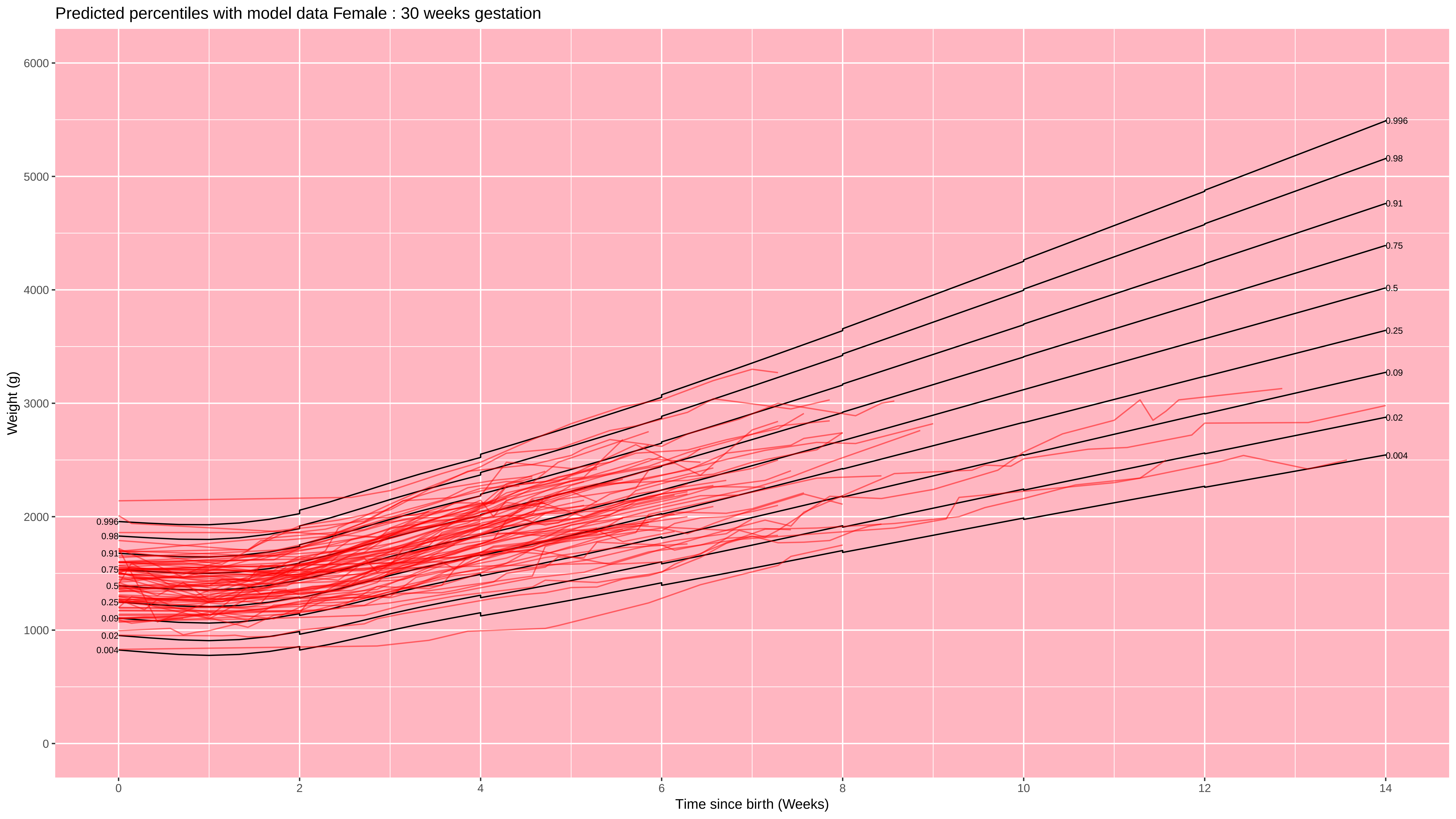

Predicted percentiles with model data Female : 30 weeks gestation
Weight (g)
Time since birth (Weeks)
0
1000
2000
3000
4000
5000
6000
0
2
4
6
8
10
12
14
0.996
0.98
0.91
0.75
0.5
0.25
0.09
0.02
0.004

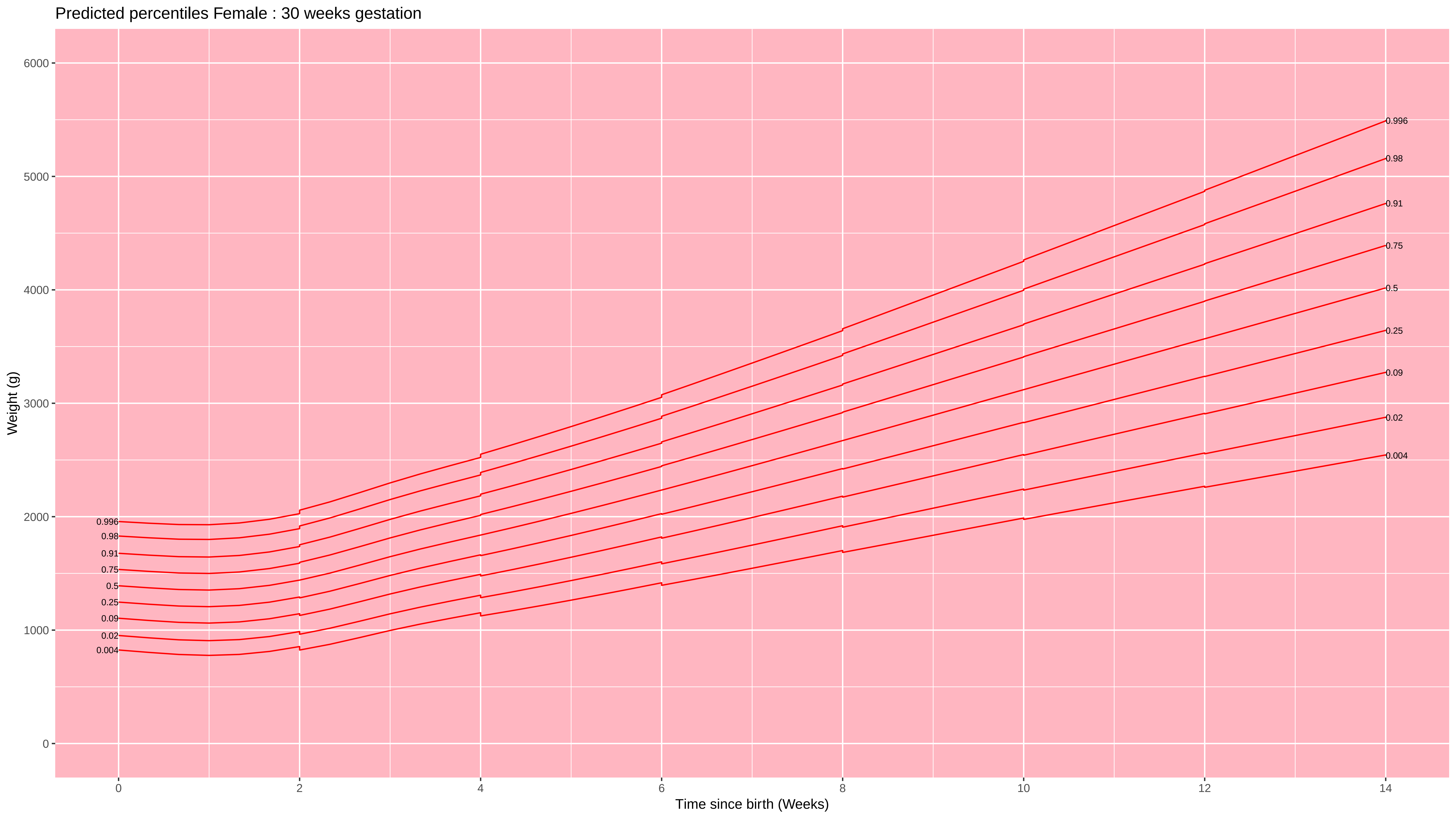
Predicted percentiles Female : 30 weeks gestation
Weight (g)
Time since birth (Weeks)
0
1000
2000
3000
4000
5000
6000
0
2
4
6
8
10
12
14
0.996
0.98
0.91
0.75
0.5
0.25
0.09
0.02
0.004
0.996
0.98
0.91
0.75
0.5
0.25
0.09
0.02
0.004

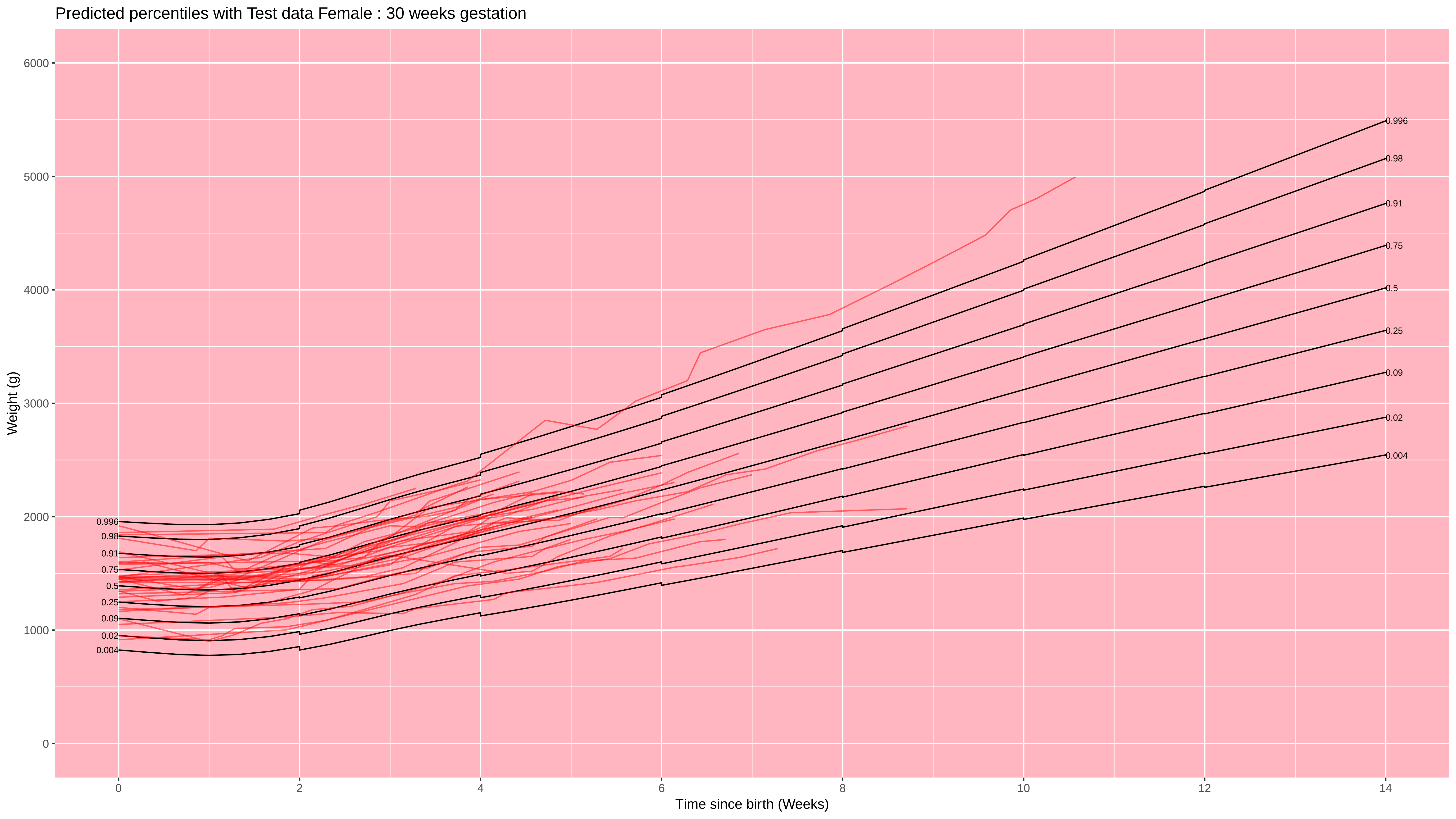

Predicted percentiles with Test data Female : 30 weeks gestation
Weight (g)
Time since birth (Weeks)
0
1000
2000
3000
4000
5000
6000
0
2
4
6
8
10
12
14
0.996
0.98
0.91
0.75
0.5
0.25
0.09
0.02
0.004

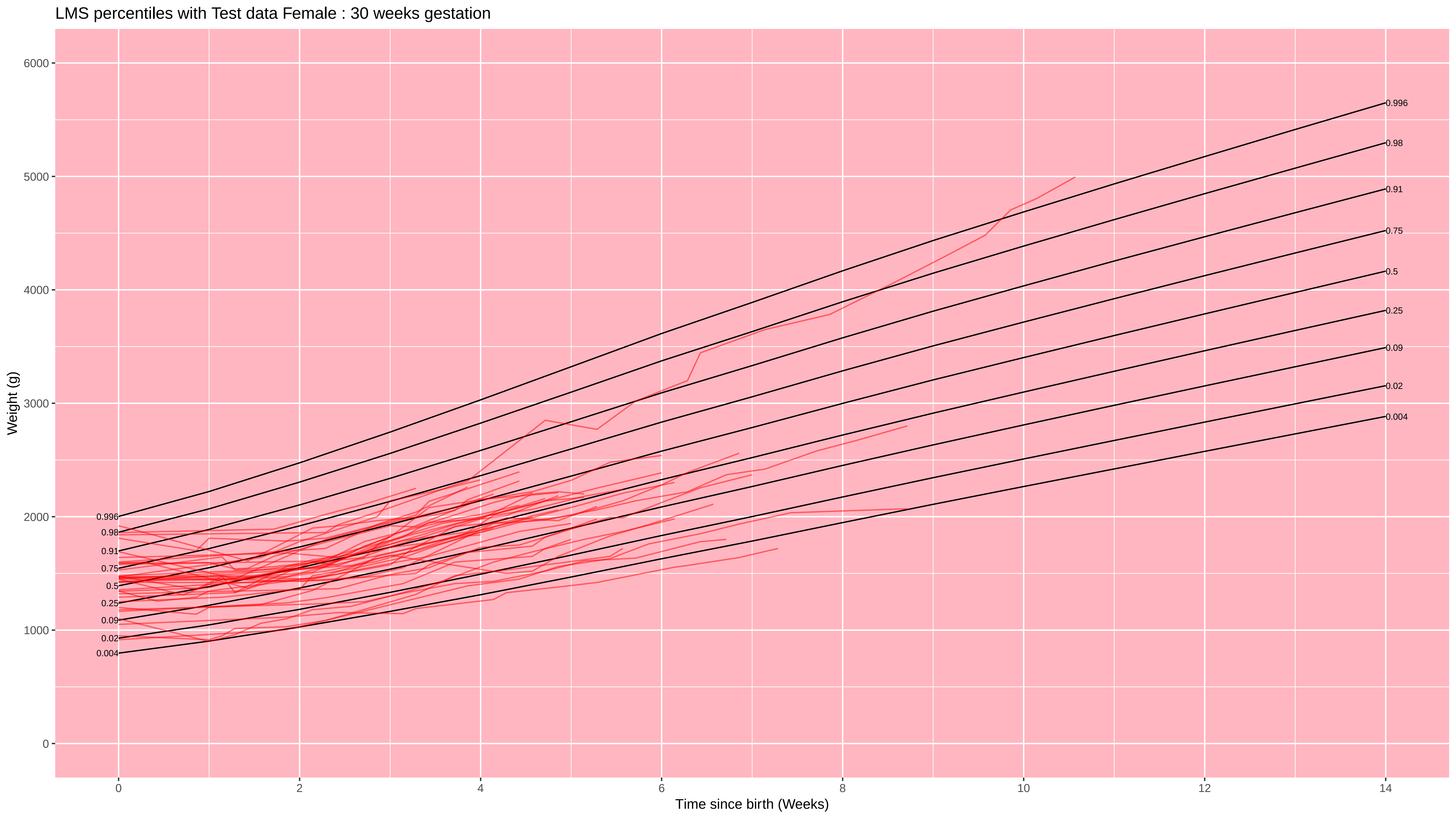

LMS percentiles with Test data Female : 30 weeks gestation
Weight (g)
Time since birth (Weeks)
0
1000
2000
3000
4000
5000
6000
0
2
4
6
8
10
12
14
0.996
0.98
0.91
0.75
0.5
0.25
0.09
0.02
0.004

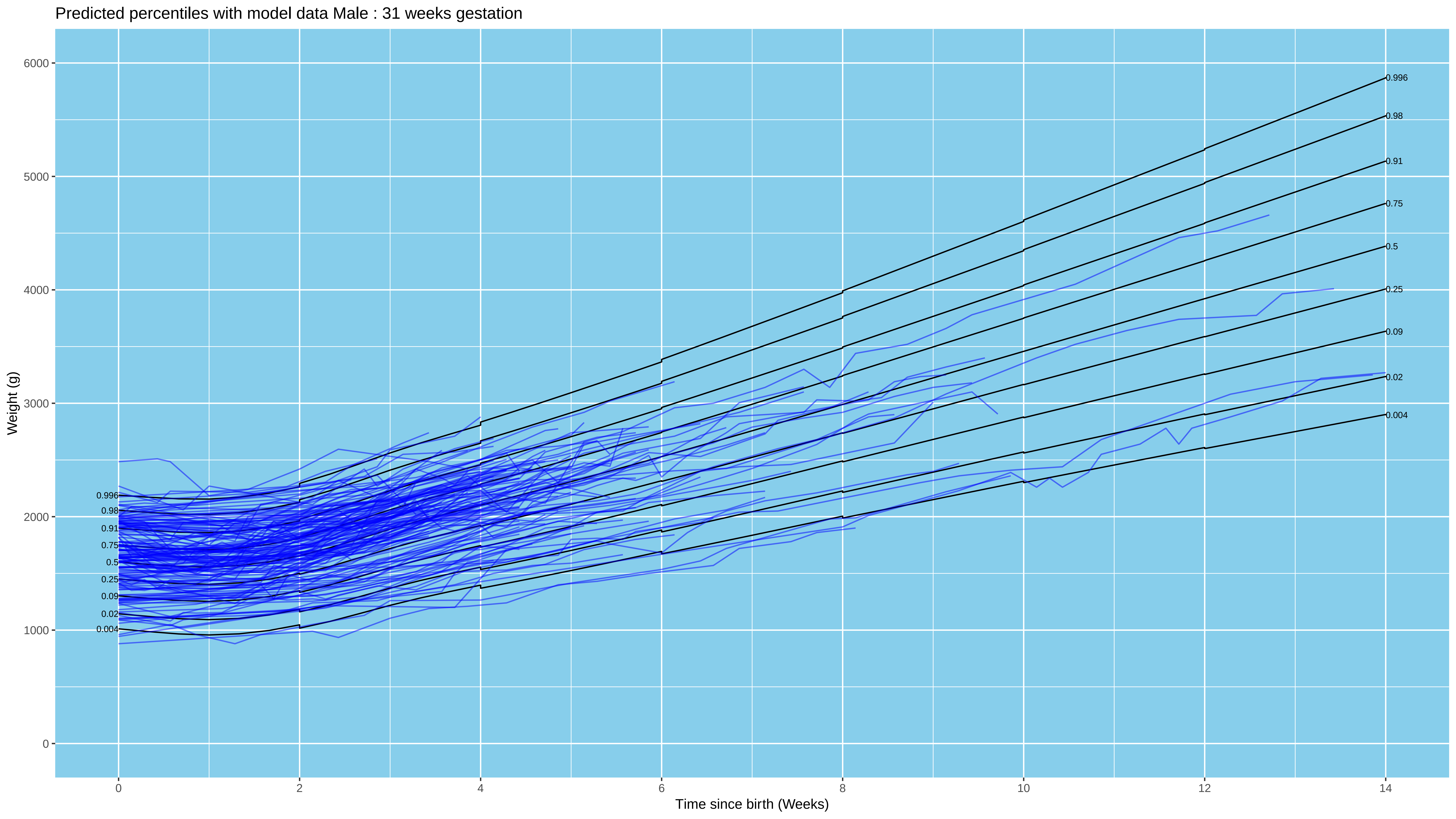
Predicted percentiles with model data Male : 31 weeks gestation
Weight (g)
Time since birth (Weeks)
0
1000
2000
3000
4000
5000
6000
0
2
4
6
8
10
12
14
0.996
0.98
0.91
0.75
0.5
0.25
0.09
0.02
0.004

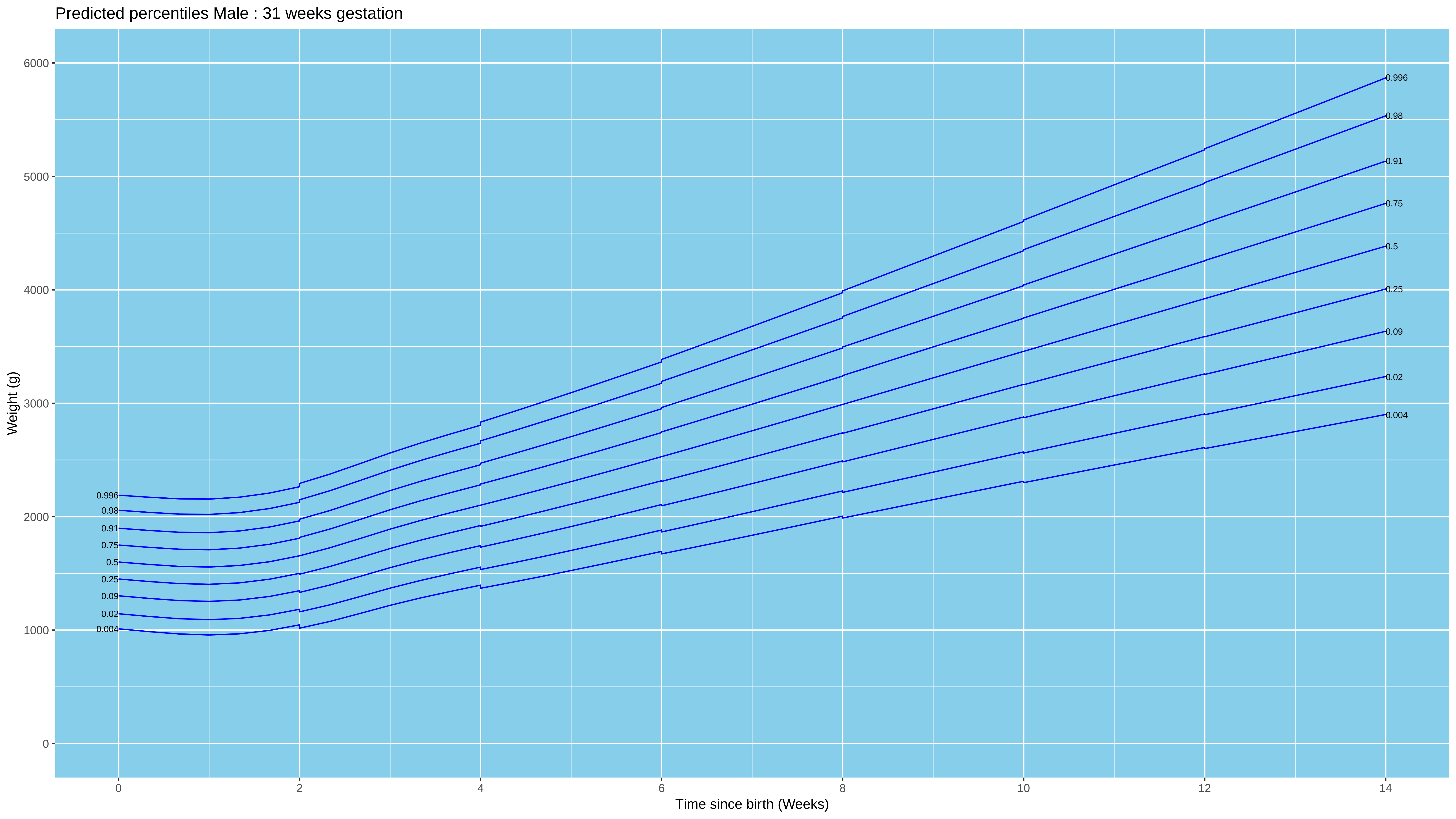

Predicted percentiles Male : 31 weeks gestation
Weight (g)
6000
5000
4000
3000
2000
1000
0
Time since birth (Weeks)
0
2
4
6
8
10
12
14
0.996
0.98
0.91
0.75
0.5
0.25
0.09
0.02
0.004

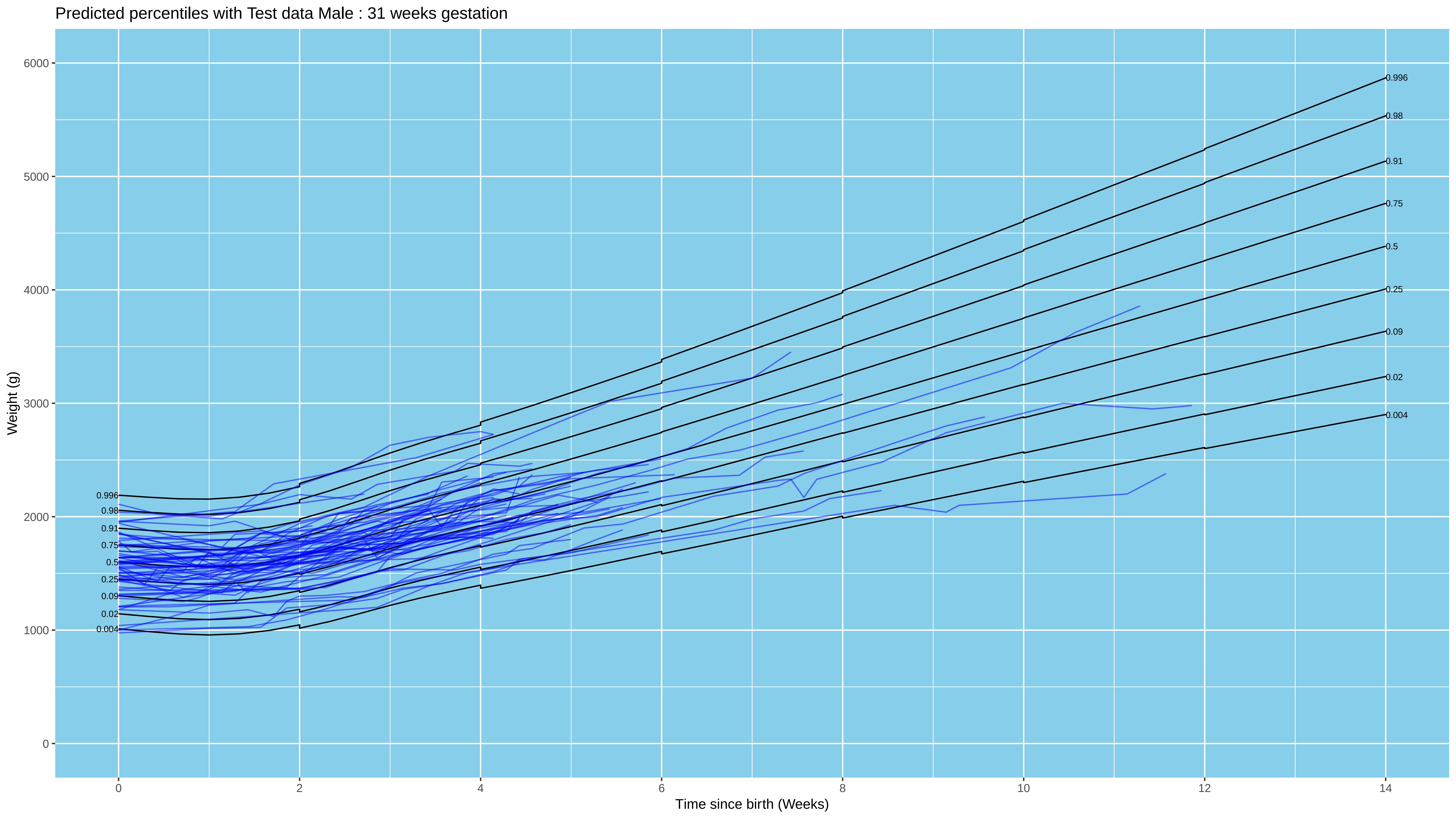

Predicted percentiles with Test data Male : 31 weeks gestation
Weight (g)
6000
5000
4000
3000
2000
1000
0
0
2
4
6
8
10
12
14
Time since birth (Weeks)
0.996
0.98
0.91
0.75
0.5
0.25
0.09
0.02
0.004

LMS percentiles with Test data Male : 31 weeks gestation

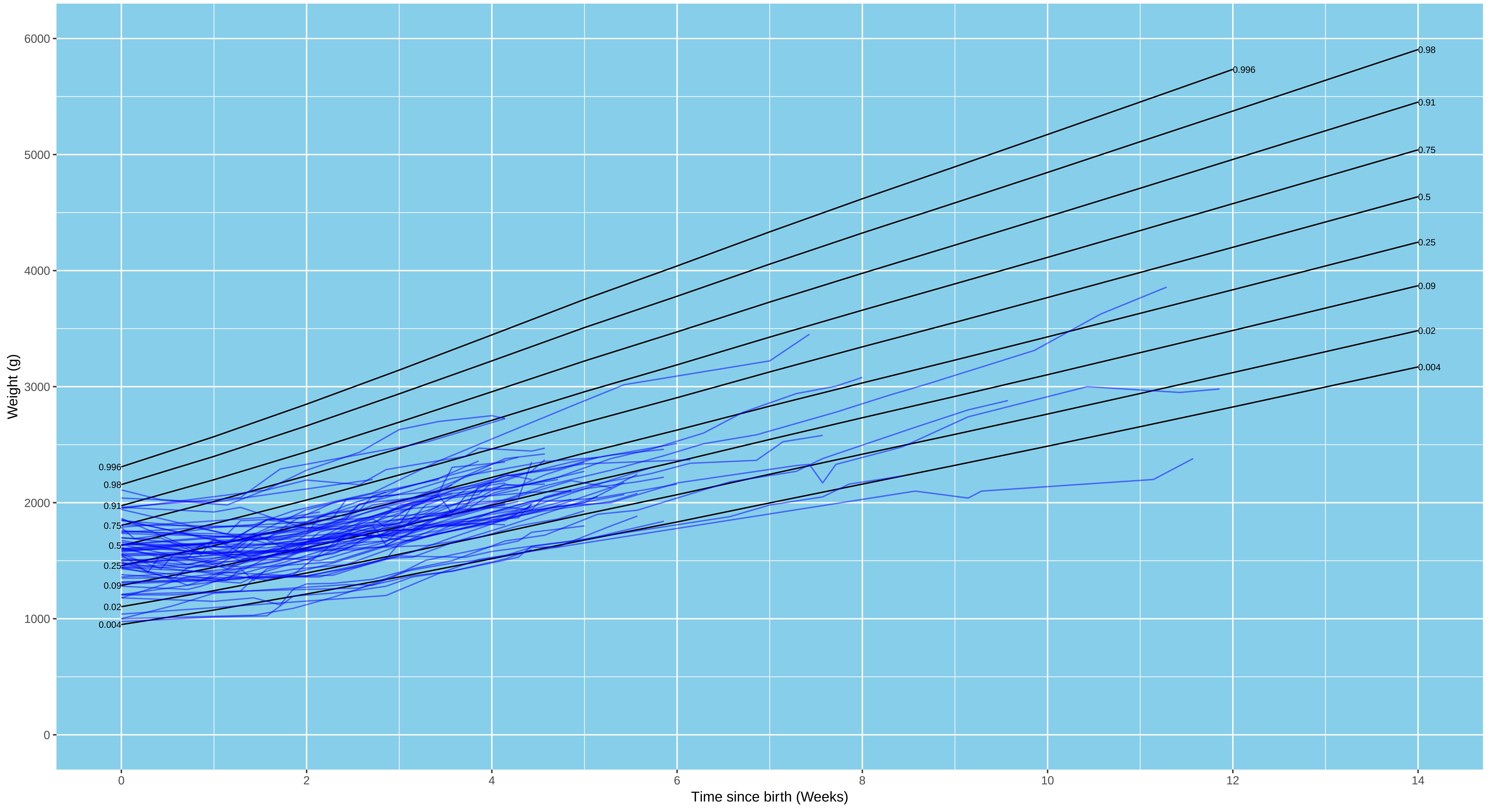

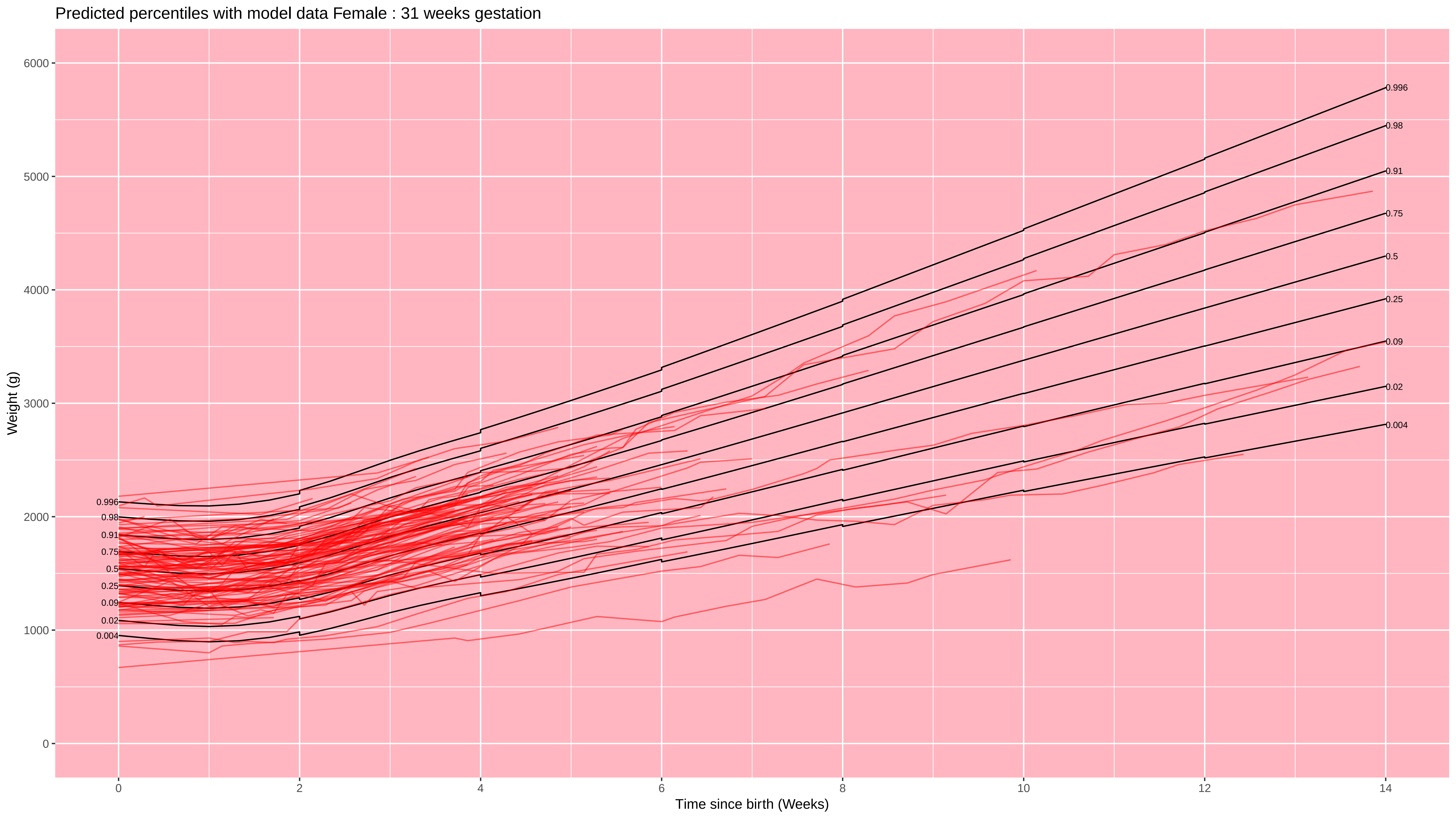
Predicted percentiles with model data Female : 31 weeks gestation
Weight (g)
Time since birth (Weeks)
0
1000
2000
3000
4000
5000
6000
0
2
4
6
8
10
12
14
0.996
0.98
0.91
0.75
0.5
0.25
0.09
0.02
0.004

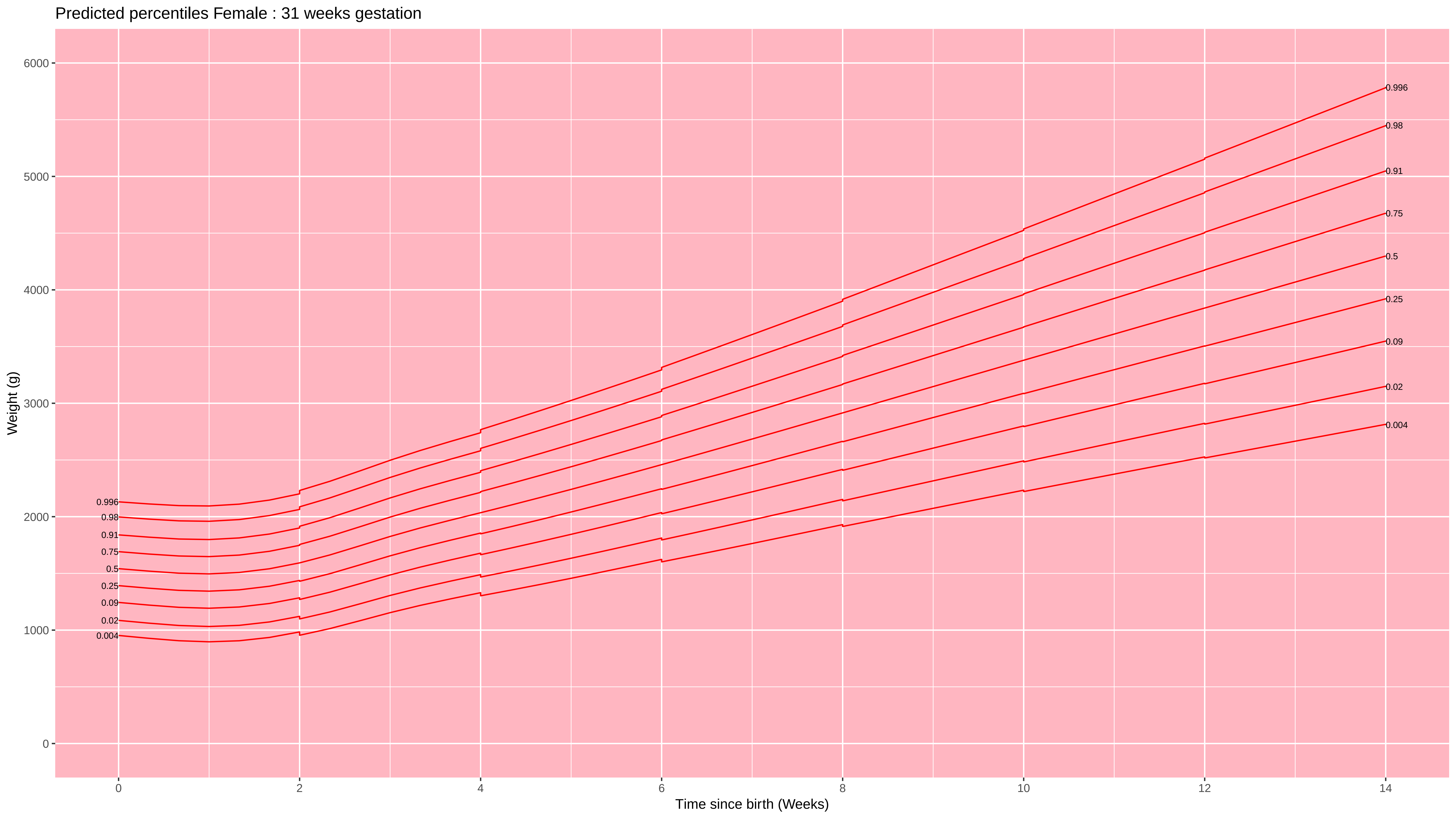

Predicted percentiles Female : 31 weeks gestation
Weight (g)
Time since birth (Weeks)
0
1000
2000
3000
4000
5000
6000
0
2
4
6
8
10
12
14
0.996
0.98
0.91
0.75
0.5
0.25
0.09
0.02
0.004

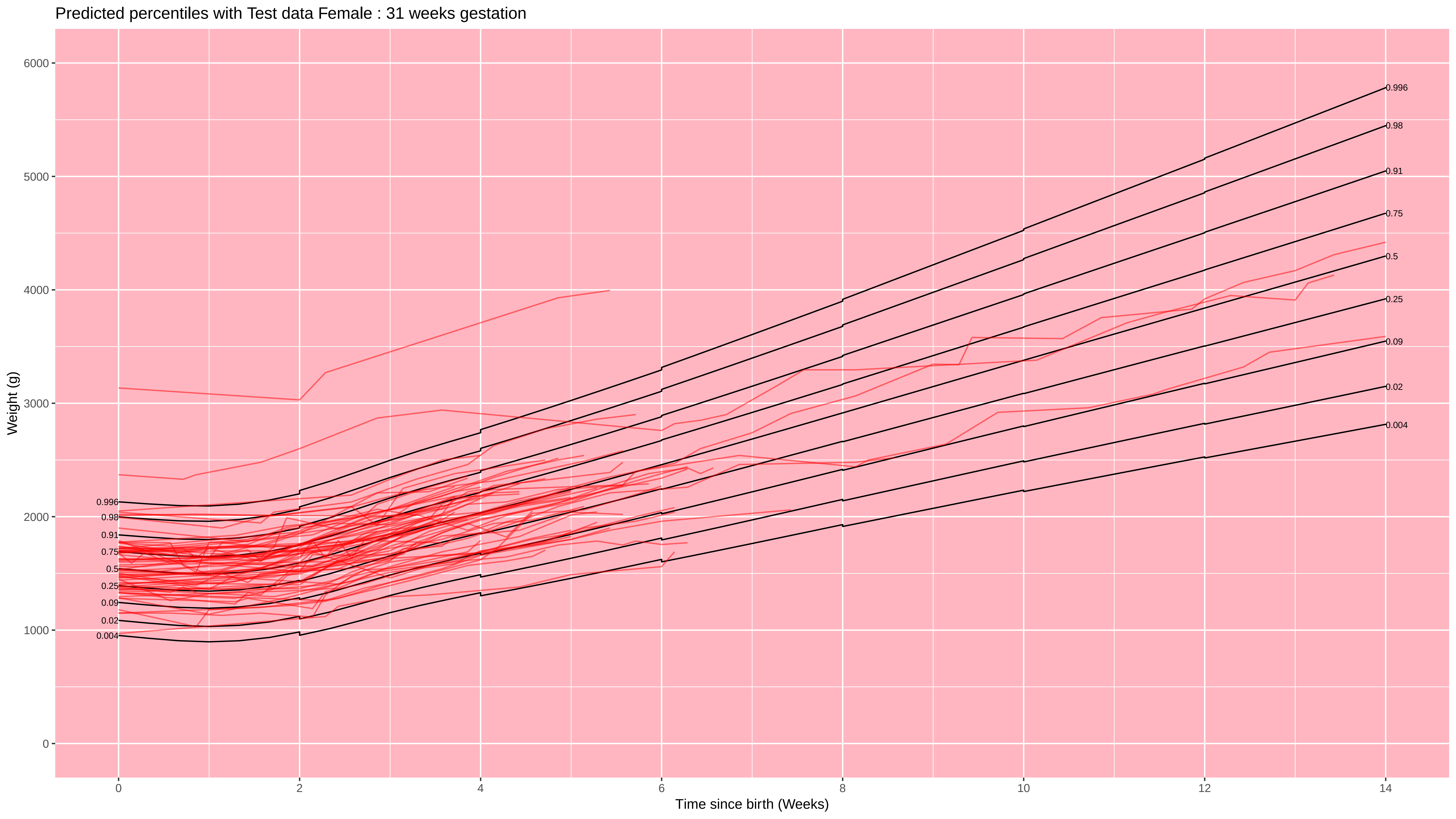

Predicted percentiles with Test data Female : 31 weeks gestation
Weight (g)
Time since birth (Weeks)
0
1000
2000
3000
4000
5000
6000
0
2
4
6
8
10
12
14
0.996
0.98
0.91
0.75
0.5
0.25
0.09
0.02
0.004

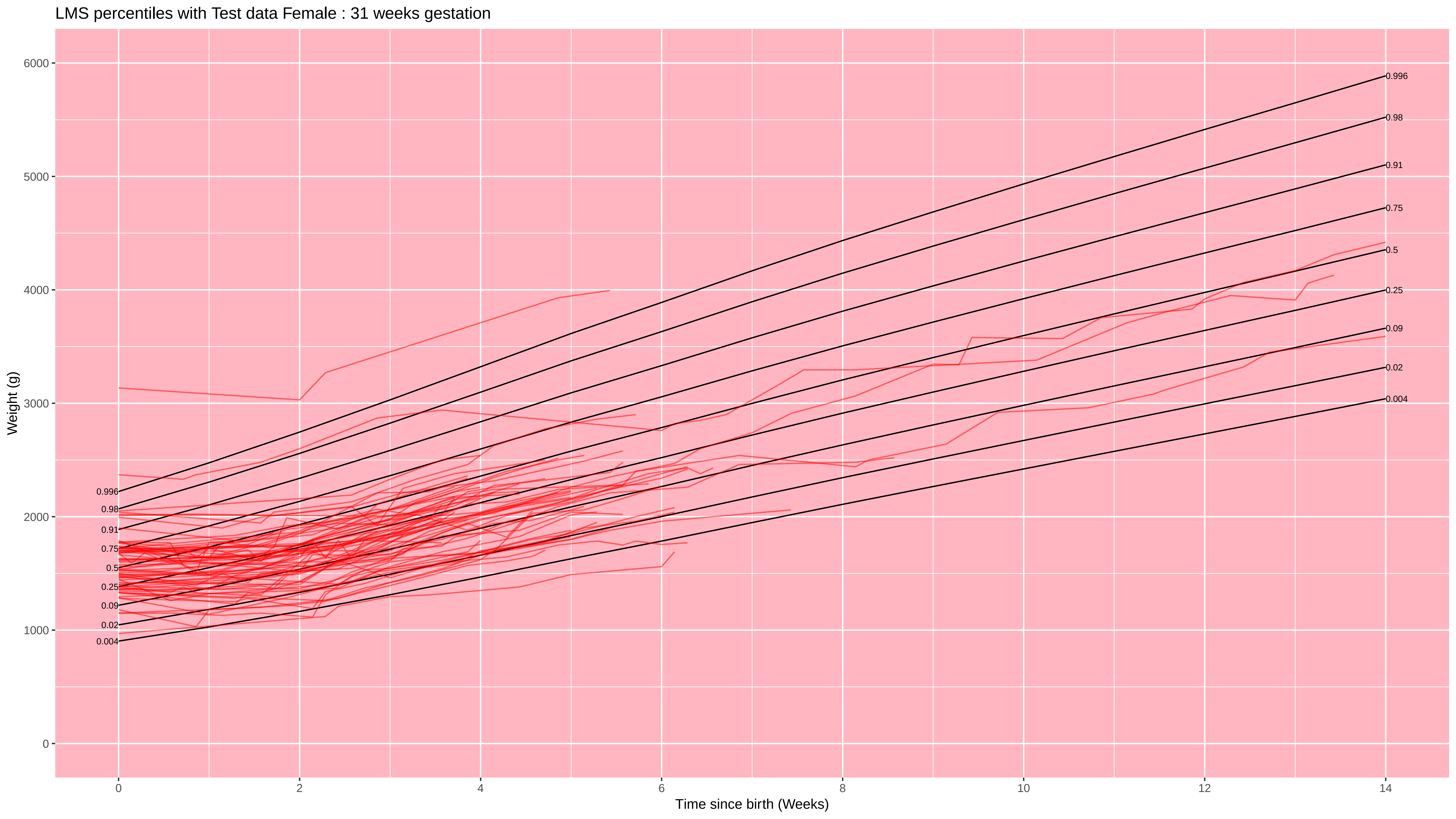

LMS percentiles with Test data Female : 31 weeks gestation
Weight (g)
6000
5000
4000
3000
2000
1000
0
0.996
0.98
0.91
0.75
0.5
0.25
0.09
0.02
0.004
0
2
4
6
8
10
12
14
Time since birth (Weeks)
